# Synthesis of an elusive, stable 2-azaallyl radical guided by electrochemical and reactivity studies of 2-azaallyl anions†

**DOI:** 10.1039/d0sc04822d

**Published:** 2021-02-03

**Authors:** Grace B. Panetti, Patrick J. Carroll, Michael R. Gau, Brian C. Manor, Eric J. Schelter, Patrick J. Walsh

**Affiliations:** Roy and Diana Vagelos Laboratories, Department of Chemistry, University of Pennsylvania 231 South 34th Street Philadelphia PA USA pwalsh@sas.upenn.edu schelter@sas.upenn.edu

## Abstract

The super electron donor (SED) ability of 2-azaallyl anions has recently been discovered and applied to diverse reactivity, including transition metal-free cross-coupling and dehydrogenative cross-coupling processes. Surprisingly, the redox properties of 2-azaallyl anions and radicals have been rarely studied. Understanding the chemistry of elusive species is the key to further development. Electrochemical analysis of phenyl substituted 2-azaallyl anions revealed an oxidation wave at *E*_1/2_ or *E*_pa_ = −1.6 V *versus* Fc/Fc^+^, which is ∼800 mV less than the reduction potential predicted (*E*_pa_ = −2.4 V *vs.* Fc/Fc^+^) based on reactivity studies. Investigation of the kinetics of electron transfer revealed reorganization energies an order of magnitude lower than commonly employed SEDs. The electrochemical study enabled the synthetic design of the first stable, acyclic 2-azaallyl radical. These results indicate that the reorganization energy should be an important design consideration for the development of more potent organic reductants.

## Abstract 2

The super electron donor (SED) capabilities of 2-azaallyl anions has recently been discovered and applied to diverse reactivity; their structures and electron transfer characteristics are reported herein.

## Graphical abstract

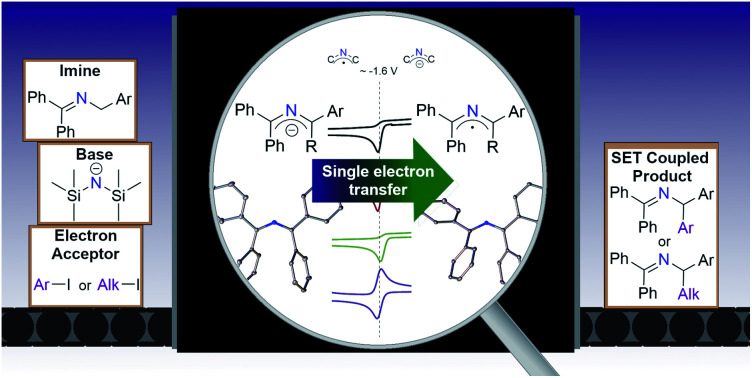

## Introduction

**Scheme 1:**
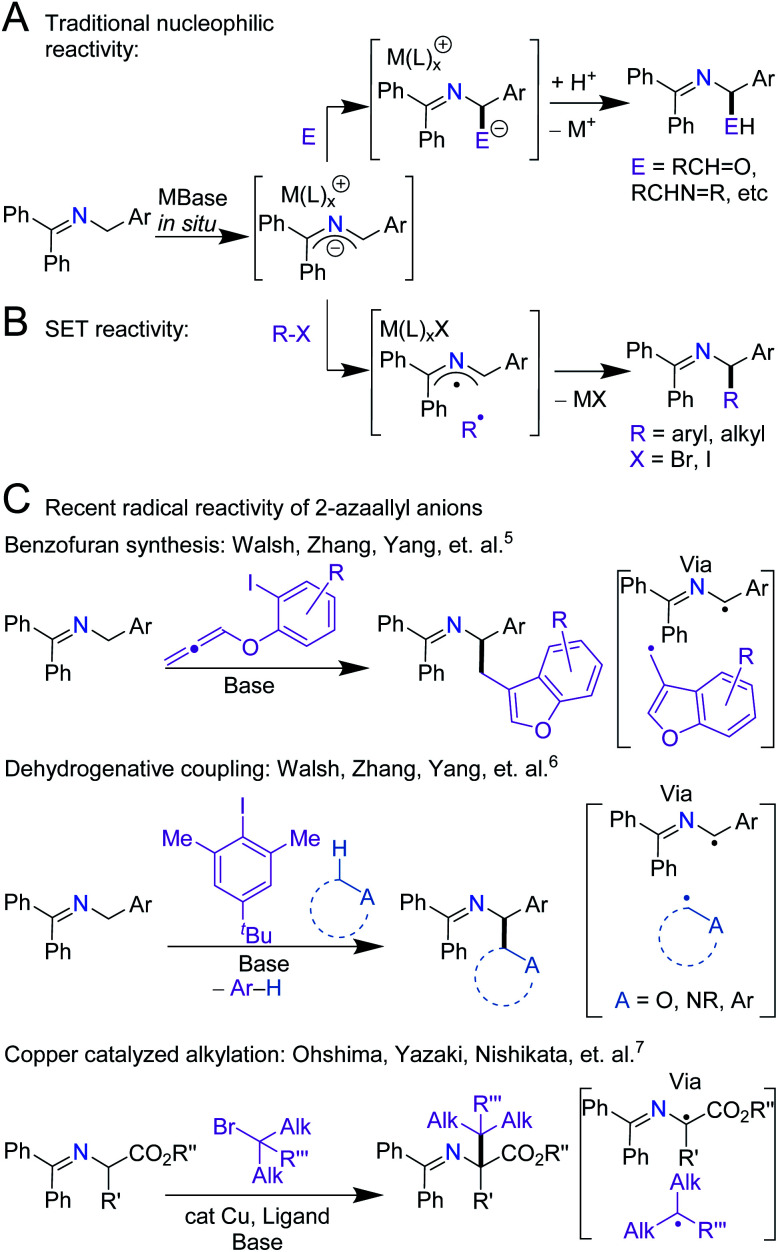
Divergent reactivity pathways reported for the phenyl substituted 2-azaallyl anion. (A) 2-Electron nucleophilic addition; E = aldehydes, imines, epoxides, carbon dioxide, *etc.* (B) An alternative 1-electron SET process; R–X = aryl halides and alkyl halides. (C) Representative recent reactivity employing the 2-azaallyl anion, exhibiting the importance of SET in its reactivity.^5–7^

**Scheme 2:**
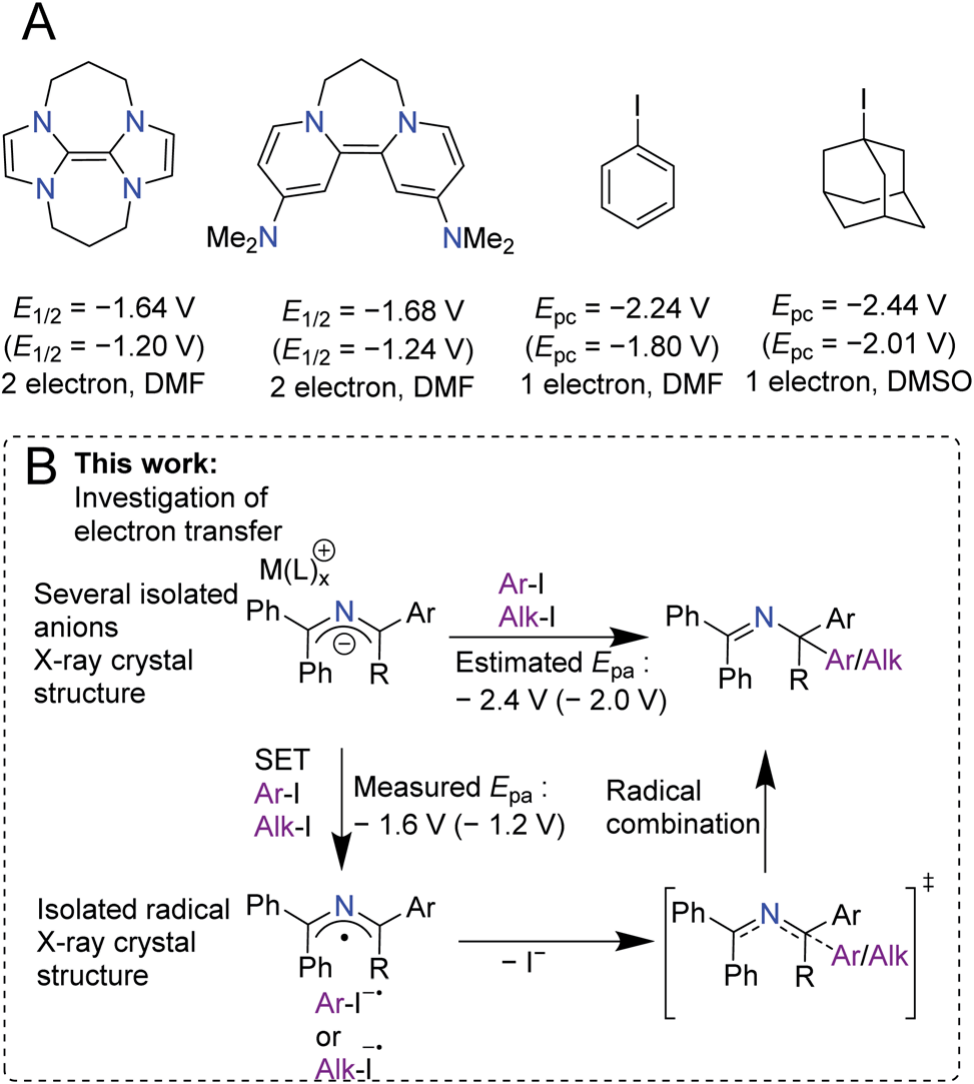
(A) Murphy and co-workers' reported SEDs and their electrochemical oxidation potentials. Also, the potentials of the electrophiles to be discussed in this work. The potentials are reported *versus* Fc/Fc^+^ and *versus* SCE in parentheses.^27–32^ The waves are listed with the number of electrons reported and the solvent the cyclic voltammetry was measured in. (B) This work, which provides isolation of 2-azaallyl anions and a 2-azaallyl radical, measurements of their electrochemistry, and investigation of their reactivities.

Over the last century, the diverse reactivity of 2-azaallyl anions has been mapped, with new and unexpected reactivity being discovered to date.^1–7^ Early in the inception of their chemistry, 2-azaallyl anions were used primarily as umpolung nucleophiles derived from imines.^8^ These intermediates react with a variety of electrophiles resulting in C–C bond formation (Scheme 1A).^4,7–12^ Recently, however, this umpolung reagent was found to activate electrophiles through one-electron processes (Scheme 1B). Using EPR spectroscopy, computational studies, and radical trapping experiments, our team demonstrated the activation of aryl, alkyl, and vinyl halides through single electron transfer (SET) pathways.^3,13,14^ We then applied the 2-azaallyl anion SET to a radical ring closing method for the synthesis of 3-aminoethyl benzofurans (Scheme 1C, top).^5^ A method for the dehydrogenative C(sp^3^)–C(sp^3^) coupling *via* a SET initiated radical relay (Scheme 1C, middle) was also outlined.^6^ Recently, Ohshima and coworkers introduced a copper-catalyzed synthesis of hindered amino acids by coupling 2-azaallyl radicals with alkyl radicals (Scheme 1C, bottom).^7^ Despite over 100 years of research into 2-azaallyl anions and their different forms of reactivity, electrochemical and structural data on these reactive species remain underdeveloped.^8,15–21^ Notably, electrochemical analysis of aryl and alkyl halide substrates reduced by 2-azaallyl anions reveal *E*_pc_ values approaching −2.4 V *versus* ferrocene (Fc/Fc^+^) (−2.0 V *vs.* SCE).^22^ The SET reactivity observed with 2-azaallyl anions is reminiscent of organic super electron donors (SEDs), which have garnered attention due to their potential advantages over transition metal reducing agents, including lower costs and toxicities (Scheme 2A). Additionally, SEDs have been successfully developed for a variety of applications, including replacement of stoichiometric metal reducing agents and use in redox flow batteries.^23–25^ To develop highly-reducing organic compounds, chemists have designed SEDs that become aromatic upon electron transfer (Scheme 2A). As a result, reported SEDs have attained reduction potentials approaching *E*_1/2_ = −1.9 V *versus* Fc/Fc^+^ (*E*_1/2_ = −1.5 V *vs.* SCE) in DMF.^26,27^ However, even SEDs with potentials as low as −1.3 V *versus* Fc/Fc^+^ (−0.8 V *vs.* SCE) have shown reactivity with aryl and alkyl iodide substrates.^26,28^ The high energy barrier of electron transfer (uphill by 0.6–0.8 V) is proposed to be overcome by the irreversibility of iodide anion loss from the radical anions.^29–31^ With the interesting reactivity of the 2-azaallyl anion, and their poorly-defined structural and electrochemical properties, we set out to investigate the electron transfer behavior of phenyl substituted 2-azaallyl anions.

Herein we describe the structures and electron transfer characteristics of isolated 2-azaallyl anions. Surprisingly, the experimentally determined oxidation events occur at less negative potentials than expected from benchmarking in our previous reactivity studies (Scheme 2B).^3,8,14,22^ To further investigate this electrochemical event, isolation and full characterization of several 2-azaallyl anions and the corresponding oxidation product, the 2-azaallyl radical, were performed. Single crystal XRD data of these species revealed only small changes in the overall structures between redox states, indicating a hidden feature of the phenyl substituted 2-azaallyl anions: their low activation energies of SET.

## Results and discussion

**Fig. 1:**
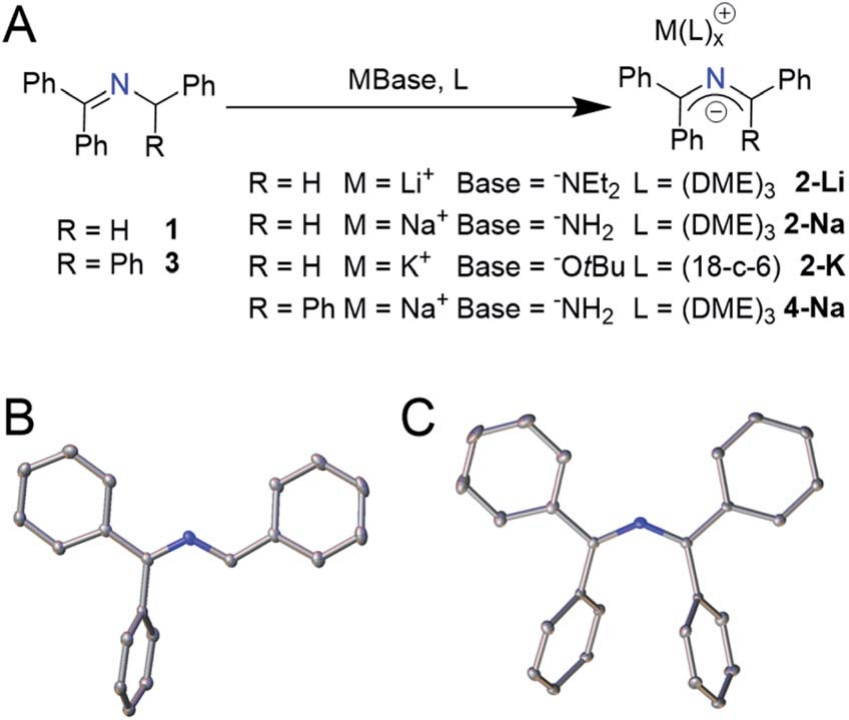
(A) Synthetic scheme of 2-azaallyls **2-Li**, **2-Na**, **2-K** and **4-Na** from the corresponding imines. (B) Thermal elipsoid plots of [Na(DME)_3_][1,3,3-triphenyl-2-azaallyl anion] **2-Na** at the 30% probability level, representative of **2-Li** and **2-K**. Phenyl ring hydrogen atoms and the counter cation removed for clarity. (C) Thermal elipsoid plots of [Na(DME)_3_][1,1,3,3-tetraphenyl-2-azaallyl anion] **4-Na** at the 30% probability level. Hydrogen atoms and the counter cation removed for clarity.

Initial studies into *in situ* generated 2-azaallyl anions were complicated by irreproducibility and unexpectedly high numbers of waves observed in electrochemical experiments. To address these issues, we sought to first isolate the 2-azaallyl anions and then study these reactive species. We chose a series of weakly nucleophilic bases with low-boiling point conjugate acids to facilitate the synthesis and characterization of the proposed intermediates. We found that treating ketimine **1** with a slight excess of lithium diethyl amide (LiNEt_2_) in 1,2-dimethoxyethane (DME) yielded an intense purple solution (Fig. 1A). This purple compound was determined to be the expected lithium 2-azaallyl anion DME solvate **2-Li**, isolated in 82% crystalline yield. Sodium amide base (NaNH_2_) generated a similar dark purple solution upon reaction with **1**, albeit more slowly. From this solution we isolated the analogous sodium 2-azaallyl anion **2-Na** in 76% crystalline yield. An alternative, rational synthesis of **2-K** was realized employing potassium *tert*-butoxide (KO^*t*^Bu) as the base, providing 90% crystalline yield of **2-K** from **1**. With the goal of studying the electrochemistry, we sought to prepare a 2-azaallyl anion more likely to exhibit reversible oxidation. We rationalized that the 1,1,3,3-tetraphenyl 2-azaallyl anion would be sterically protected from dimerization, a commonly observed side reaction in 2-azaallyl chemistry.^8,13,14^ Thus, deprotonation of the imine *N*-benzhydryl-1,1-diphenylmethanimine (**3**) using NaNH_2_ generated **4-Na** in analogy to the synthesis of **2-Na**. All 2-azaallyl anions were isolated as beautiful, strongly absorbing purple crystalline solids (*ε* = 14 000 mol^−1^ cm^−1^ L, Fig. S21†) that were stable under inert atmosphere. These anions, however, rapidly decolorized and decomposed upon exposure to trace oxygen or water.

Single crystal X-ray diffraction (SCXRD) was performed on all four isolated 2-azaallyl anions. X-ray diffraction quality crystals of compounds **2-Li**, **2-Na**, and **4-Na** grew from cooled, concentrated solutions of DME layered with pentane, while compound **2-K** crystallized from hot concentrated solutions of toluene upon cooling to room temperature. All compounds were found to have two phenyl rings that were almost perfectly co-planar with the 2-azaallyl moiety, while the other phenyl rings rotated out of conjugation (Fig. 1B and C). Compounds **2-Li**, **2-Na**, and **2-K** all have a similar 2-azaallyl anion core, with a C–N–C angle of 123.9(8)–124.46(16)°, while **4-Na** has an expanded angle of 131.61(11)–132.84(11)°. We suspect that this increased angle is due to the steric repulsion of the two internal phenyl rings.

**Scheme 3:**
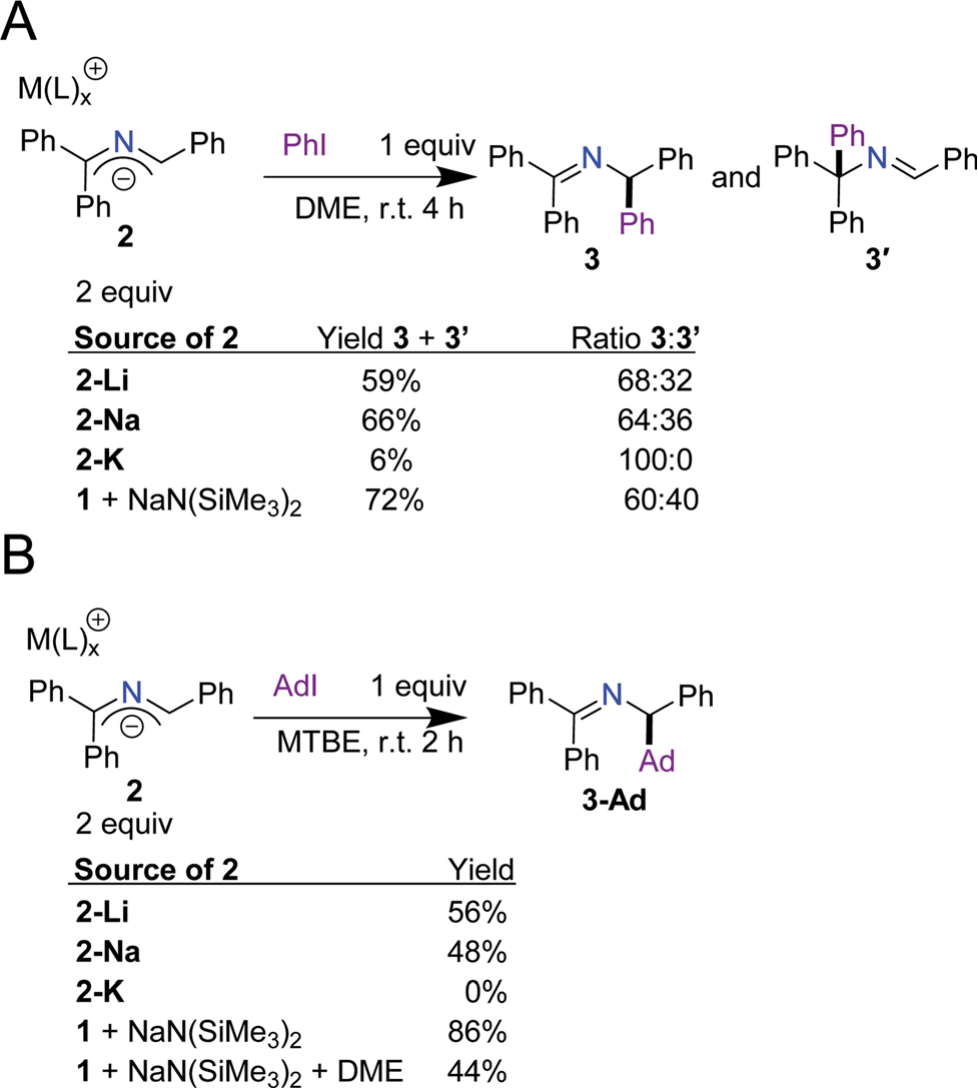
(A) Comparison of the reaction products of the 2-azaallyl anion and phenyl iodide using isolated **2-M** and *in situ* generated **2-Na** to produce **3** and **3′**. (B) Comparison of the reaction products of the 2-azaallyl anion and adamantyl iodide using isolated **2-M** and *in situ* generated **2-Na** with varying amounts of dimethoxyethane (DME) to produce **3-Ad**.

Next, we aimed to (1) validate our hypothesis that arylation and alkylation occur *via* direct SET between the anions and aryl and alkyl iodides (Scheme 2B) and (2) determine if any cation effects are operative. By following our previously optimized conditions and replacing NaN(SiMe_3_)_2_ and imine **1** with the 2-azaallyl anions (**2-M**), we compared the reactivity of the isolated anions with the *in situ* generated anions.^14^ When phenyl iodide was treated with 2 equiv. of **2-M** in DME, the yields of **3** and **3′** were 59% for **2-Li**, 66% for **2-Na**, and 6% for **2-K**. The yields for **2-Li** and **2-Na** fit well with our results with *in situ* generation of 2-azaallyl anions from ketimine **1** and NaN(SiMe_3_)_2_ (72%, Scheme 3A). The yields of **3-Ad** from the reaction of adamantyl iodide with either **2-Li** or **2-Na** were 56% and 48%, respectively. The *in situ* generated anion from **1** and NaN(SiMe_3_)_2_ (Scheme 3B) produced the expected alkylated product in 86% yield. The higher yield from the *in situ* generated 2-azaallyl anion may be due, in part, to the cations of **2-Li** and **2-Na** being bound by DME rather than the reaction solvent, methyl *tert*-butyl ether (MTBE). To test this hypothesis, we modified our *in situ* conditions by adding 3 equiv. of DME per sodium cation (9 equiv. total) to the reaction medium, resulting in a reduction of the yield of **3-Ad** to 44% (Scheme 3B). This observation compares well with the use of isolated **2-Na** (48% yield). Due to the similarity in yields between the isolated 2-azaallyl anions and those prepared *in situ*, we postulated that **2-Li** and **2-Na** are the active species in the SET arylation and alkylation chemistry, acting as effective room temperature SEDs. Despite the success of **2-Li** and **2-Na**, we found that isolated **2-K** does not perform well under the reaction conditions. We attribute this decrease in yield primarily to solubility issues, as **2-K** is sparingly soluble in both MTBE and DME. With the knowledge that the isolated 2-azaallyl anions are competent intermediates in our previous chemistry (Scheme 2B), we next examined their electrochemistry.

**Fig. 2:**
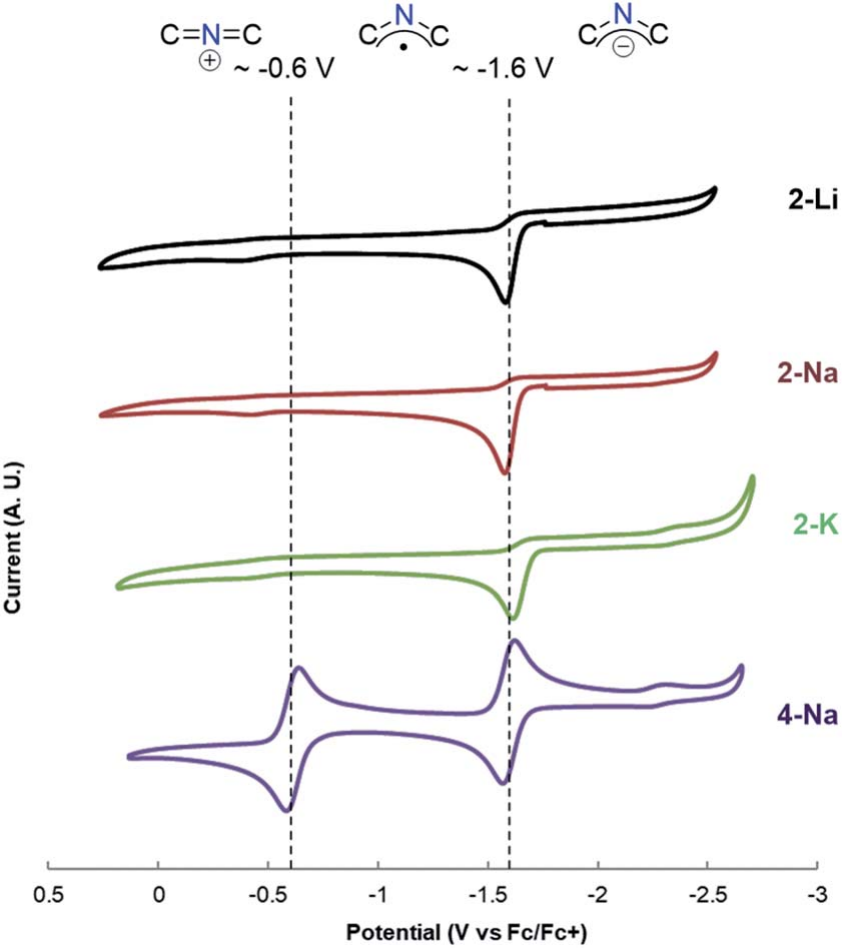
Cyclic voltammetry of compounds **2-Li**, **2-Na**, **2-K**, and **4** with guides for the eye to indicate the redox state regimes of the 2-azaallyl compound. Solvent = DME, *ν* = 100 mV s^−1^, [sub] = 0.001 M, [^*n*^NPr_4_][BAr^F^_4_] = 0.1 M. The signal observed at −2.3 *versus* Fc/Fc^+^ is the redox feature of small quantities of the imine decomposition product.

To mimic the conditions in our prior arylation chemistry (Scheme 2B), cyclic voltammetry of the 2-azaallyl anions was performed in DME. Compounds **2-Li**, **2-Na**, and **2-K** exhibit effectively the same cyclic voltammetry, with one irreversible oxidation wave at *E*_pc_ = −1.61 V *versus* the Fc/Fc^+^ couple (*E*_pc_ = −1.1 V *versus* S.C.E.) (Fig. 2). The irreversibility is consistent with a fast and irreversible dimerization process following oxidation, in line with our previous observations (see discussion below).^13,14^ While measuring the electrochemistry of **4-Na**, we observed two reversible one electron waves *E*_1/2_ = −1.62 V and −0.61 V *versus* the Fc/Fc^+^ couple (−1.11 and −0.1 V *versus* S.C.E.) (Fig. 2), in line with our hypothesis that the radical derived from **4-Na** would possess greater lifetime. We assign these electrochemical events to 2-azaallyl anion oxidation to the 2-azaallyl radical (*E*_1/2_ = −1.61 V *vs.* Fc/Fc^+^) and 2-azaallyl radical oxidation to the 2-azaallenium cation (*E*_1/2_ = −0.61 V *vs.* Fc/Fc^+^). While the *E*_pc_ indicates that 2-azaallyl anions are moderately strong reductants, they are not as strong as many of the reported SEDs (Scheme 2A).^27^ Due to the reactivity of the 2-azaallyl anion with aryl and alkyl iodides, we expected a significantly more negative reduction potential. To further probe the electron transfer of 2-azaallyl anions we turned to chemical oxidants to characterize the products of 1 electron oxidation.

**Fig. 3:**
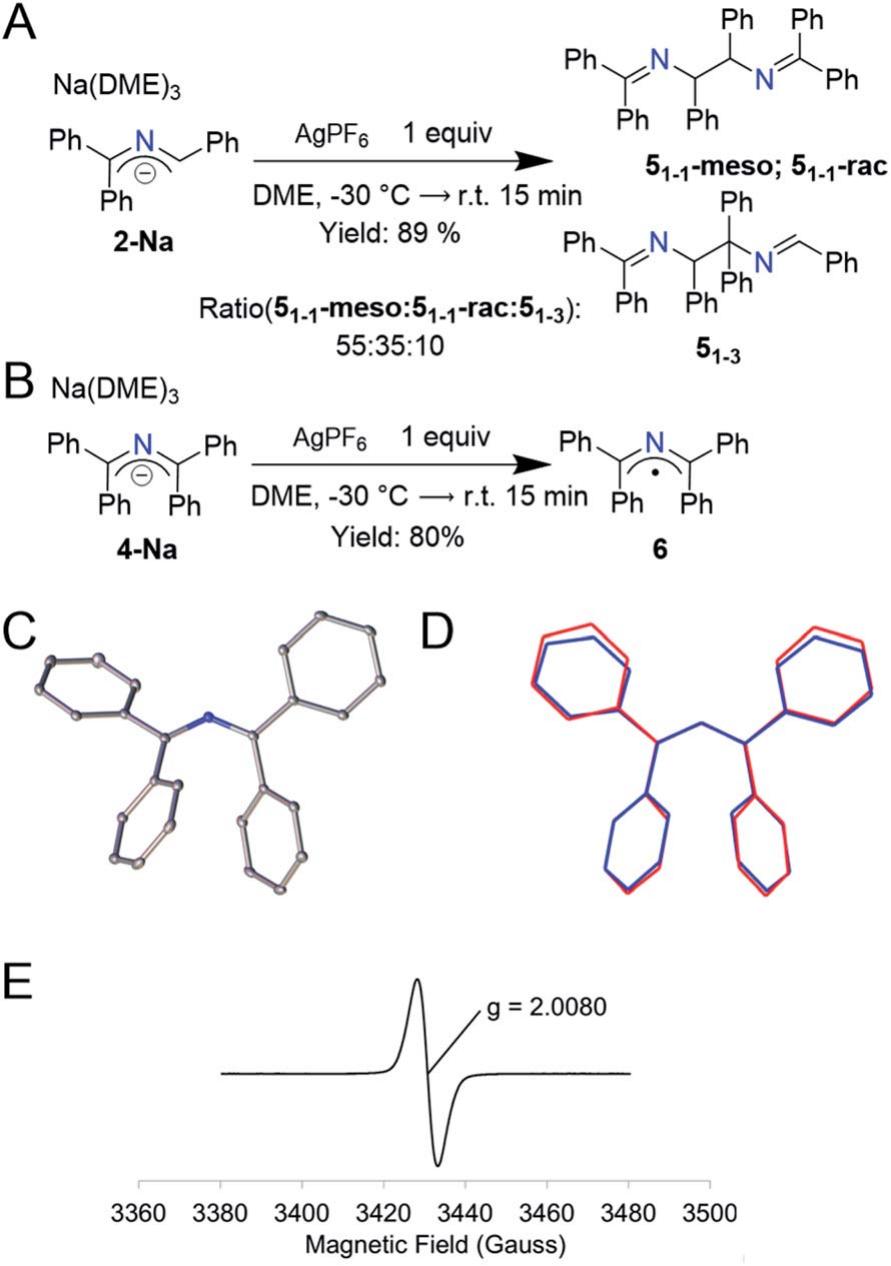
(A) 1-Electron oxidation of **2-Na** by AgPF_6_ yielding the dimers **51-1-rac**, **51-1-meso**, and **51-3** in 89% yield. (B) 1-Electron oxidation of **4-Na** by AgPF_6_ yielding compound **6** showing the redox stability of **4-Na**. (C) Thermal elipsoid plots of [1,1,3,3-tetraphenyl-2-azaallyl radical] **6** at the 30% probability level. Hydrogen atoms removed for clarity. (D) Wireframe overlay of **4-Na** and **6** depicting the structural similarity. (E) Experimental X-band EPR spectra of **6** at 298 K dissolved in benzene (6 mM) (perpendicular mode; *g* = 2.0080; *ν* = 9.64 GHz; *P* = 0.0201 mW; modulation amplitude = 0.2 mT).

Based on the electrochemistry above, we searched for appropriate 1-electron chemical oxidants and found that Ag^+^ gave the cleanest oxidation. Addition of 1 equiv. of AgPF_6_ to a purple solution of **2-Na** at −30 °C resulted in a yellow solution and a black solid (likely Ag^0^). ^1^H NMR analysis of the organic components indicated a clean conversion to a mixture of 2-azaallyl dimers **5** in 89% yield (Fig. 3A, 2 : 3 ratio of **51-1-rac** to **51-1-meso**, similar to that observed in arylation reactions).^14^ When running the LCMS of this mixture, we observed a third peak with the same mass as well as peaks indicative in the NMR of a third previously undescribed dimer. We propose that this species is **51-3**, which was not previously reported due to the low concentrations of 2-azaallyl dimers that are normally observed. We propose that the irreversibility of the electrochemistry in Fig. 2 is a result of the facile dimerization of the triphenyl 2-azaallyl radicals. To slow the dimerization processes, we moved to the more sterically encumbered **4-Na**. Thus, addition of 1 equiv. of AgPF_6_ to **4-Na** produces an NMR silent, green solution along with a similar black solid that was filtered away (Fig. 3B). X-ray quality crystals of the green compound were grown from a cooled, saturated solution of acetonitrile, revealing the compound to be 1,1,3,3-tetraphenyl 2-azaallyl radical (**6**). EPR analysis of **6** in benzene exhibited a broad signal centered at *g* = 2.0080. The only other reported acyclic 2-azaallyl radical dimerizes over the course of hours in solution, precluding isolation.^32^ Importantly, the structure of radical **6** is similar to anion **4-Na** with the only statistically significant difference being a slight decrease of the C–N–C angle from 131.61(11)–132.84(11)° to 128.87(14)° (Fig. 3C and D) within the 2-azaallyl core. With knowledge of this minor structural change, we shifted our focus to computationally probing electron transfer in the 2-azaallyl anions.

**Scheme 4:**
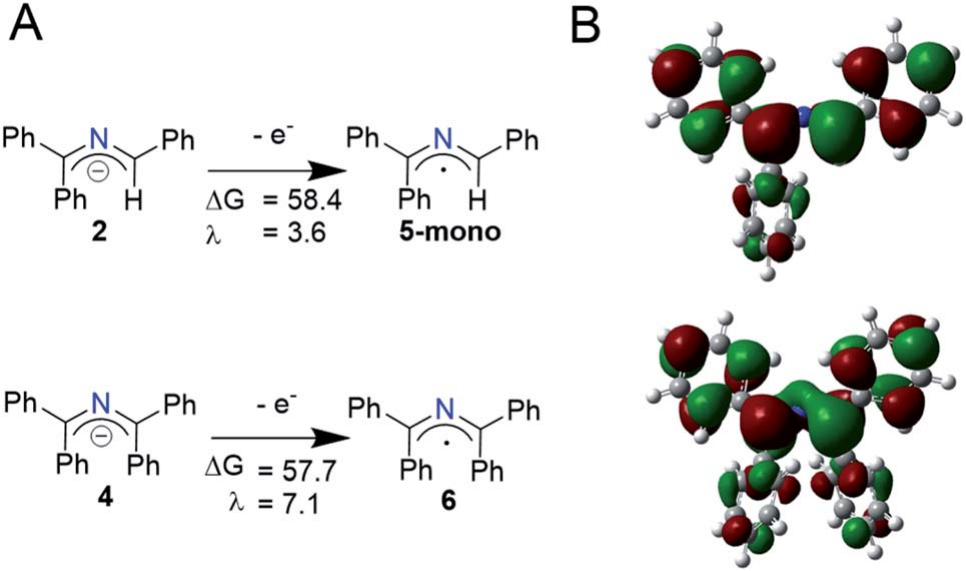
(A) Computed half reaction free energies (Δ*G*) and reorganization energies (*λ*) of the oxidation of free anions **2** (top) and **4** (bottom). Values are in kcal mol^−1^ and calculated using a solvent field of 1,4-dioxane. (B) Kohn–Sham orbitals of the HOMO of **2** and **4** where the non-bonding character of the orbital is shown.

Using a simplified version of Marcus–Hush theory, a method first described by the Nelsen group and extensively used by Murphy and coworkers, we probed the electron transfer barriers of the 2-azaallyl anion.^24,33,34^ This simplified model allows for facile comparison of the transition state energy (ΔG*) of different outer sphere electron transfer processes using the reorganization energies (*λ*) and the free energy of the electron transfer (Δ*G*_rel_). We optimized the structures of 2-azaallyl anions **2** and **4** and 2-azaallyl radicals **5-mono** and **6** (Scheme 4) using the B3LYP functional, the 6-31+G(d,p) basis set, and a CPCM solvent field. The reorganization energy of the 2-azaallyl anions **2** and **4** upon loss of an electron are remarkably low (1.6–7.8 kcal mol^−1^; Schemes 4A and S1†). This low energy cost is likely due to the orbital that the electron is removed from. In the case of 2-azaallyl anions, the orbital is one with primarily nonbonding character, which leads to minimal differences in the energy surfaces before and after electron transfer (Scheme 4B). While the reorganization energies suggest rapid reactivity, this should be regarded as a simplified model as it does not account for other factors affecting electron transfer rate, *e.g.* donor–acceptor electronic coupling, donor–acceptor association, *etc.*^35–37^ We are planning on investigating these factors affecting electron transfer kinetics in future work.

## Conclusions

We have described the electron transfer properties of the 2-azaallyl anion and provided insight into its ability to activate difficult substrates *via* 1-electron transfer at room temperature. By providing the first X-ray structure of a stable 2-azaallyl radical, we demonstrated that the structures of the 2-azaallyl anion and the 2-azaallyl radical are nearly identical by both X-ray crystallography and DFT calculations. We attribute this to the 2-azaallyl anion's HOMO having nonbonding character. This similarity in the structures lead to the computational determination of a remarkably small reorganization energy. This study highlights the importance of reorganization energy as a compliment to reduction potential, as a guiding principle in the design of SEDs.

## Conflicts of interest

There are no conflicts of interest to declare.

## Supplementary Material

SC-012-D0SC04822D-s001

SC-012-D0SC04822D-s002
