# Supplementary material for: Synthesis of an elusive, stable 2-azaallyl radical guided by electrochemical and reactivity studies of 2-azaallyl anions
Source: Chem Sci. 2021 Feb 3;12(12):4405–10. doi: 10.1039/d0sc04822d (PMC8179533; doi:10.1039/d0sc04822d)
Supplement: SC-012-D0SC04822D-s001 [file SC-012-D0SC04822D-s001.pdf]

Electronic Supplementary Material (ESI) for Chemical Science.
This journal is © The Royal Society of Chemistry 2021

Synthesis of an elusive, stable 2-azaallyl radical guided by electrochemical and reactivity studies of 2-azaallyl anions

Grace B. Panetti,[a] Patrick J. Carroll,[a] Michael R. Gau,[a] Brian C. Manor,[a] Eric J. Schelter,*[a] and Patrick J. Walsh*[a]

[a] P. Roy and Diana T. Vagelos Laboratories, Department of Chemistry, University of Pennsylvania, Philadelphia, PA 19104, United States

Phone: (+1)-(215)-898-8633, (+1)-(215)-573-2875;

* e-mail: schelter@sas.upenn.edu, pwalsh@sas.upenn.edu

Supporting Information

Experimental Procedures S2
Synthetic Procedures S4
^{1}H and ^{13}C NMR Data S10
Electrochemical Data S36
Electronic Spectra S45
X-Ray Crystal Structures S46
Computational Data S52
References S75

Experimental Procedures

General Methods:

For all reactions and manipulations performed under an inert atmosphere (N_2), standard Schlenk techniques or a Vacuum Atmospheres, Inc. Nexus II drybox equipped with a molecular sieves 13X / Q5 Cu–0226S catalyst purifier system were used. Glassware was oven-dried overnight at 150 °C prior to use. ^{1}H NMR spectra were obtained on a Brüker AM-500 or a Brüker UNI-400 Fourier transform NMR spectrometer at 500 or 400 MHz, respectively. ^{13}C{^{1}H} NMR spectra were recorded on a Brüker AM-500 Fourier transform NMR spectrometer at 126 MHz. All spectra were measured at 300 K unless otherwise specified. Chemical shifts were recorded in units of parts per million (ppm) downfield from residual proteo solvent peaks (^{1}H) or characteristic solvent peaks (^{13}C{^{1}H}). All coupling constants are reported in hertz. EPR spectra were acquired in perpendicular mode on a Brüker EMX X-band EPR spectrometer equipped with an Oxford cryostat with a liquid helium dewar at Temple Universtiy. Elemental analyses were obtained on a Costech ECS 4010 instrument at the Earth and Environmental Science department of the University of Pennsylvania.

Solvents:

Dimethoxyethane, pentane, diethyl ether, toluene, and acetonitrile and pentane were purchased from Fisher Scientific. The solvents were sparged for 20 min with dry N_2 and dried using a commercial two-column solvent purification system comprising columns packed with Q5 reactant and neutral alumina respectively (for hexanes and pentane), or two columns of neutral alumina (for THF). Dry methyl *tert*-butyl ether was further dried over calcium hydride, vacuum transferred, and stored over molecular sieves before use. Deuterated tetrahydrofuran, chloroform and dichloromethane were purchased from Cambridge Isotope Laboratories, Inc. Deuterated tetrahydrofuran was stored for at least 12 h over potassium mirror prior to use. 1-2 dichloroethane was purchased from Fisher Scientific and used as received.

Materials:

Benzophenone imine was purchased from Alfa-Aesar and used as received. Benzyl amine was purchased from Acros Organics and used as received. Benzhydryl amine was purchased from Tokyo Chemical Industries and used as received. $NaN(SiMe_3)_2$ was purchased from Sigma-Aldrich and used as received. KOtBu was purchased from Acros Organics and used as received. 18-crown-6 was purchased from Acros Organics and purified by precipitation of the MeCN adduct, followed by drying overnight at r.t. under reduced pressure. The solid was then further dried by dissolving in Et_2O with 4 Å molecular sieves for 2 days. After which the solution was filtered and the volatiles removed under reduced pressure. Adamantyl iodide (AdI) was

purchased from Sigma-Aldrich and sublimed before use. Phenyl iodide (PhI) was purchased from Acros Organics and distilled before use. $LiNEt_2$ was prepared from previously reported synthesis.[1]

Electrochemistry:

Voltammetry experiments (CV, DPV) were performed using a CH Instruments 620D Electrochemical Analyzer/Workstation and the data were processed using CHI software v9.24. All experiments were performed in an N2 atmosphere drybox using electrochemical cells that consisted of a 4 mL vial, glassy carbon working electrode, a platinum wire counter electrode, and a silver wire plated with AgCl as a quasi-reference electrode. The quasi-reference electrode was prepared by dipping a length of silver wire in concentrated hydrochloric acid. The working electrode surfaces were polished prior to each set of experiments. Potentials were reported versus ferrocene, which was added as an internal standard for calibration at the end of each run. Solutions employed during these studies were ~2 mM in analyte and 100 mM in $[^{n}Pr_4N][BAr^F]$ in 2 mL of dimethoxyethane. All data were collected in a positive-feedback IR compensation mode.

UV-visable Spectroscopy:

10 mm path length quartz cells fused with a J-Young valve were used for UV-vis of air and moisture sensitive compounds. Electronic absorption spectra (UV-Vis) were collected on a Perkin Elmer 950 UV-Vis/NIR spectrophotometer.

X-ray Crystallography:

X-ray intensity data were collected on a Brüker APEXII CCD area detector or a Brüker APEXIII D8QUEST CMOS area detector, both employing graphite-monochromated Mo-K α radiation (λ= 0.71073 Å) at 100(1) K. Rotation frames were integrated using SAINT,[2] producing a listing of unaveraged F^2 and $\sigma(F^2)$ values which were then passed to the SHELXT[3] program package for further processing and structure solution. The intensity data were corrected for Lorentz and polarization effects and for absorption using SADABS[4] or TWINABS.[5] Refinement was performed by full-matrix least squares based on F^2 using SHELXL.[6] All of the reflections were used during refinement. Non-hydrogen atoms were refined anisotropically and hydrogen atoms were refined using a riding model.

Synthetic methods:

Synthesis of 1:

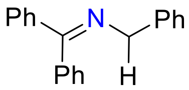

Using a modified literature procedure.[7] In air, add 7.0 g (38.6 mmol) of benzophenone imine and 100 mL of DCM to a 250 mL round bottom flask with a Teflon-coated stirbar. Add 4.14 g (38.6 mmol) of benzyl amine to this solution. Cap the round bottom flask with a needle pierced septum and stir for 14 h. Remove the solvent via rotary evaporation to yield a light yellow oil. Add 25 mL of hexanes to induce precipitation and place in a freezer. Collect the solid via vacuum filtration and dry at 50° C at 50 mtorr overnight. The compound was stored in a N_2 filled glovebox. Yield: 9.85 g (94 %) NMR spectra matched previously reported spectra.[7]

Synthesis of 1′:

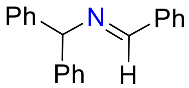

1′ was synthesized according to the literature procedure. NMR spectra matched previously reported spectra.[8]

Synthesis of 3:

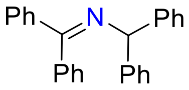

Using a modified literature procedure.[7] In air, add 1.812 g (10 mmol) of benzophenone imine and 1.832 g (10 mmol) of diphenylmethylamine to a 100 mL round bottom containing 50 mL of 1,2-dichloroethane and a Teflon-coated stirbar. Cap the round bottom with a reflux condenser a needle pierced septum and reflux for 48 h. Filter the suspension through a celite plug, collecting the filtrate. Remove the solvent by rotary evaporation, yielding a white crystalline solid. Dry the compound at 50° C at 50 mtorr overnight. Transfer the solid to a medium porosity fritted filter and wash the solid with pentane (3x2 mL) in a N_2 filled glovebox and dry under reduced pressure for 1 h. Yield: 2.56 g (74 %) NMR spectra matched previously reported spectra.[9]

Synthesis of 3′:

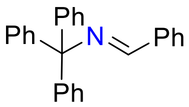

Using a modified literature procedure.[8] To a flame dried flask equipped with a stirbar and 1.000 gram of Na_2SO_4, was added 10 mL of dry DCM, benzaldehyde (0.265 g, 2.5 mmol) and trityl amine (0.648 g, 2.5 mmol). The reaction was stirred overnight, after which time the solution was filtered through a coarse porosity fritted filter and the solvent removed by rotary evaporation. The resulting solid was dried overnight at room temperature, 50 mtorr, yielding 0.712 g (82 %) NMR spectra matched previously reported spectra.[10]

Synthesis of 3-Ad:

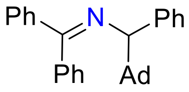

3-Ad was synthesized according to the literature procedure. NMR spectra matched previously reported spectra.[11]

Synthesis of 2-Li:

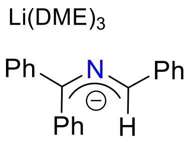

A 20 mL glass scintillation vial was charged with a Teflon-coated stirbar, 4 mL of dimethoxyethane (DME), and 0.500 g (1.84 mmol, 1 equiv) of solid **1**. A solution consisting of 0.175 g of lithium diethyl amide (2.21 mmol, 1.2 equiv) and 4 mL of DME was added dropwise. The vial was sealed and stirred for 18 h. The solution was evacuated to driness, triturated with pentane and redissolved in 4 mL of DME and placed in an -30° C for 1 hour to sufficiently cool, after which it was layered with 4 mL of diethyl ether and 4 mL of pentane and returned to the freezer. After 24 h, the mixture was filtered using a medium porosity fritted filter and the crystalline solid was washed with 3x2 mL of cold diethyl ether and dried under reduced pressure for 3 h. Yield 0.826 g (82%)

Anal. Calcd. for $C_{20}H_{16}NLi{\bullet}2(C_4H_{10}O_2)$ C, 73.50; H, 7.93; N, 3.05. Found: C, 73.19; H, 8.52; N, 2.61.

^{1}H-NMR (500 MHz, *d*^{8}-THF) δ: 7.24 (d, *J* = 7.5 Hz, 4 H), 7.17 (bs, 2 H), 6.98 (bs, 2 H), 6.87 (bs, 1 H), 6.83 (s, 1 H), 6.70 (t, *J* = 7.7 Hz, 4 H), 6.14 (bs, 1 H), 6.11 (t, *J* = 7.0 Hz, 1H), 3.43 (s, 12 H, DME), 3.27 (s, 18 H, DME).

^{13}C{^{1}H}-NMR (126 MHz, *d*^{8}-THF) δ: 147.25, 132.12, 128.45, 128.11, 127.70, 120.58, 120.20, 115.19, 114.93, 107.80, 69.88 (DME), 56.08 (DME).

X-ray quality crystals were obtained from layering concentrated solutions of **2-Li** in cold DME and layering with diethyl ether in a -30° C freezer (1:1 v/v).

Synthesis of 2-Na:

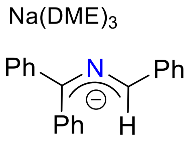

A 20 mL glass scintillation vial was charged with a Teflon-coated stirbar, 10 mL of dimethoxyethane (DME), and 0.173 g of sodium amide (4.422 mmol, 1.2 equiv). To this stirred slurry 1.00 g (3.69 mmol, 1 equiv) of solid **1** was added portionwise. The vial was sealed and stirred for 24 h. Filter the suspension through a Celite packed coarse porosity fritted filter. The filtrate was evacuated to driness and redissolved in 8 mL of DME and placed in an -30° C for 1 hour to sufficiently cool, after which it was layered with 10 mL of pentane and returned to the freezer. After 24 h, the mixture was filtered using a medium

porosity fritted filter and the crystalline solid was washed with 3x2 mL of cold diethyl ether and dried under reduced pressure for 3 h. Yield: 1.57 g (75%)

Anal. Calcd. for $C_{28}H_{20}NNa\cdot3(C_4H_{10}O_2)$ C, 68.06; H, 8.39; N, 2.48. Found: C, 68.79; H, 7.12; N, 2.22. (This represents the best measurement out of three trials)
^{1}H-NMR (500 MHz, *d*^{8}-THF) δ: 7.26 (bs, 2 H), 7.19 (bs, 4 H), 6.96 (bs, 3 H), 6.82 (t, *J* = 7.7 Hz, 4 H), 6.73 (s, 1 H), 6.29 (bs, 1 H) 6.25 (t, *J* = 7.1 Hz, 1H), 3.43 (s, 12 H, DME), 3.27 (s, 18 H, DME).
^{13}C{^{1}H}-NMR (126 MHz, *d*^{8}-THF) δ: 143.13, 139.38, 129.41, 126.14, 126.07, 121.67, 116.83, 113.57, 113.05, 105.31, 69.88 (DME), 56.08 (DME).
X-ray quality crystals were obtained from layering concentrated solutions of **2-Na** in cold DME and layering with pentane in a -30° C freezer (1:1 v/v).

Synthesis of 2-K:

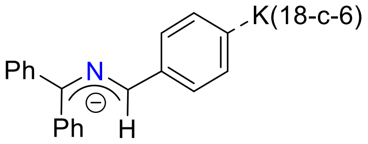

A 20 mL glass scintillation vial was charged with a Teflon-coated stirbar, 10 mL of toluene ,0.500 g (1.84 mmol, 1 equiv) of **1**, and 0.487 g (1.84 mmol, 1 equiv) of 18-crown-6. To this mixture was added 0.217 g (1.93 mmol, 1.05 equiv) of KO*t*Bu as a solid. The now red-purple mixture was stirred for 2 hours at room temperature. Volatiles were removed under reduced pressure. 10 mL of fresh toluene were added and the mixture was then heated to dissolve the purple powder. The resulting solution was allowed to slowly cool to room temperature before being moved into a -30° C freezer. After 24 h, the mixture was filtered using a medium porosity fritted filter and the crystalline solid was washed with 3x2 mL of cold diethyl ether and dried under reduced pressure for 3 h. Yield 0.950 g (90%)

Anal. Calcd. for $C_{28}H_{20}NK\cdot(C_{12}H_{24}O_6)$ C, 66.99; H, 7.03; N, 2.44. Found: C, 66.96; H, 6.66; N, 2.40.

^{1}H-NMR (500 MHz, *d*^{8}-THF) δ: 7.27 (t, *J* = 7.6 Hz, 4 H), 7.19 (bs, 3 H), 6.89 (bs, 2 H), 6.82 (s, 1 H), 6.72 (t, *J* = 7.4 Hz, 4 H), 6.14 (bs, 1 H) 6.11 (t, *J* = 7.0 Hz, 1H).

^{13}C{^{1}H}-NMR (126 MHz, *d*^{8}-THF) δ: 146.38, 131.25, 127.49, 127.09, 126.69, 122.06, 119.62, 119.16, 114.11, 113.71, 106. 62, 70.06 (18-c-6). X-ray quality crystals were obtained from recrystallization from hot toluene.

Synthesis of 4:

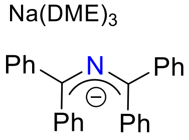

A 20 mL glass scintillation vial was charged with a Teflon-coated stirbar, 10 mL of DME, and 0.136 g of sodium amide (3.45 mmol, 1.2 equiv). To this stirred slurry 1 g (2.88 mmol, 1 equiv) of solid **3** was added portionwise. The vial was sealed

and stirred for 24 h. Filter the suspension through a Celite packed coarse porosity fritted filter. The filtrate was evacuated to driness and redissolved in 8 mL of DME and placed in an -30° C for 1 hour to sufficiently cool, after which it was layered with 10 mL of pentane and returned to the freezer. After 24 h, the mixture was filtered using a medium porosity fritted filter and the crystalline solid was washed with 3x2 mL of cold diethyl ether and dried under reduced pressure for 3 h. Yield: 1.45 g (78%)

Anal. Calcd. for $C_{26}H_{20}NNa \cdot 3(C_4H_{10}O_2)$ C, 71.22; H, 8.02; N, 2.19. Found: C, 71.25; H, 7.93; N, 2.11.

^{1}H-NMR (500 MHz, $CDCl_3$) δ: 6.72 (bs, 20 H), 3.41 (s, 12 H, DME), 3.25 (s, 18 H, DME). The broadness of the peak centered at 6.72 does not change with increasing temperature.

^{13}C{^{1}H}-NMR (126 MHz, $CDCl_3$) δ: 69.72 (DME), 56.01 (DME). No signals belonging to the 2-azaallyl portion could be seen at room or elevated temperatures.

X-ray quality crystals were obtained from layering concentrated solutions of # in cold DME and layering with pentane in a -30° C freezer (1:1 v/v).

Dimerization of 2-Na yielding $5_{1\text{-}1}$-rac $5_{1\text{-}1}$-meso, and $5_{1\text{-}3}$:

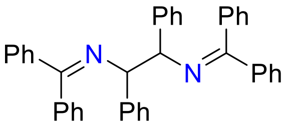

$5_{1\text{-}1}$-meso; $5_{1\text{-}1}$-rac

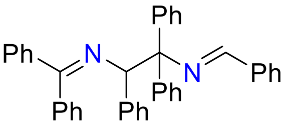

$5_{1\text{-}3}$

A 20 mL glass scintillation vial was charged with a Teflon coated stirbar, 4 mL of DME and 0.112 g of **2-Na** (0.2 mmol, 1 equiv.) resulting in a purple solution. Another 20 mL glass scintillation vial was charged with 4 mL of DME and 0.040 g of $AgPF_6$ (1.56 mmol, 1 equiv.). Both vials were placed in a -25 °C freezer to cool. After 15 minutes the vials were removed and the Ag^+ solution was slowly added to the solution of azaallyl anion, resulting in a color change upon complete addition to a yellow solution with black particulate. The solution was filtered using a Celite packed coarse porosity fritted filter. Remove volatiles under vacuum. The solid was redissolved in dichloromethane and filtered again through an alumina packed coarse porosity fritted filter, washing with dichloromethane. Again the volatiles were removed under reduced pressure. The primary species present in the solid were confirmed to be **$5_{1\text{-}1}$-rac** and **$5_{1\text{-}1}$-meso** in a 2:3 ratio by H^1 NMR in $CDCl_3$.

Synthesis of 6:

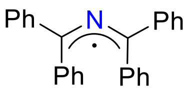

A 20 mL glass scintillation vial was charged with a Teflon coated stirbar, 4 mL of DME and 0.100 g of **4-Na** (1.56 mmol, 1 equiv.) resulting in a purple solution. Another 20 mL glass

scintillation vial was charged with 4 mL of DME and 0.040 g of $AgPF_6$ (1.56 mmol, 1 equiv.). Both vials were placed in a -25 °C freezer to cool. After 15 minutes the vials were removed and the Ag^+ solution was slowly added to the solution of azaallyl anion, resulting in a color change upon complete addition to a green solution with black particulate. The solution was filtered using a Celite packed coarse porosity fritted filter. Remove volatiles under reduced pressure. The solid was redissolved in pentane and filtered again using a Celite packed coarse porosity fritted filter. Again the volatiles were removed under reduced pressure resulting in a green powder yielding 0.041 g (82%) of **6**.

HRMS calc'd for $[C_{26}H_{20}N]^+$ = 346.1596, found 346.1594.

X-ray quality crystals were grown from concentrated solutions of **6** in acetonitrile at –25 °C.

Arylation of 2-M:

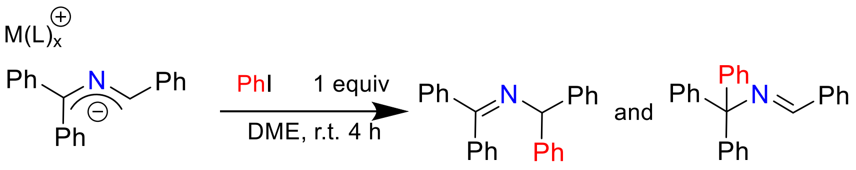

A 4 ml scintillation vial charged with a teflon coated stirbar, was charged with 0.041 g (0.2 mmol) of phenyl iodide and 1 mL of dimethoxyethane. While stirring, 0.4 mmol of **2-M** was added to yield a purple solution. The vial was sealed and stirred for 4 hours. Upon finishing, the vial was exposed to air and 2 drops of water were quickly added, causing a color change from purple to yellow. The solution was passed through 1 mL of silica using 12 mL of EtOAc. The solvent was removed, and to the crude oil mixture was added $CDCl_3$ and 7 μL of dibromomethane were added as an internal standard to determine the yield.

Alkylation of 2-M:

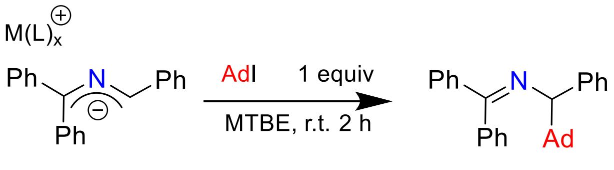

A 4 ml scintillation vial charged with a teflon coated stirbar, was charged with 0.026 g (0.1 mmol) of phenyl iodide and 1 mL of methyl *tert*butyl ether. While stirring, 0.2 mmol of **2-M** was added to yield a purple solution. The vial was sealed and stirred for 2 hours. Upon finishing, the vial was exposed to air and 2 drops of water were quickly added, causing a color change from purple to yellow. The solution was passed through 1 mL of silica using 12 mL of EtOAc. The solvent was removed, and to the crude oil mixture was added $CDCl_3$ and

7 μL of dibromomethane were added as an internal standard to determine the yield.

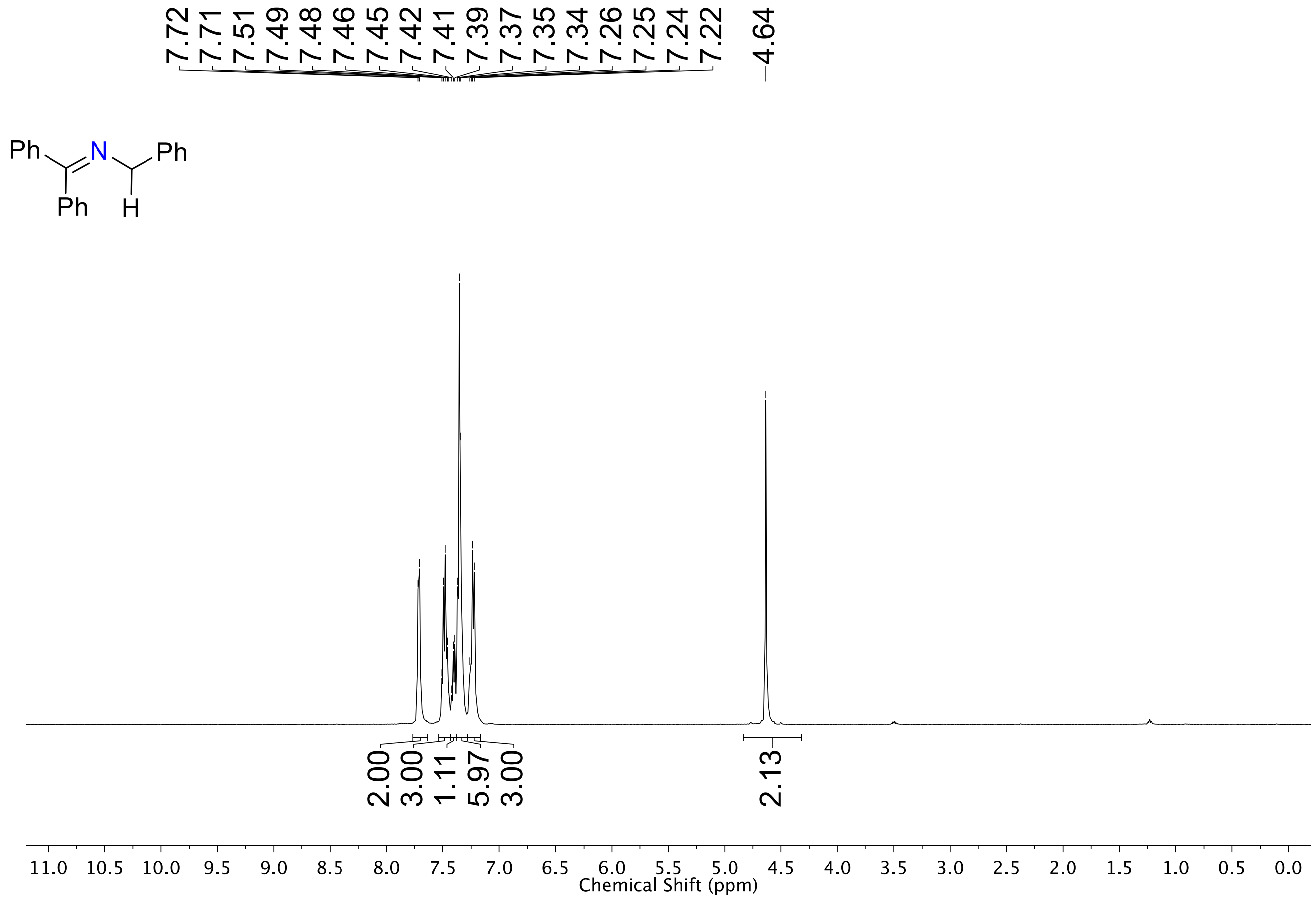

Figure S1a: ^{1}H NMR (500 MHz, $CDCl_3$) spectrum of **1**.

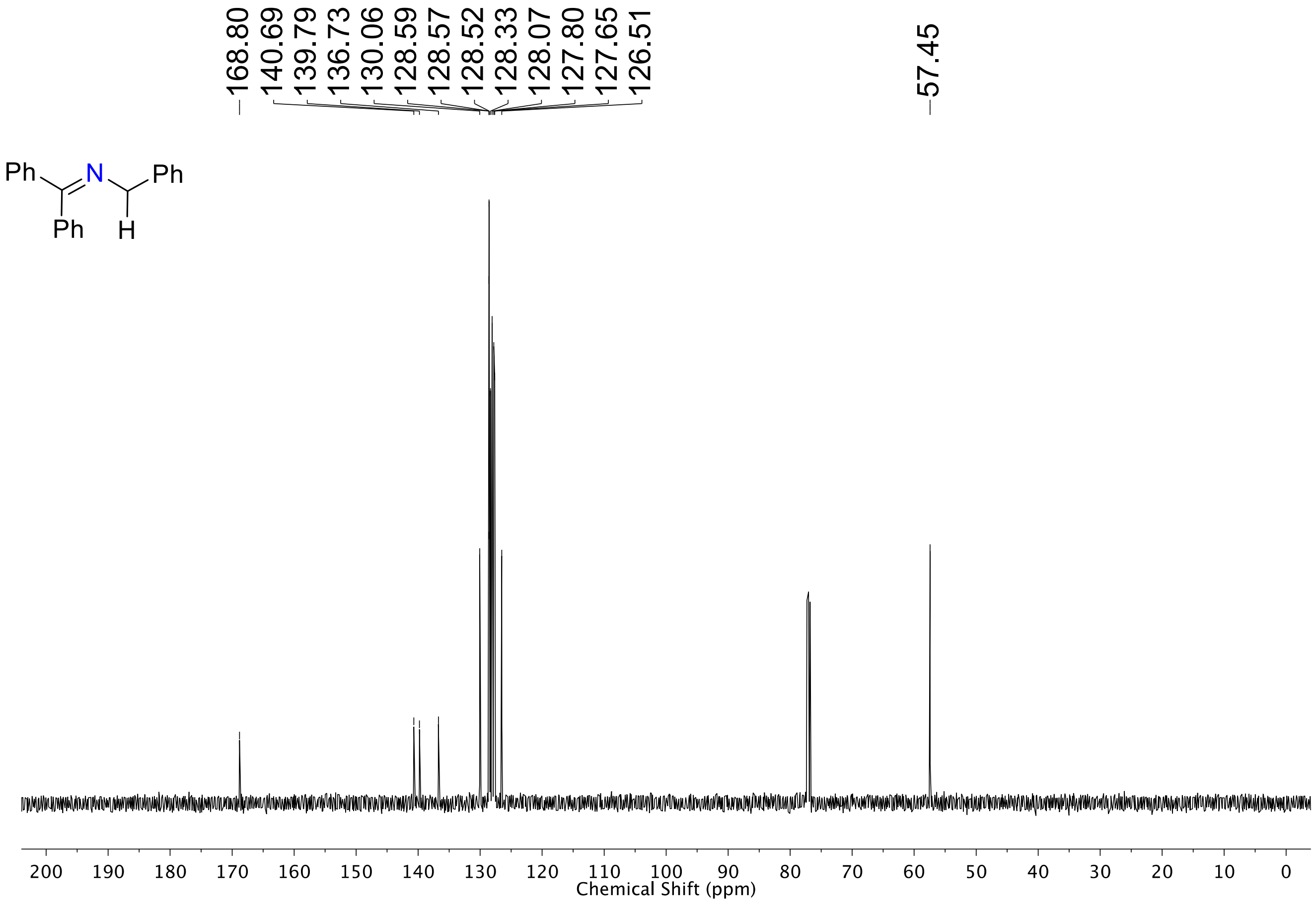

Figure S1b: $^{13}C\{^{1}H\}$ NMR (126 MHz, $CDCl_3$) spectrum of **1**.

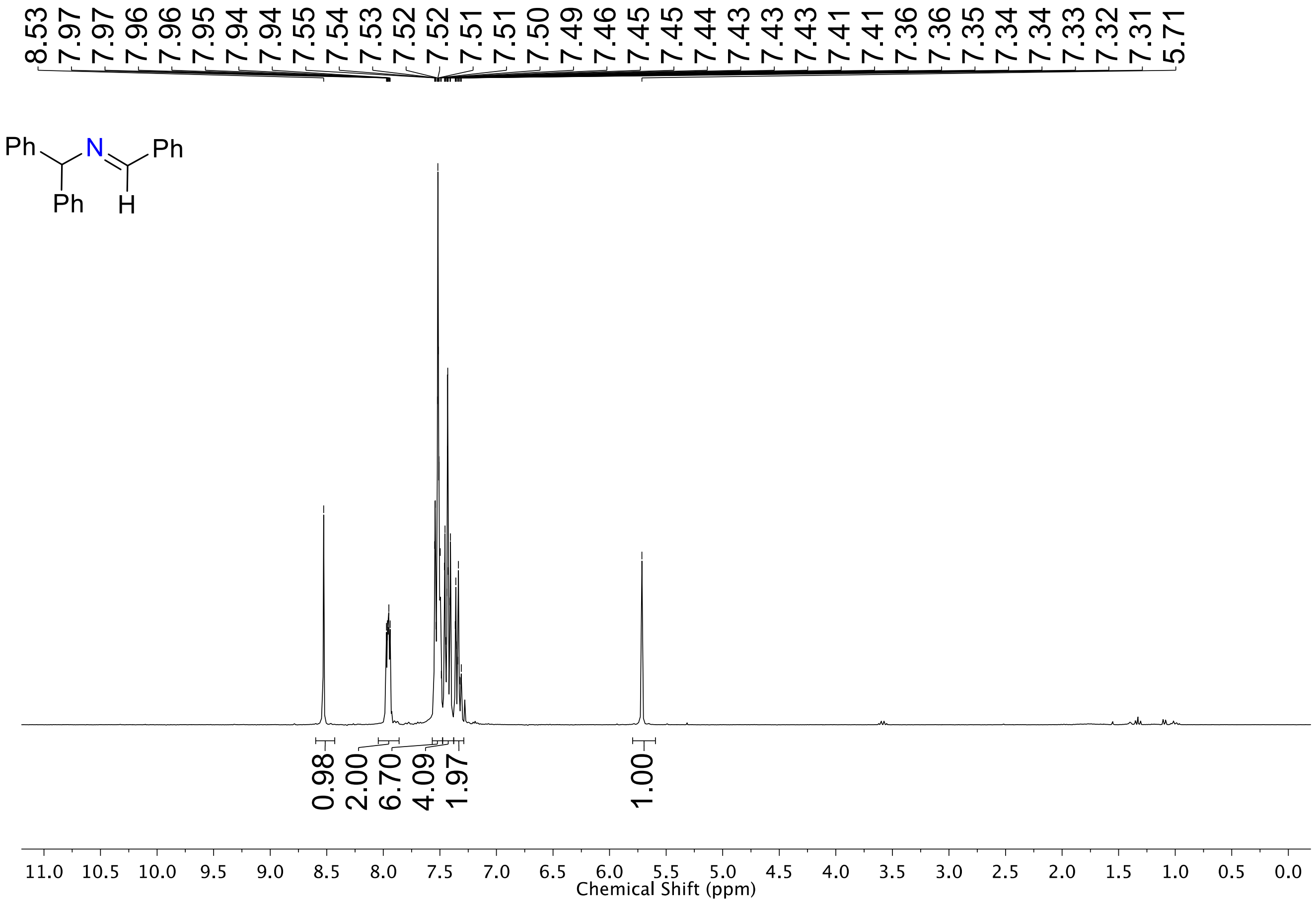

Figure S2a: ^{1}H NMR (300 MHz, $CDCl_3$) spectrum of **1′**.

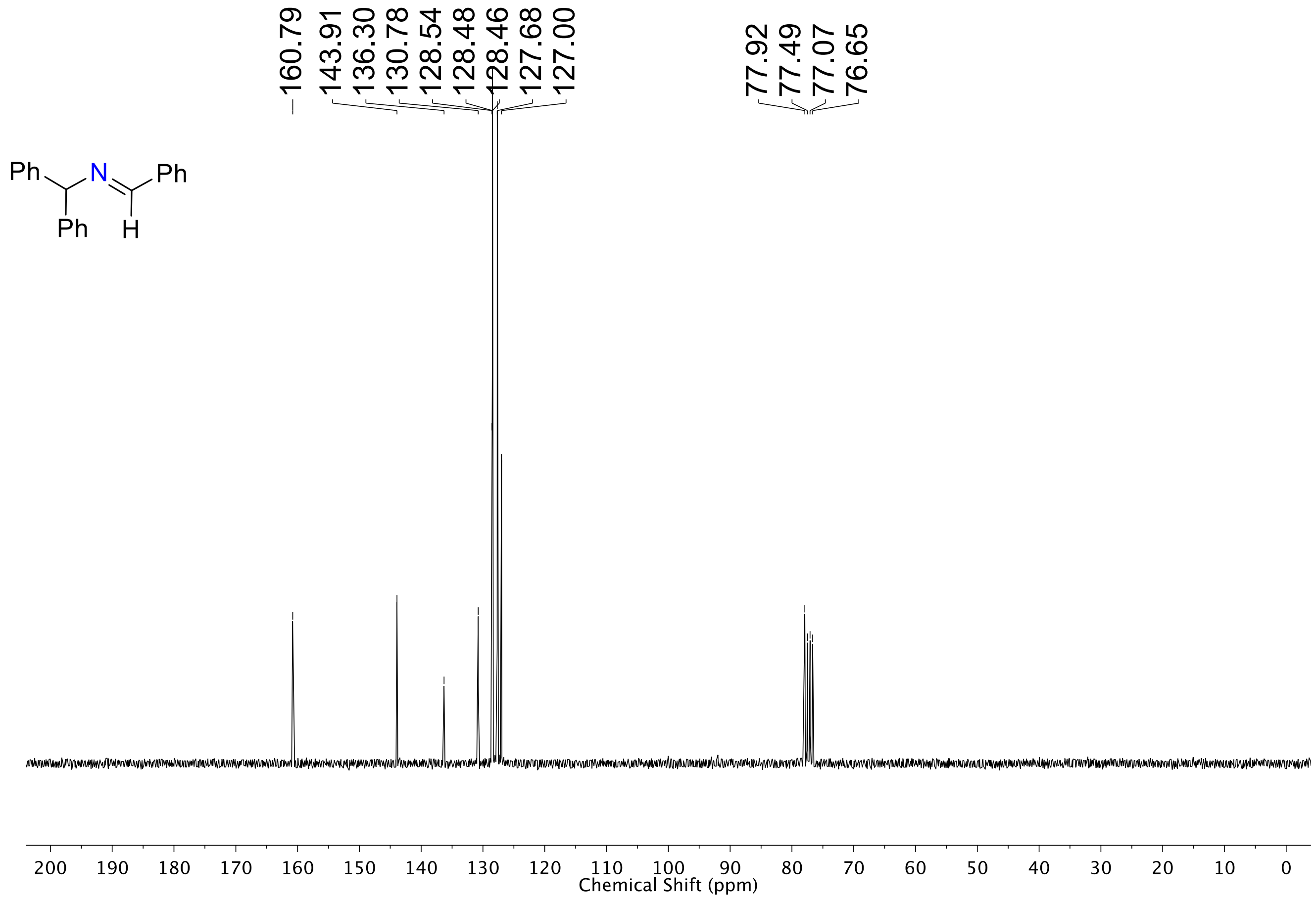

Figure S2b: $^{13}C\{^1H\}$ NMR (126 MHz, $CDCl_3$) spectrum of **1′**.

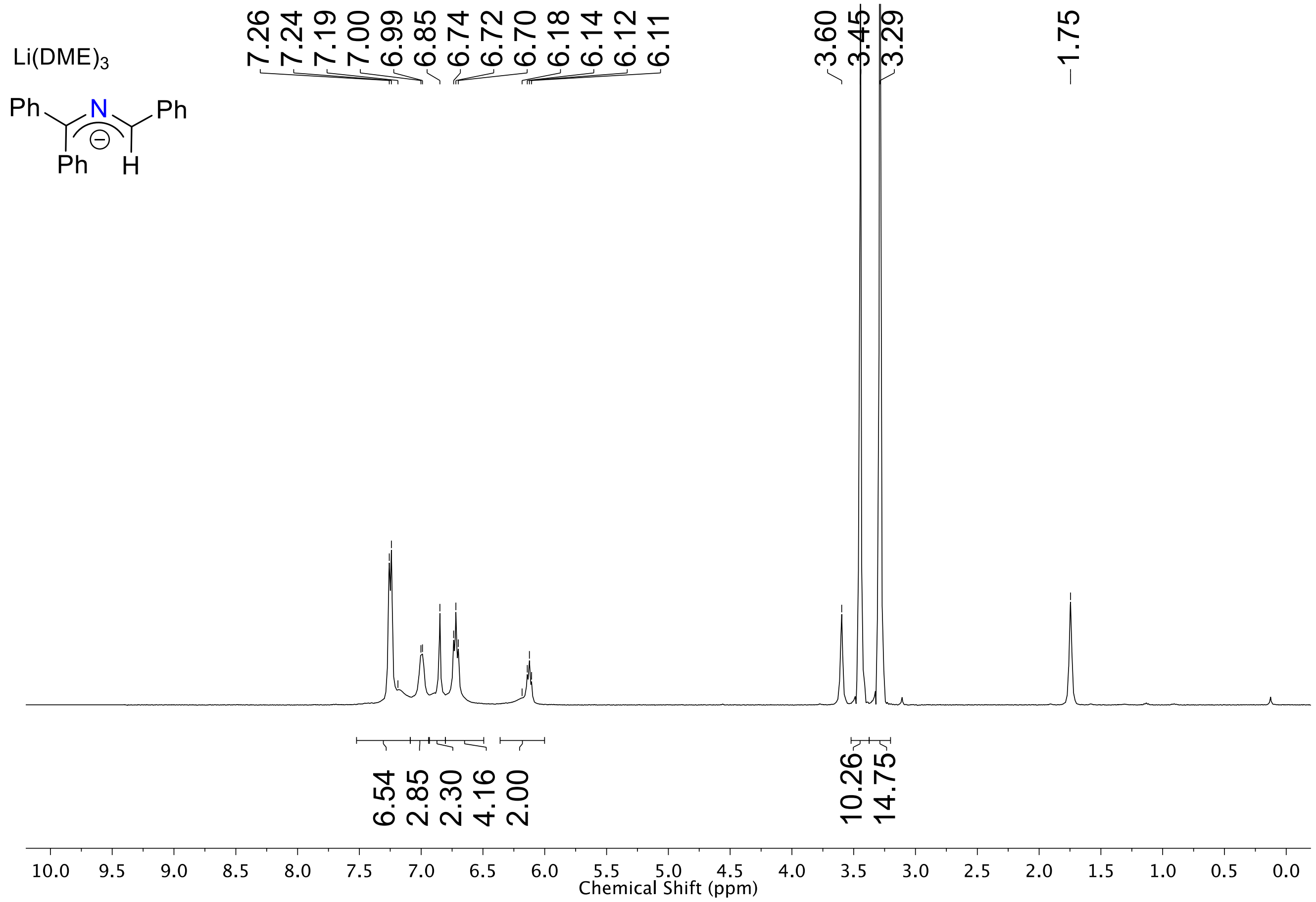

Figure S3a: ^{1}H NMR (400 MHz, THF-d_8) spectrum of **2-Li**.

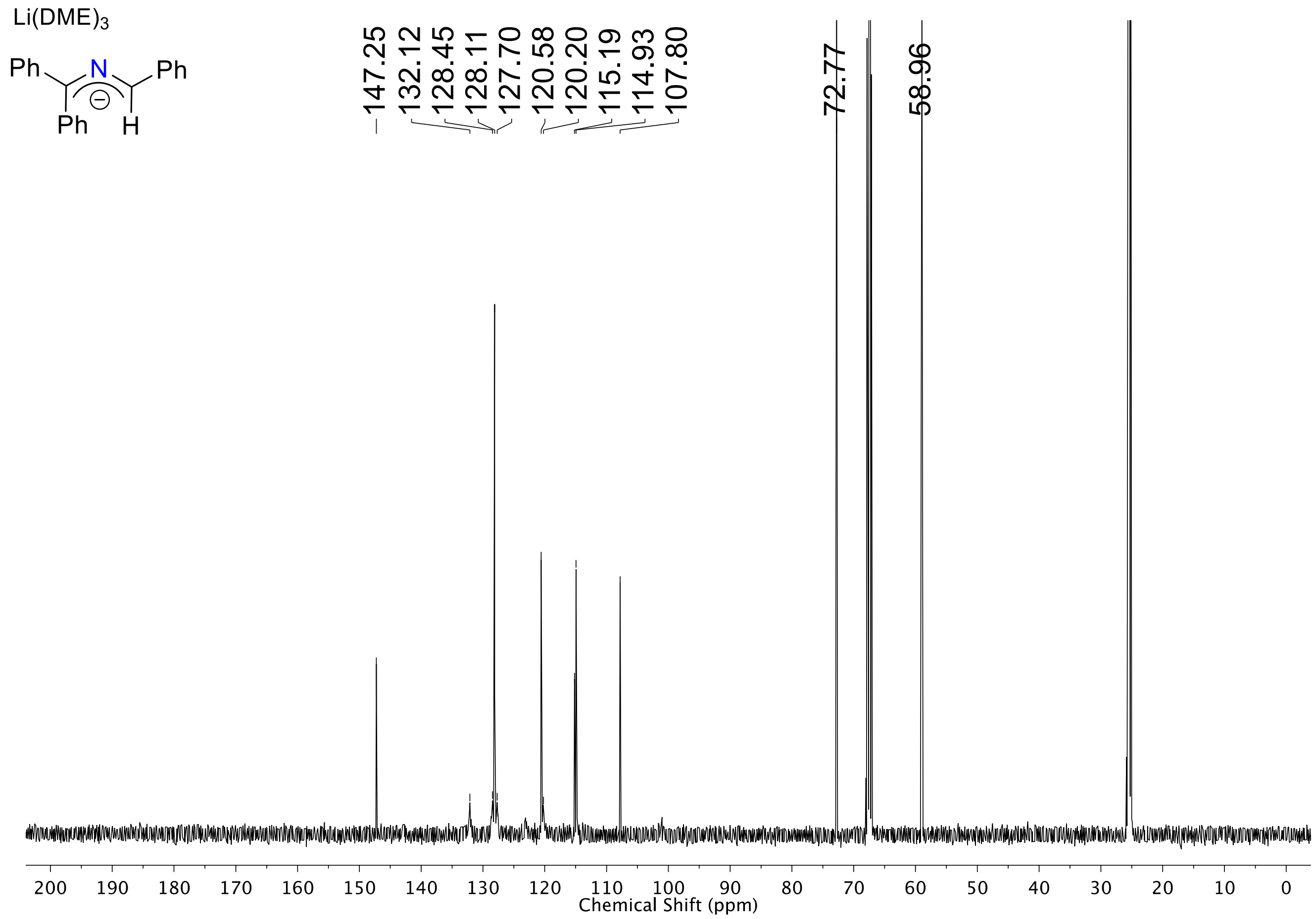

Figure S3b: $^{13}C\{^1H\}$ NMR (126 MHz, THF-d_8) spectrum of **2-Li**.

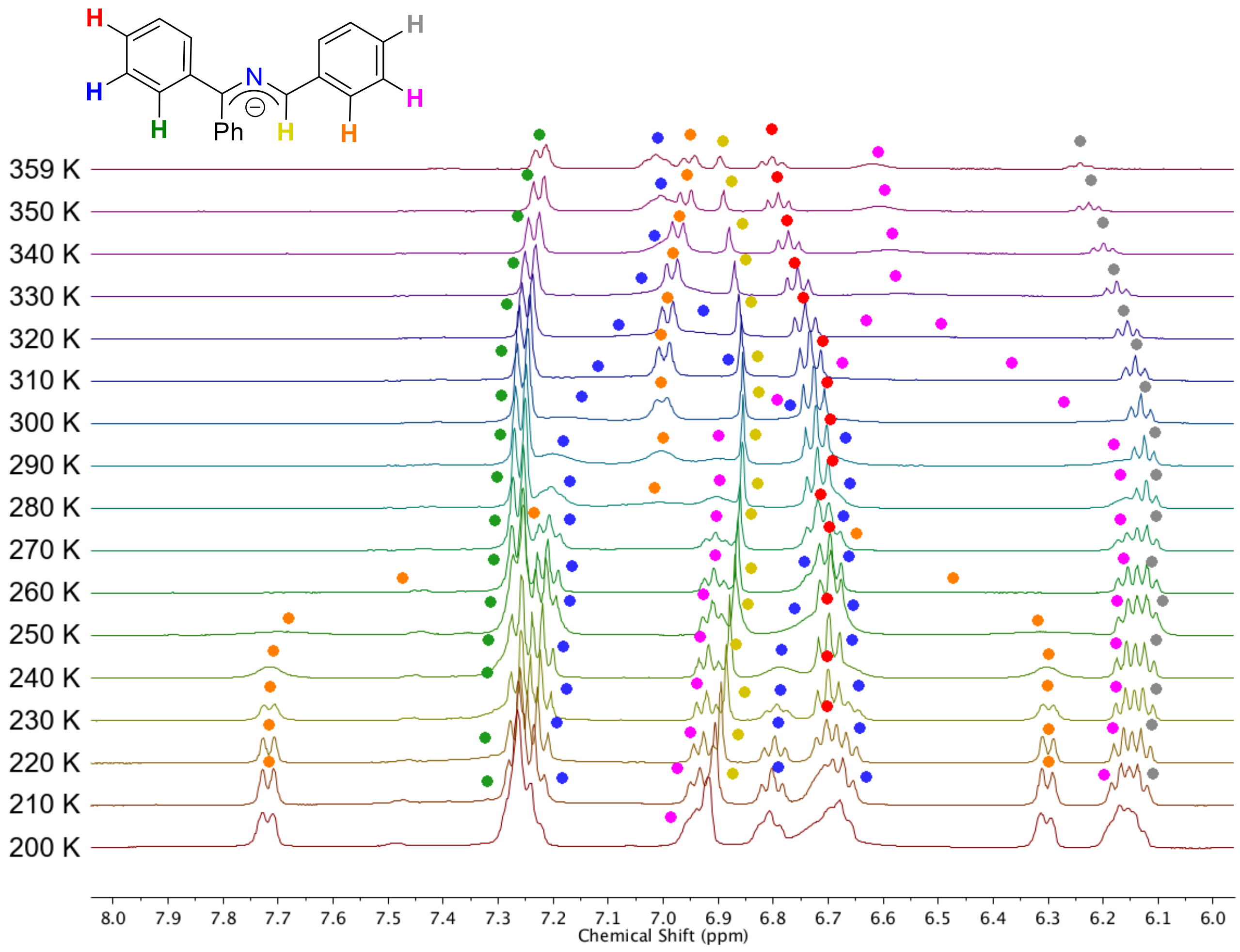

Figure S3c: Variable temperature ^{1}H NMR (400 MHz, THF-d_8) of **2-Li** from 200 - 359 K.

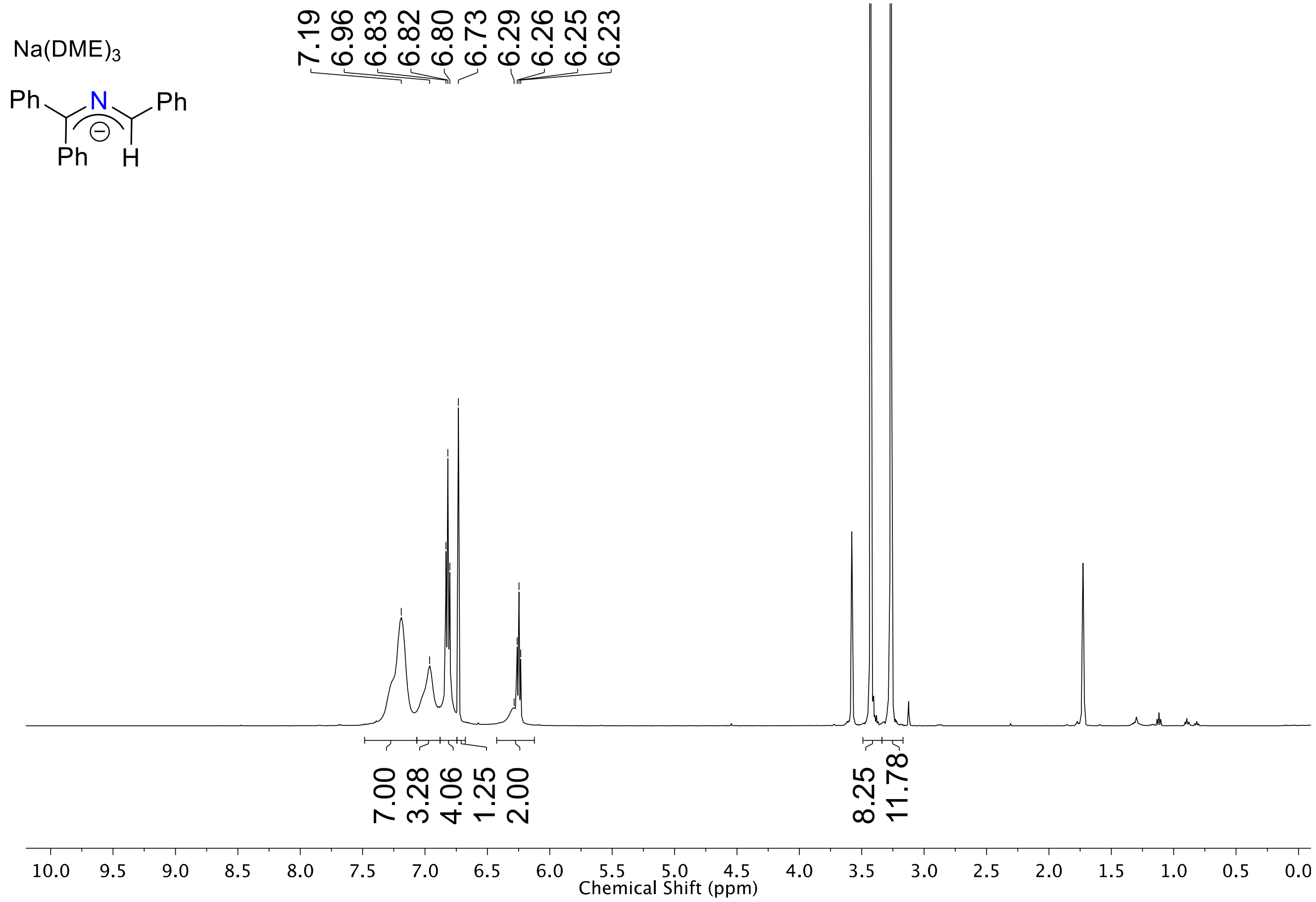

Figure S4a: ^{1}H NMR (500 MHz, THF-d_8) spectrum of **2-Na**.

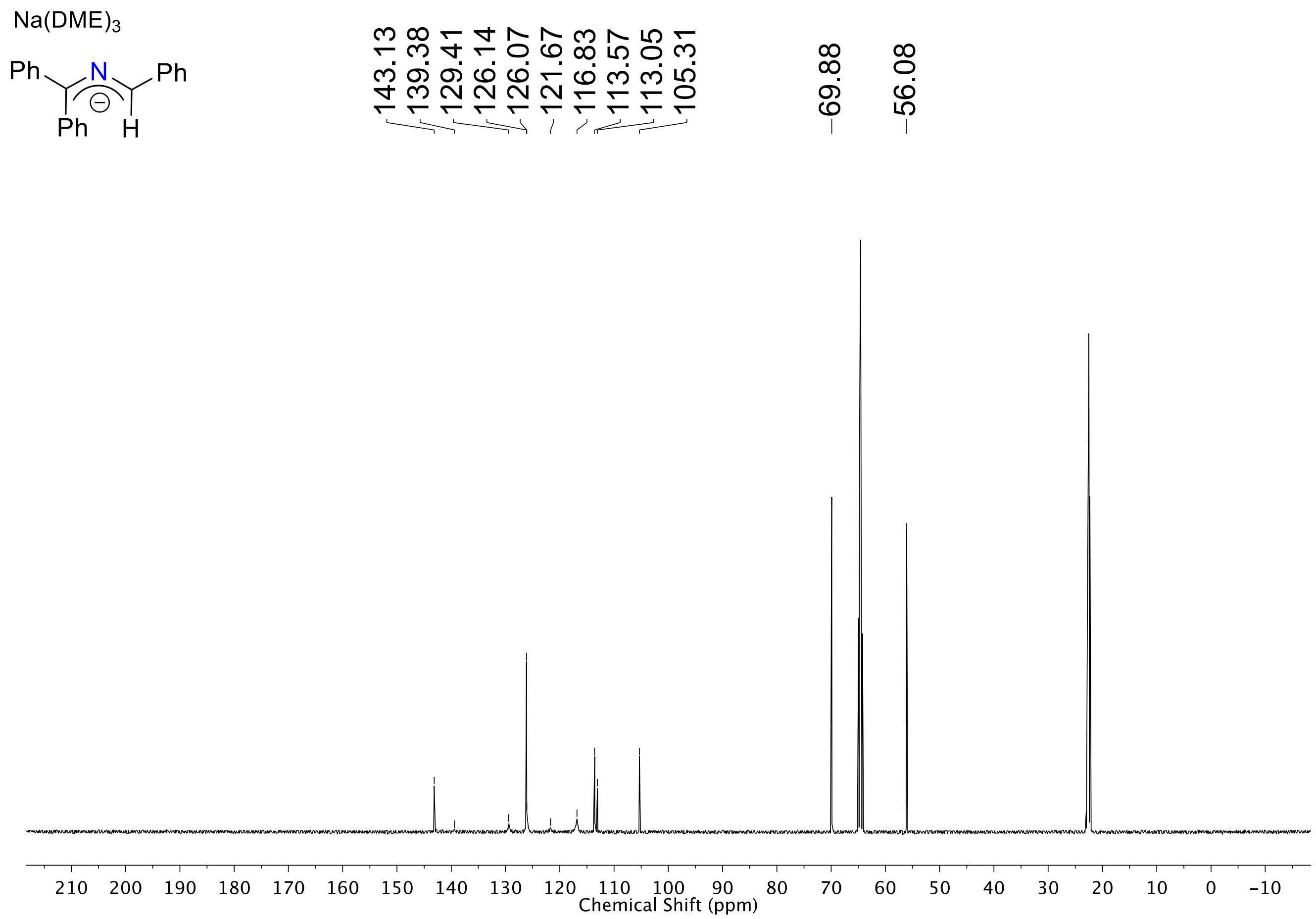

Figure S4b: $^{13}C\{^{1}H\}$ NMR (126 MHz, THF-d_8) spectrum of **2-Na**.

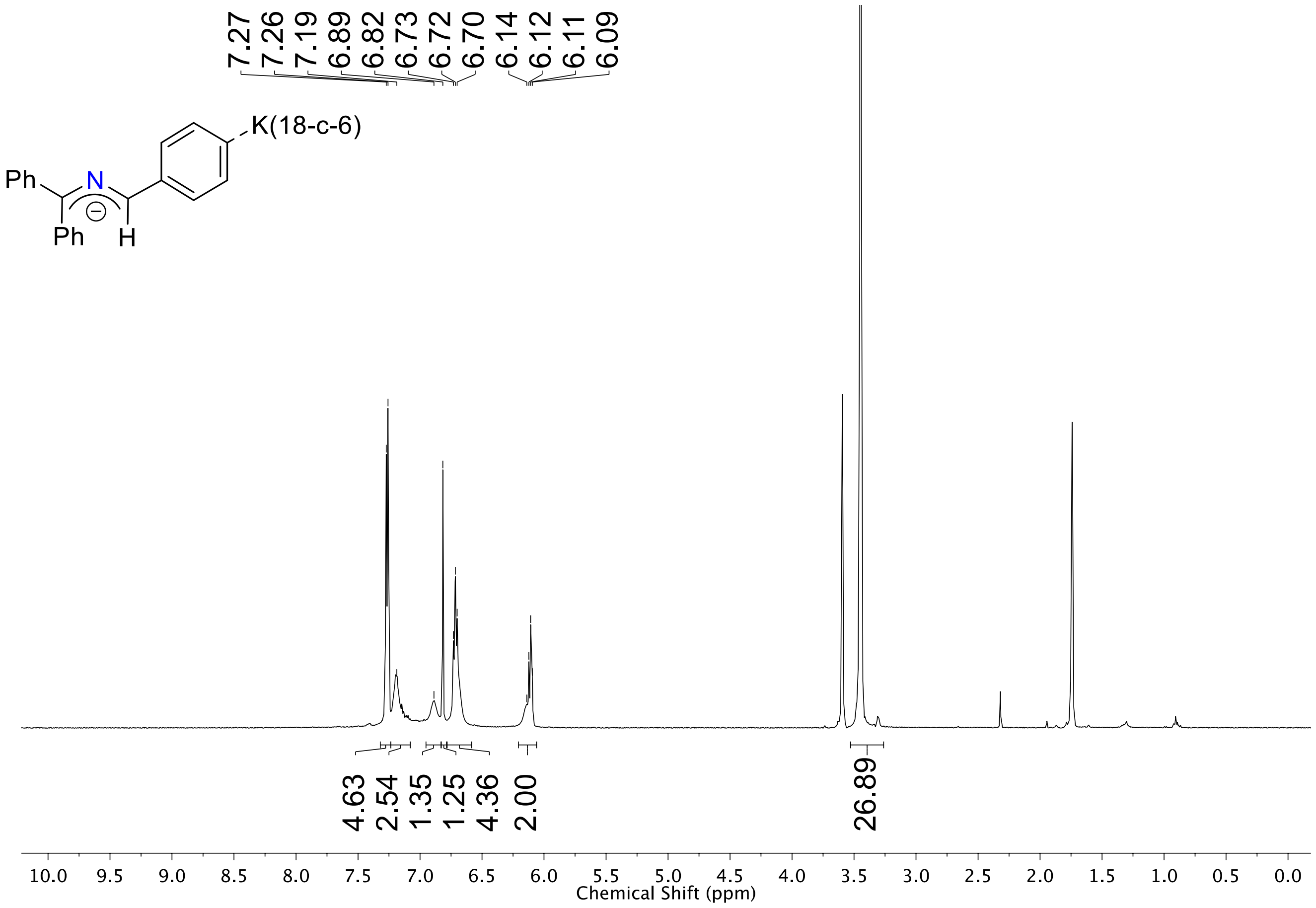

Figure S5a: ^{1}H NMR (500 MHz, THF-d_8) spectrum of **2-K**.

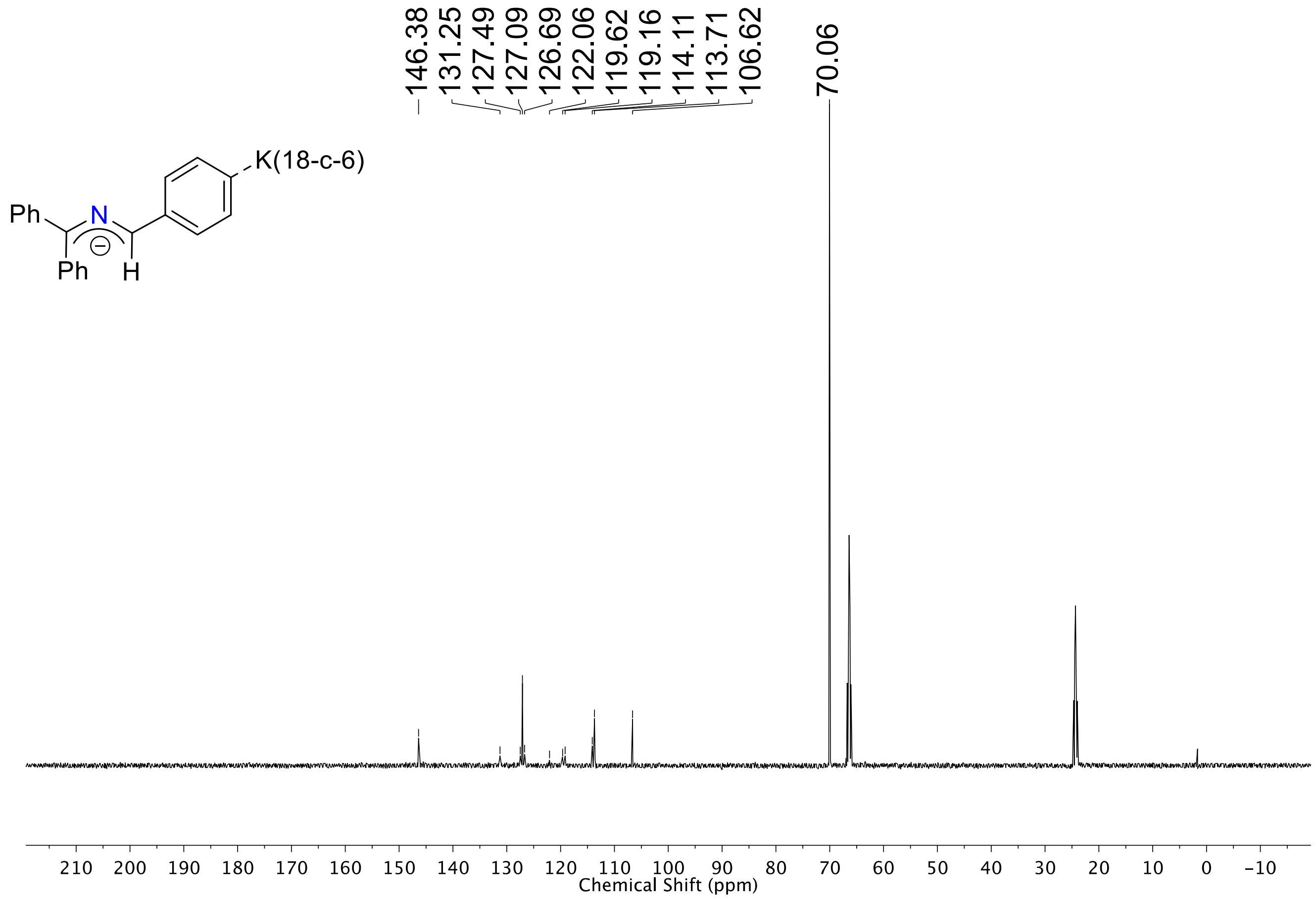

Figure S5b: $^{13}C\{^{1}H\}$ NMR (126 MHz, THF-d_8) spectrum of **2-K**.

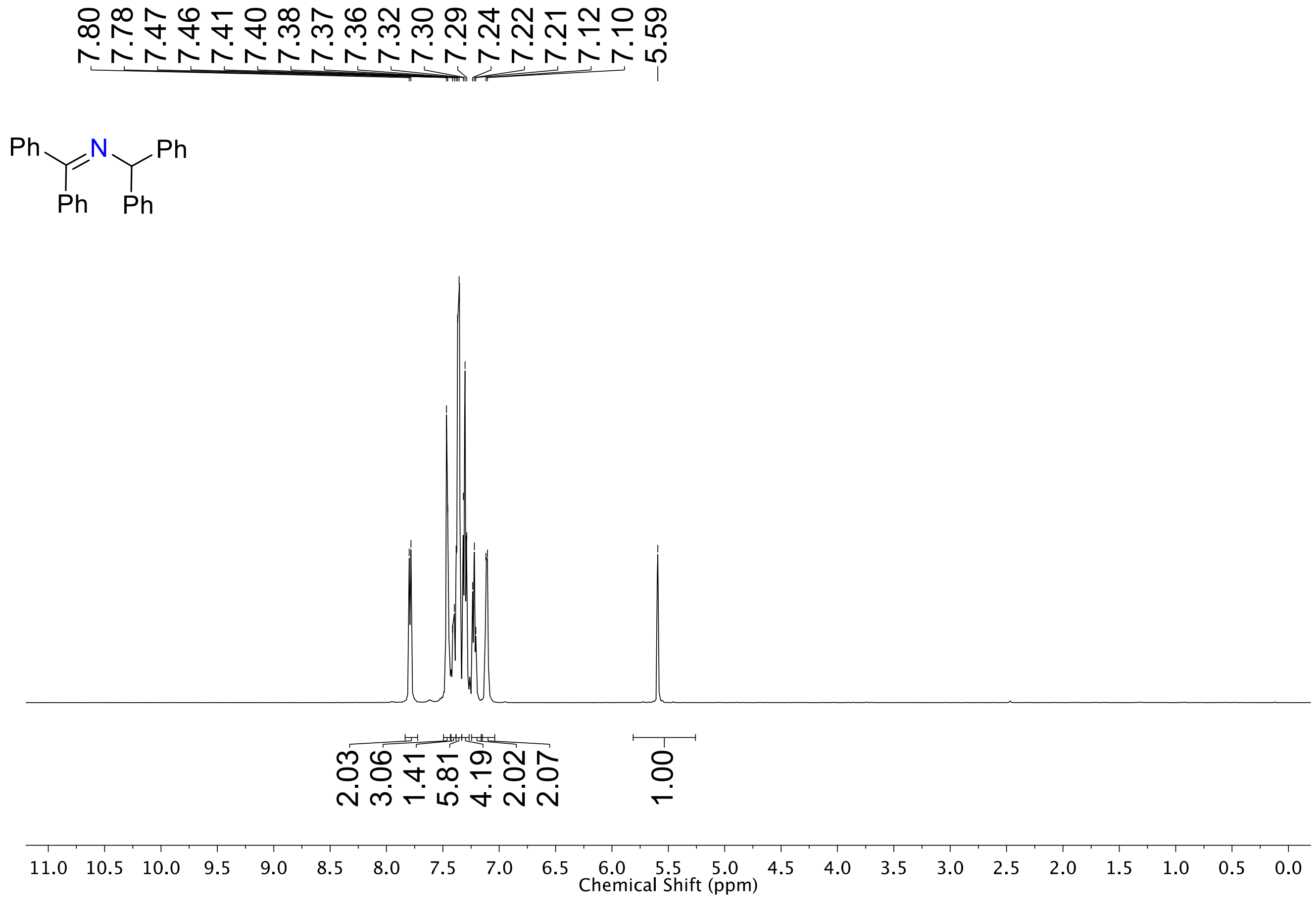

Figure S6a: ^{1}H NMR (500 MHz, $CDCl_3$) spectrum of **3**.

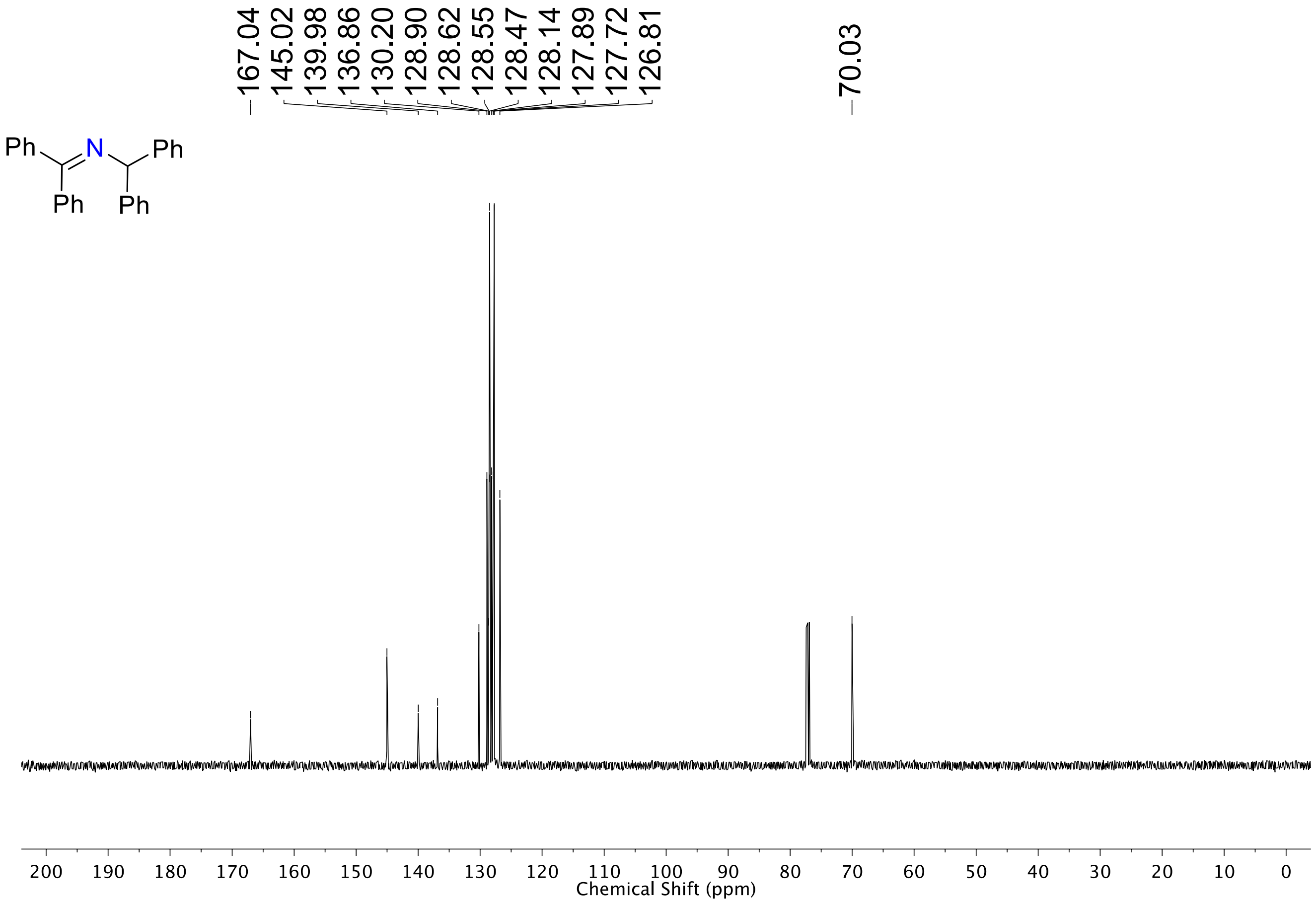

Figure S6b: $^{13}C\{^{1}H\}$ NMR (126 MHz, $CDCl_3$) spectrum of **3**.

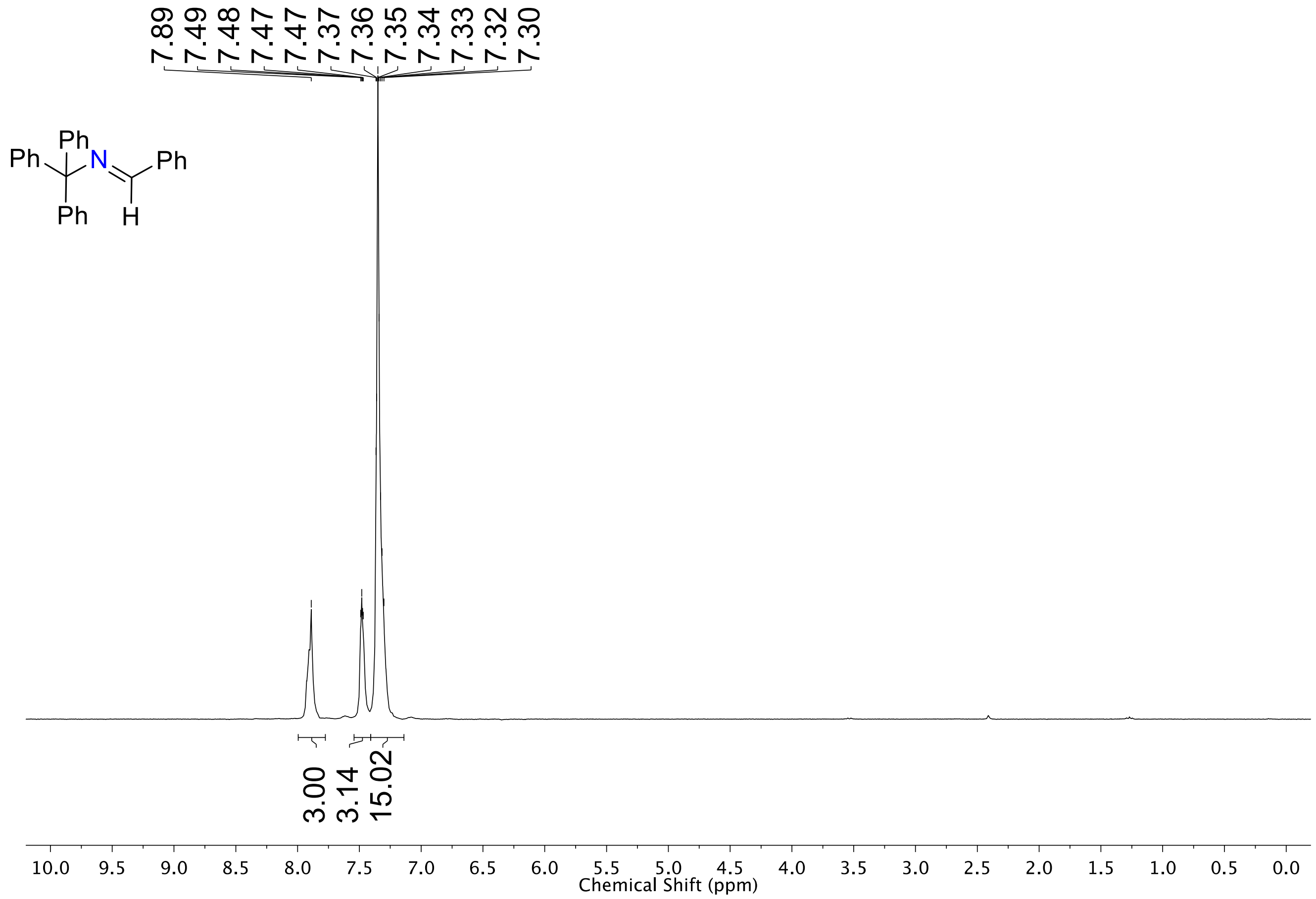

Figure S7a: ^{1}H NMR (300 MHz, $CDCl_3$) spectrum of **3′**.

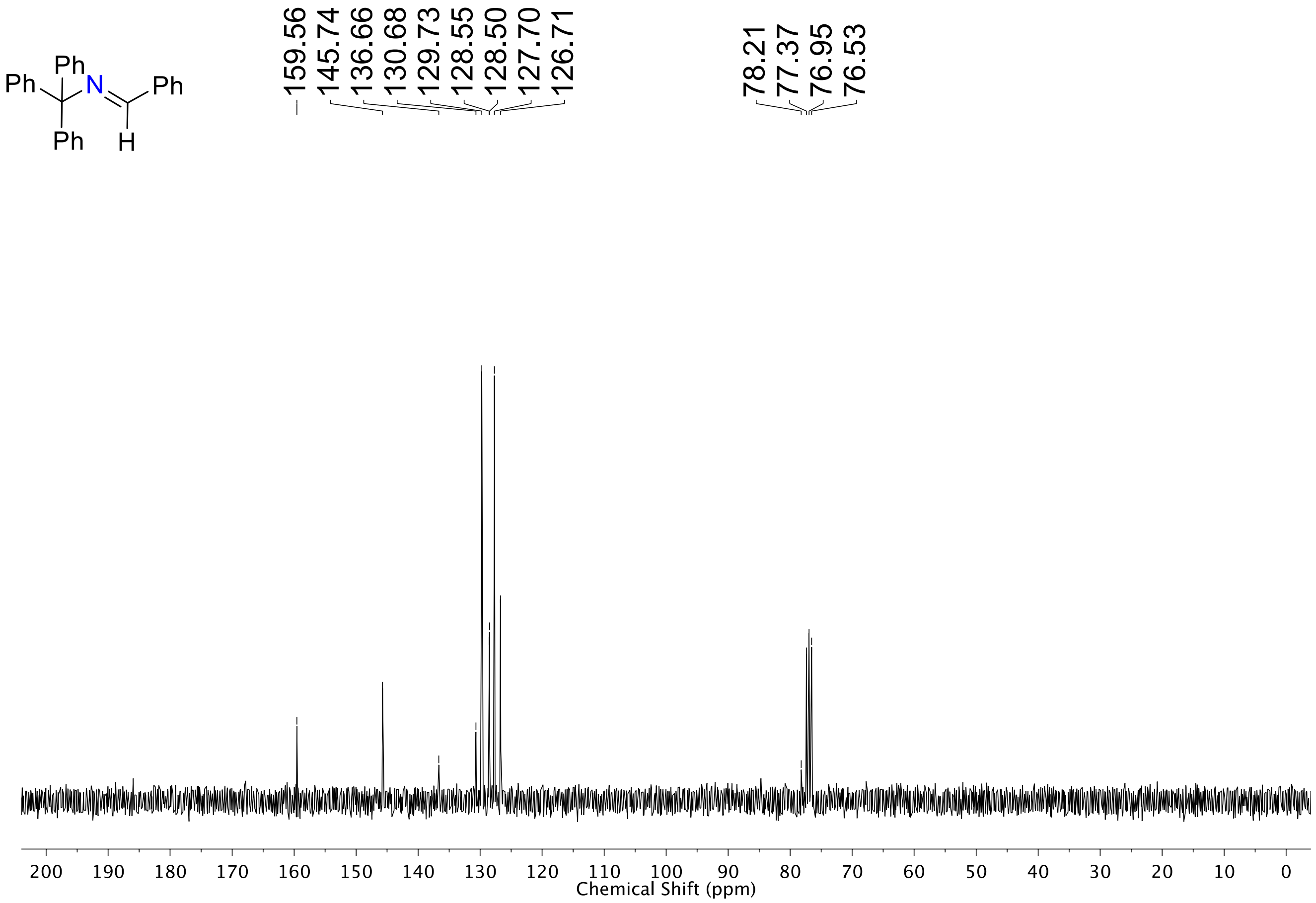

Figure S7b: $^{13}C\{^{1}H\}$ NMR (75 MHz, $CDCl_3$) spectrum of **3′**.

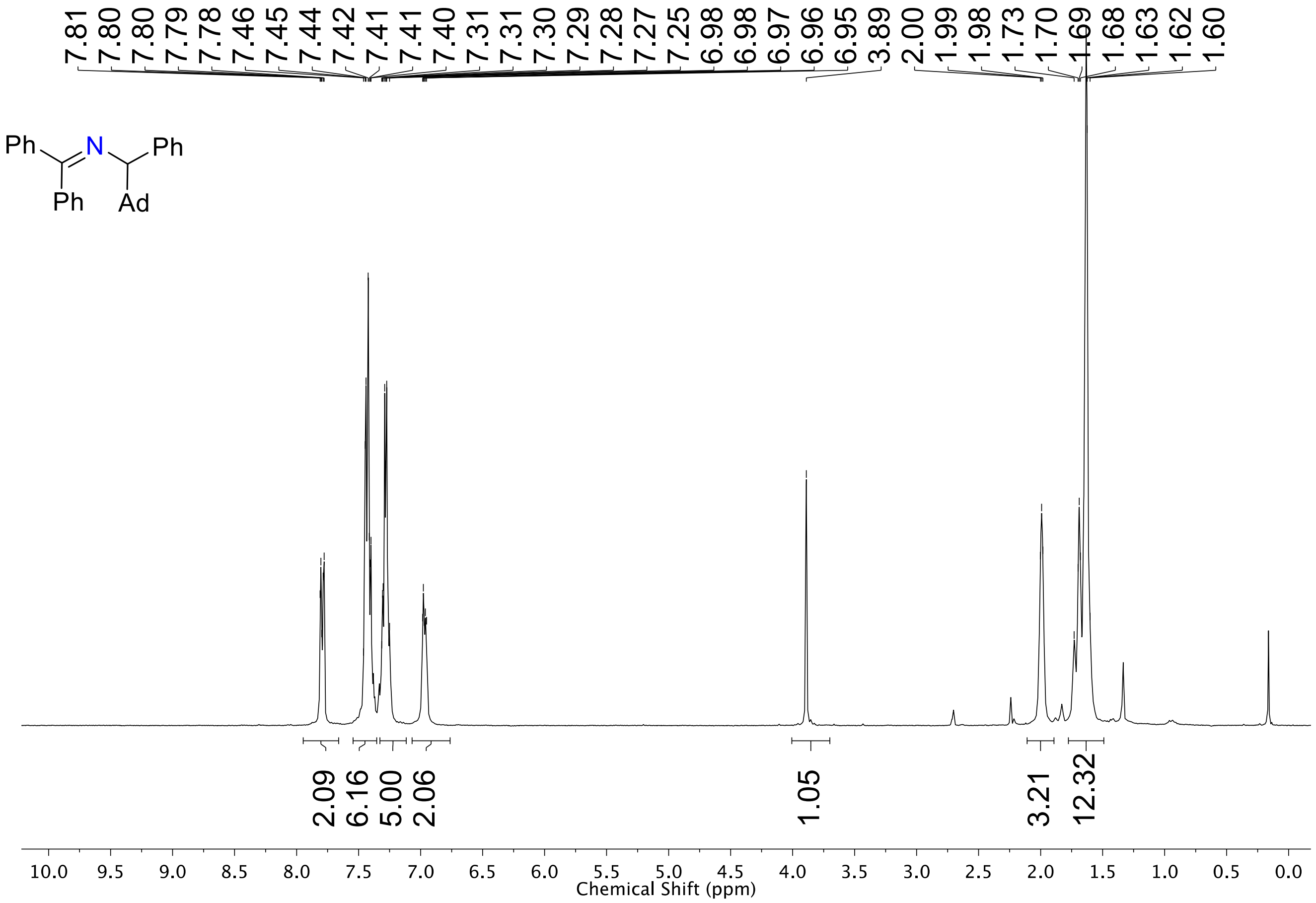

Figure S8a: ^{1}H NMR (300 MHz, $CDCl_3$) spectrum of **3-Ad**.

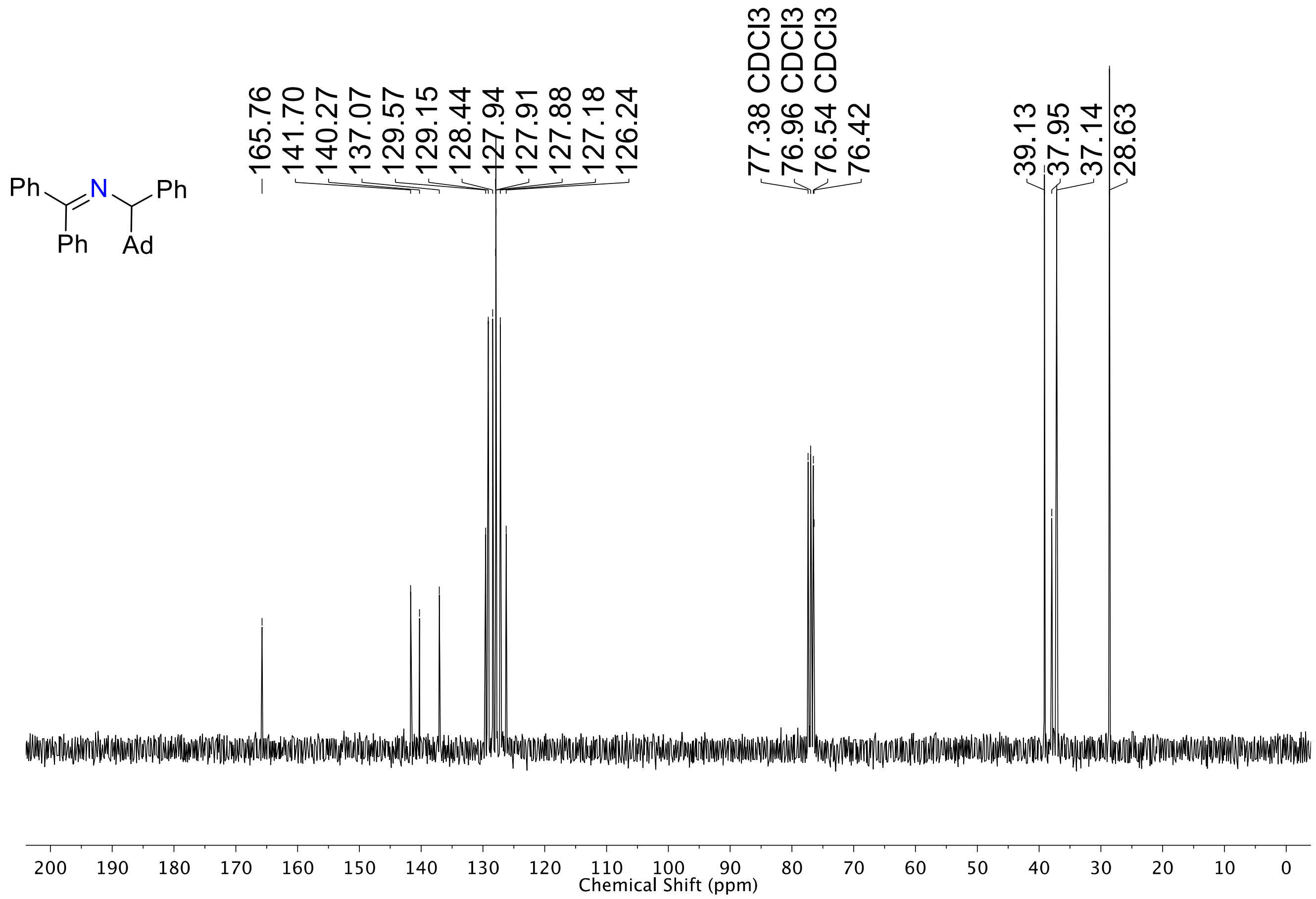

Figure S8b: $^{13}C\{^1H\}$ NMR (75 MHz, $CDCl_3$) spectrum of **3-Ad**.

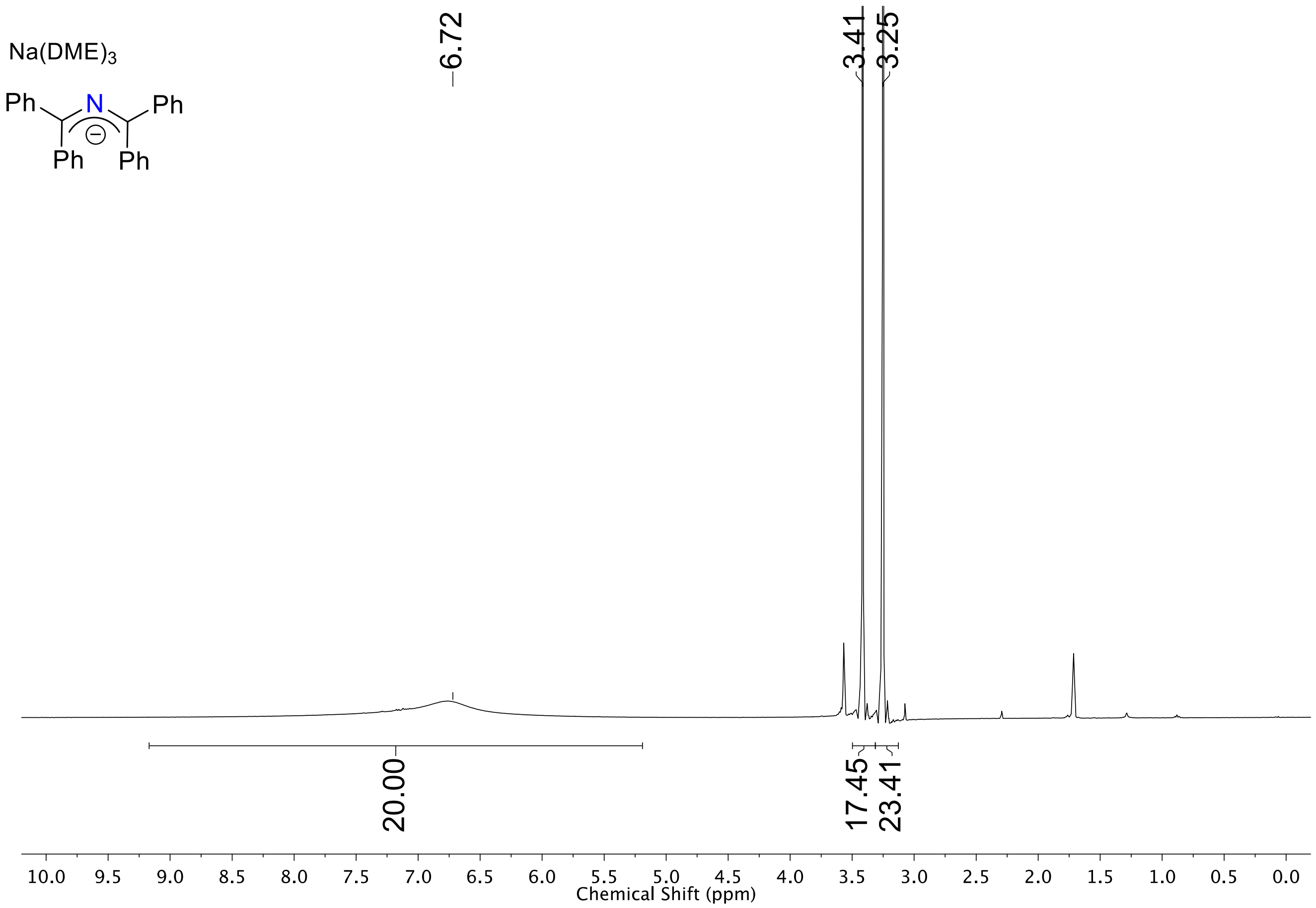

Figure S9a: ^{1}H NMR (400 MHz, THF-d_8) spectrum of **4-Na**.

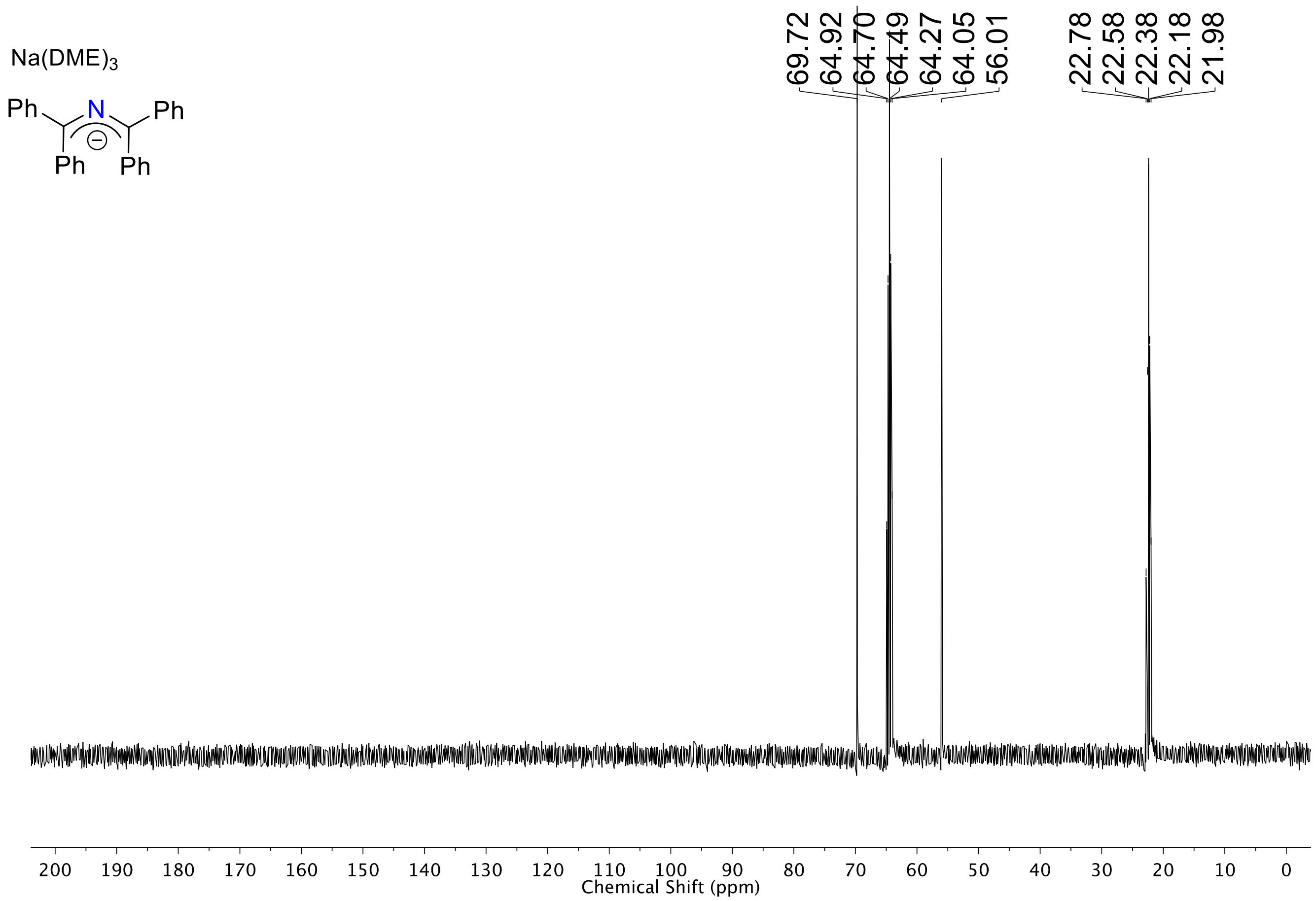

Figure S9b: $^{13}C\{^{1}H\}$ NMR (100 MHz, THF-d_8) spectrum of **4-Na**.

Arylation color coding:

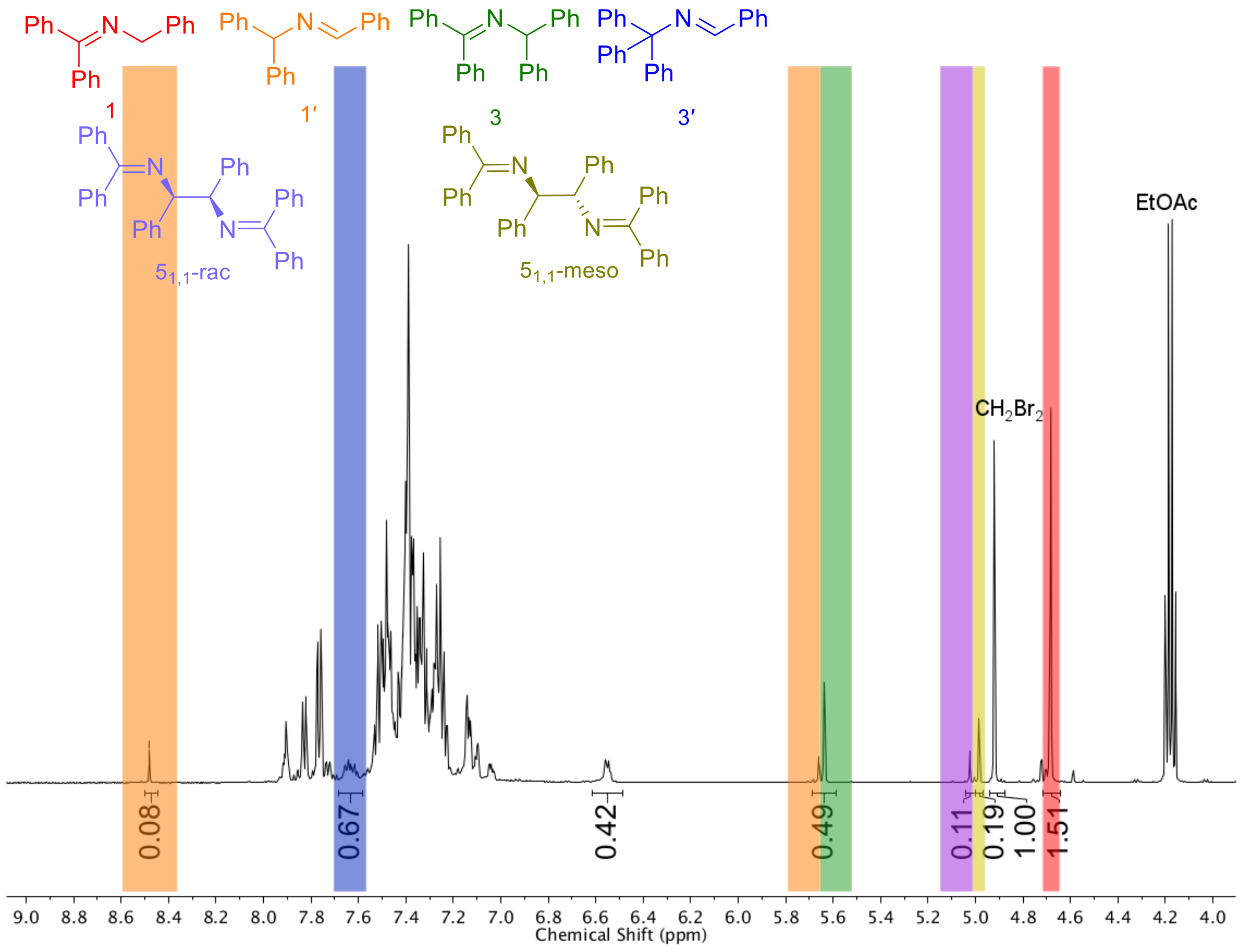

Figure S10: ^{1}H NMR (500 MHz, $CDCl_3$) spectrum of reaction of **2-Li** with PhI

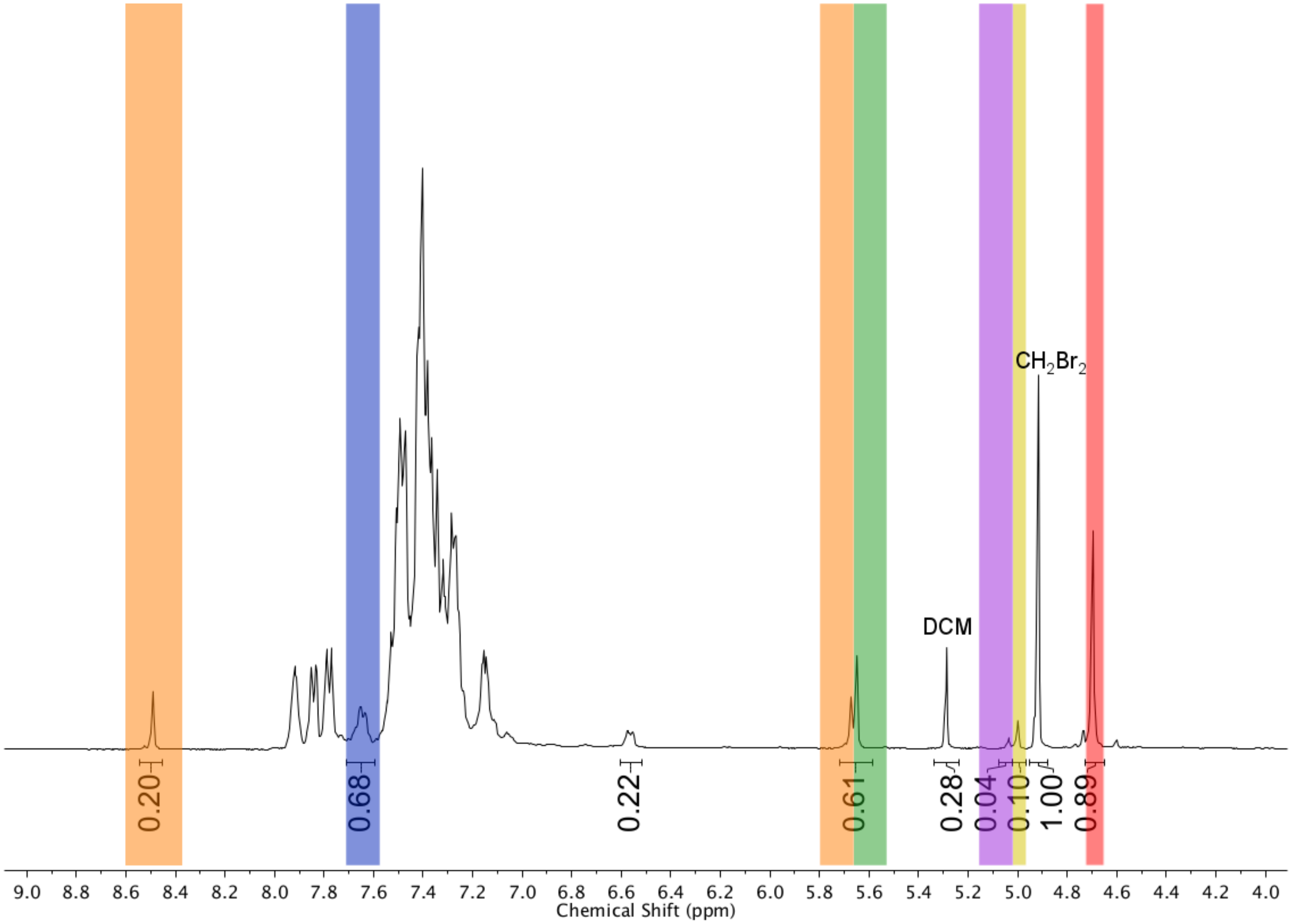

Figure S11: ^{1}H NMR (500 MHz, $CDCl_3$) spectrum of reaction of **2-Na** with PhI.

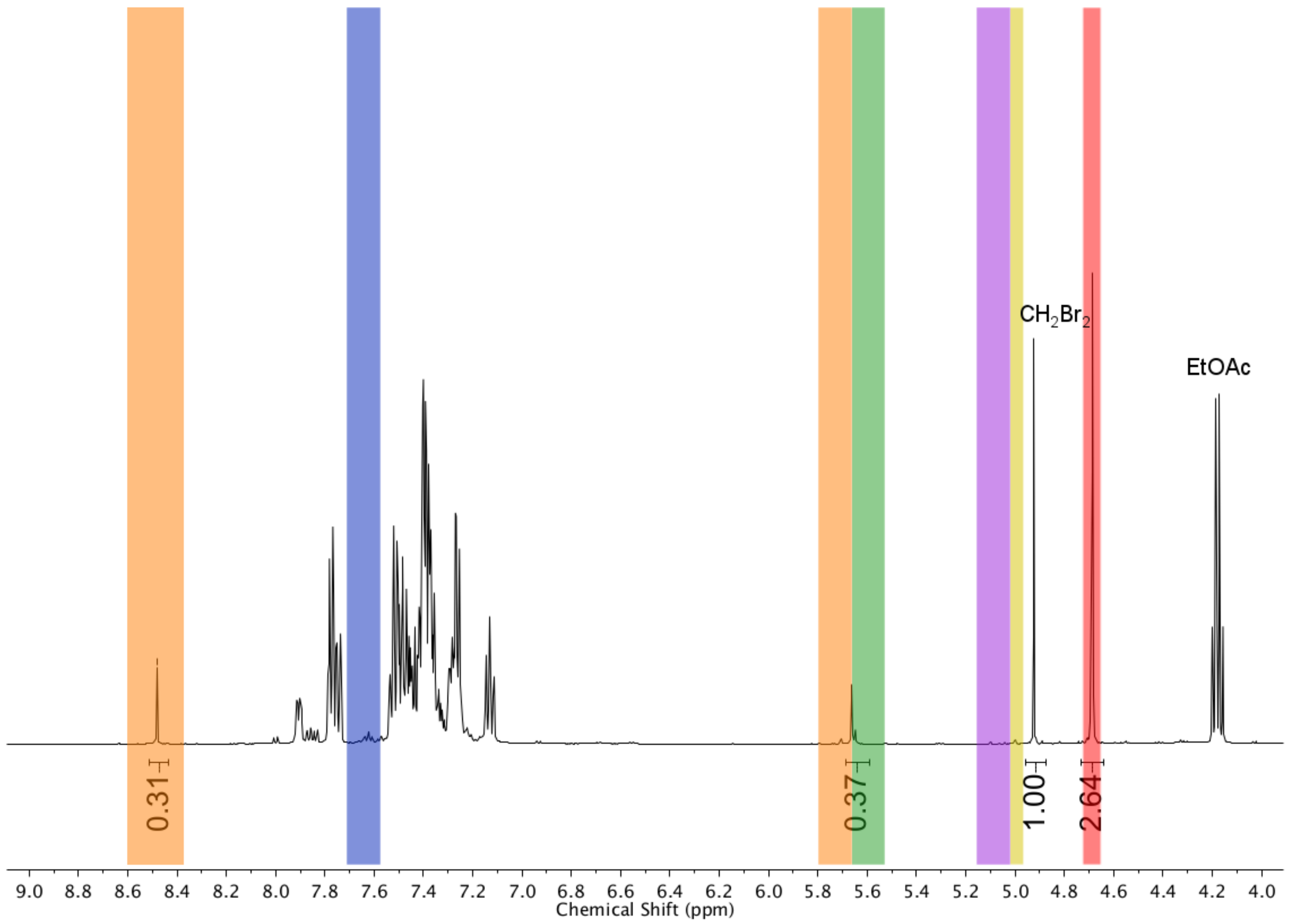

Figure S12: ^{1}H NMR (500 MHz, $CDCl_3$) spectrum of reaction of **2-K** with PhI.

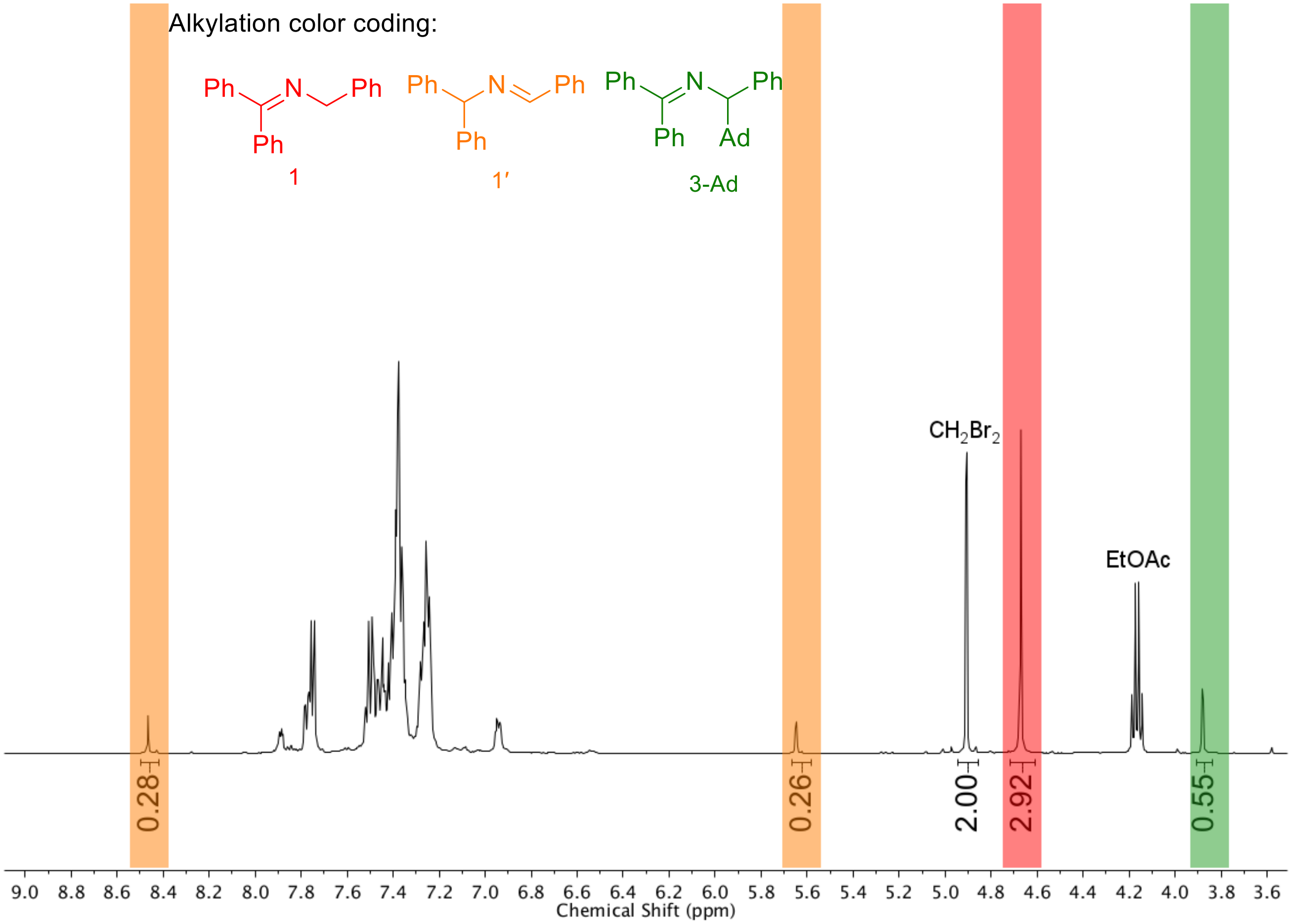

Figure S13: ^{1}H NMR (500 MHz, $CDCl_3$) spectrum of reaction of **2-Li** with AdI.

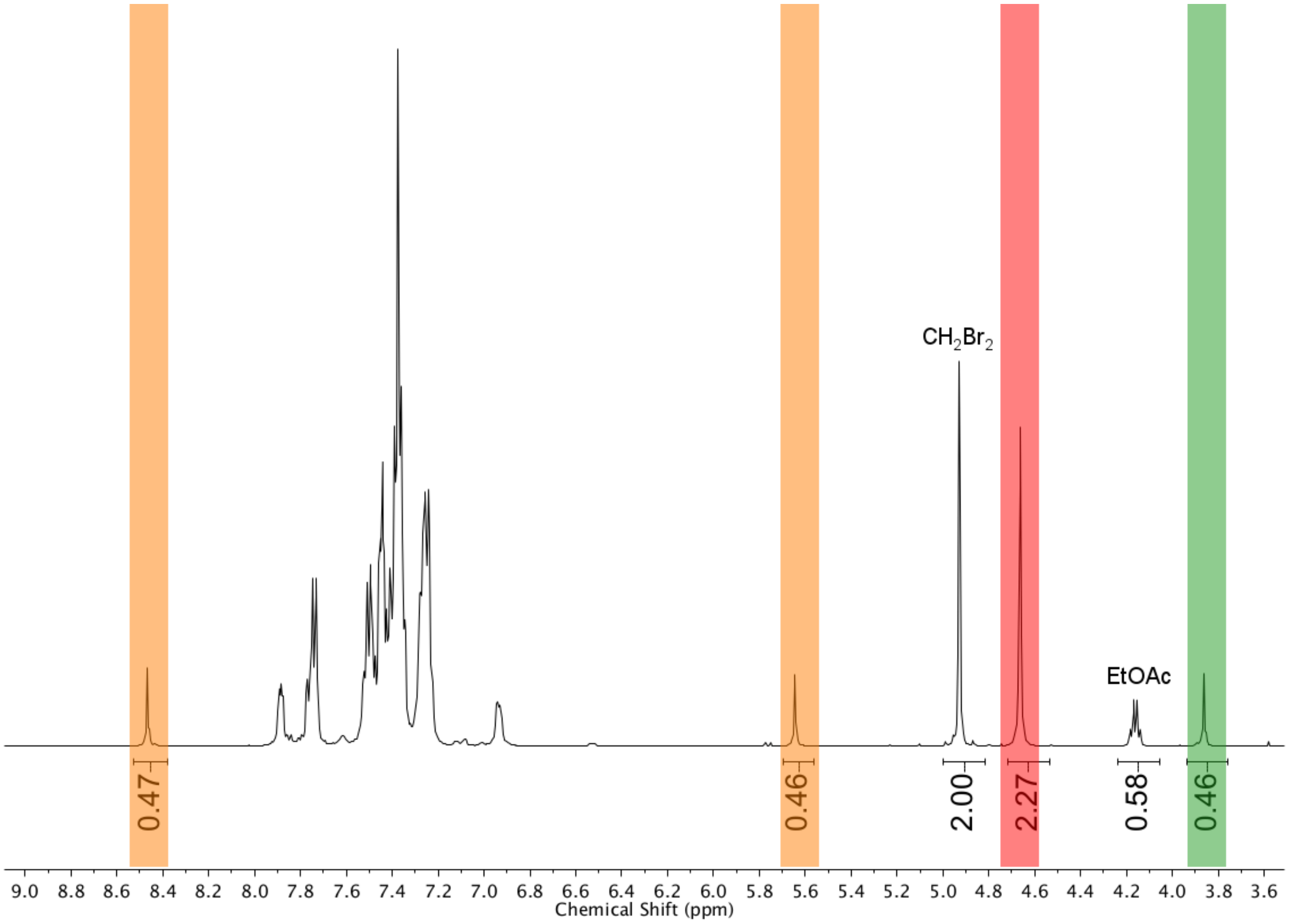

Figure S14: ^{1}H NMR (500 MHz, $CDCl_3$) spectrum of reaction of **2-Na** with AdI.

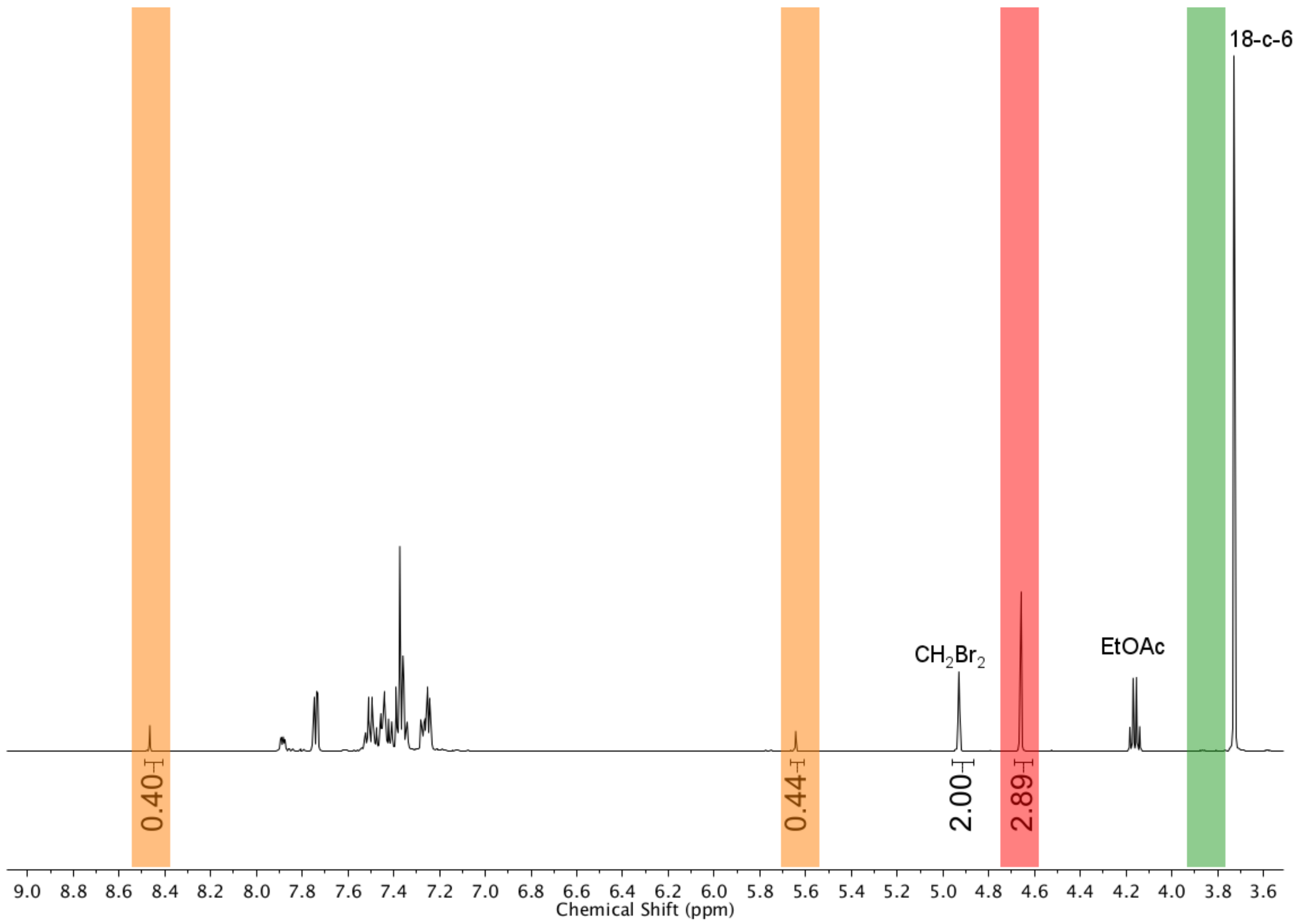

Figure S15: ^{1}H NMR (500 MHz, $CDCl_3$) spectrum of reaction of **2-K** with AdI.

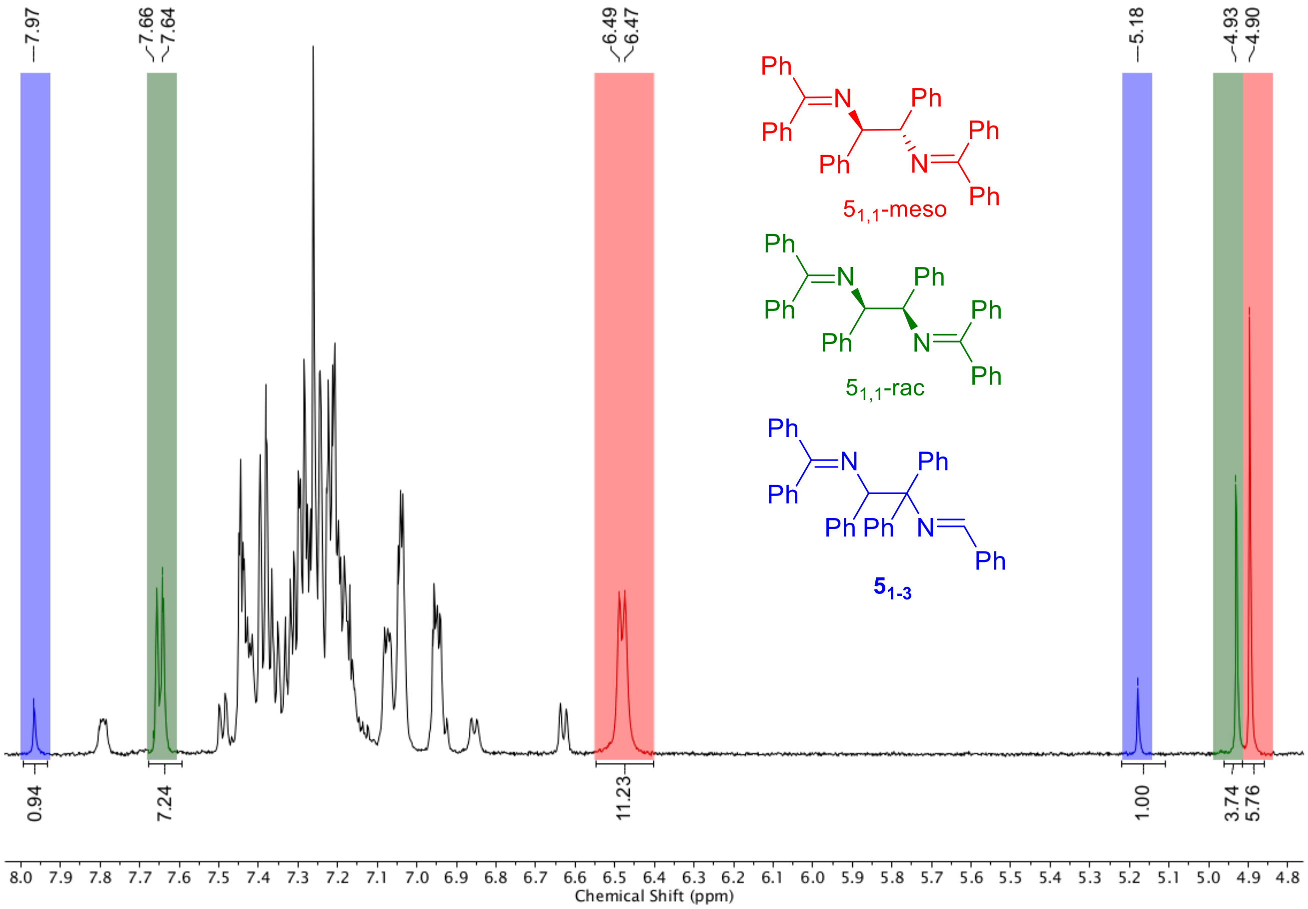

Figure S16: H[1] NMR (500 MHz, $CDCl_3$) spectrum of dimerization of **2-Na** with $AgPF_6$. The three isomers dimers **$5_{1\text{-}1}$-rac $5_{1\text{-}1}$-meso, and $5_{1\text{-}3}$** are color coded green, red, and blue respectively.

Electrochemical Measurements:

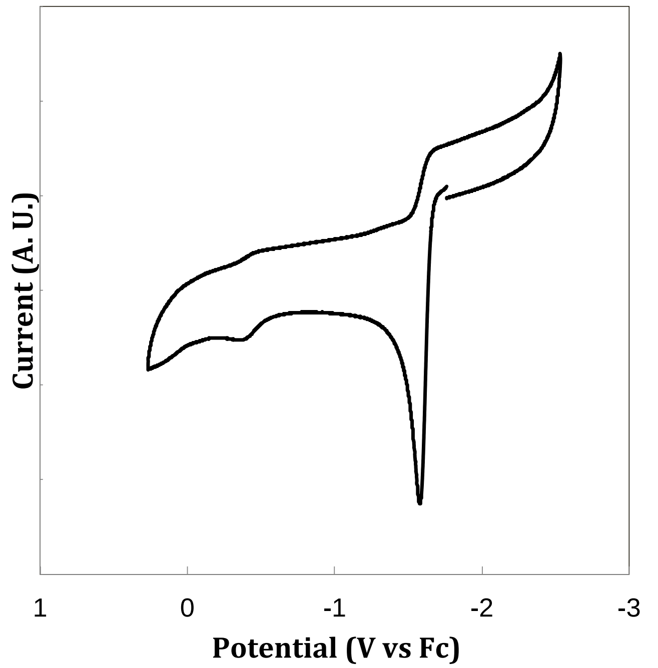

Figure S17a: Cyclic Voltammogram of **2-Li** (1 mmol*L^{-1}) in DME using [^{n}NPr$_4$][BAr$^F_4$] (100 mmol*L^{-1}) as the electrolyte. Scan Rate: 100 mV*s^{-1}. OCP: −1.76 V versus Fc/Fc$^+$. E_{pc}: −1.58 V versus Fc/Fc$^+$.

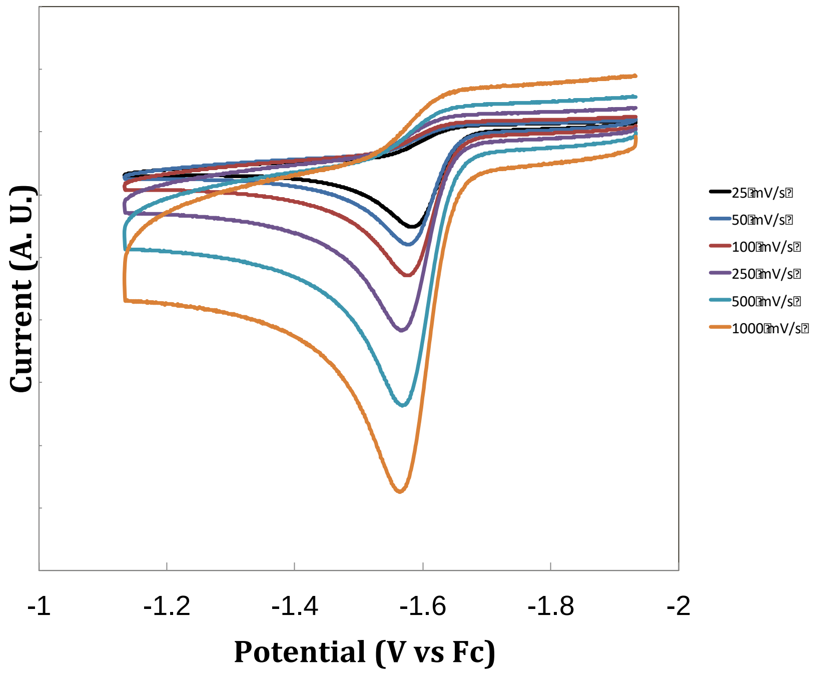

Figure S17b: Scan rate dependence of **2-Li** (1 mmol*L^{-1}) in DME using [^{n}NPr$_4$][BAr$^F_4$] (100 mmol*L^{-1}) as the electrolyte.

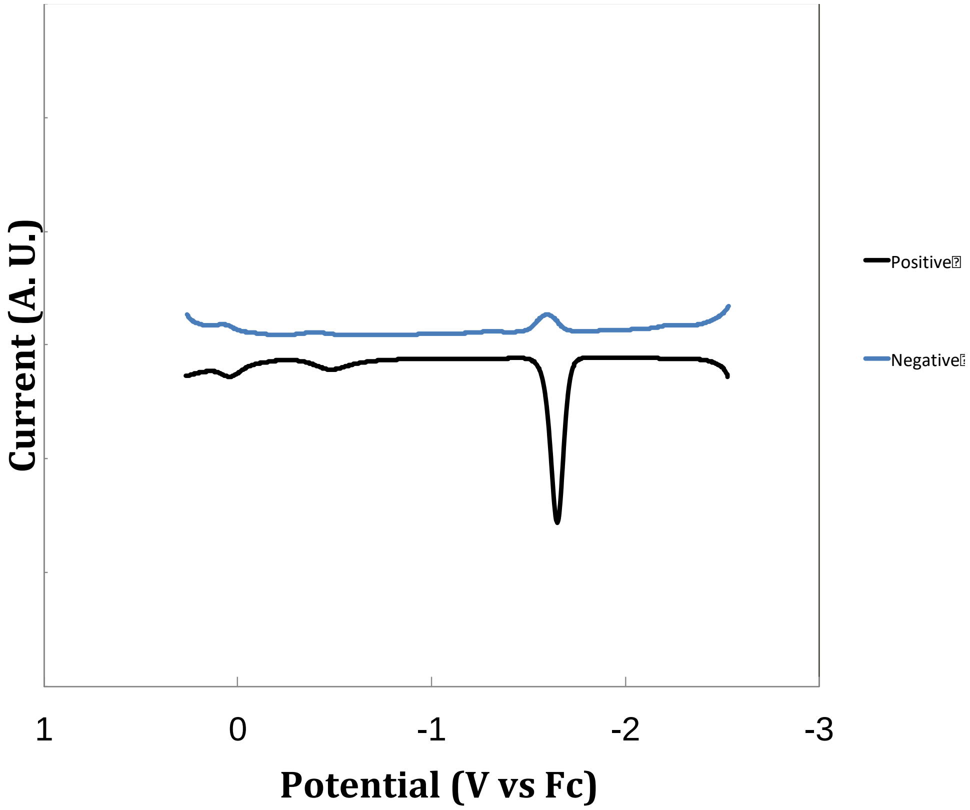

Figure S17c: Differential Pulse Voltammetry of **2-Li** (1 mmol*L^{-1}) in DME using [$^{n}NPr_4$][$BAr^F{}_4$] (100 mmol*L^{-1}) as the electrolyte.

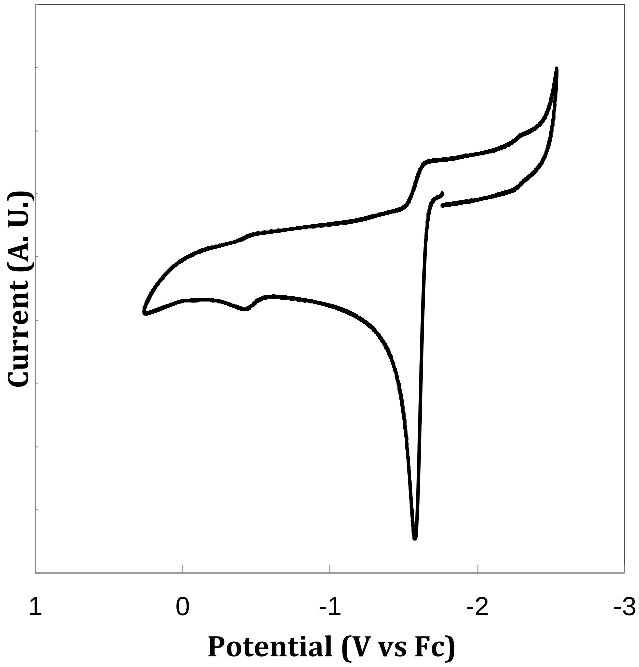

Figure S18a: Cyclic Voltammogram of **2-Na** (1 mmol*L^{-1}) in DME using [^{n}NPr$_4$][BAr$^F_4$] (100 mmol*L^{-1}) as the electrolyte. Scan Rate: 100 mV*s^{-1}. OCP: −1.76 V versus Fc/Fc$^+$. E_{pc}: −1.58 V versus Fc/Fc$^+$.

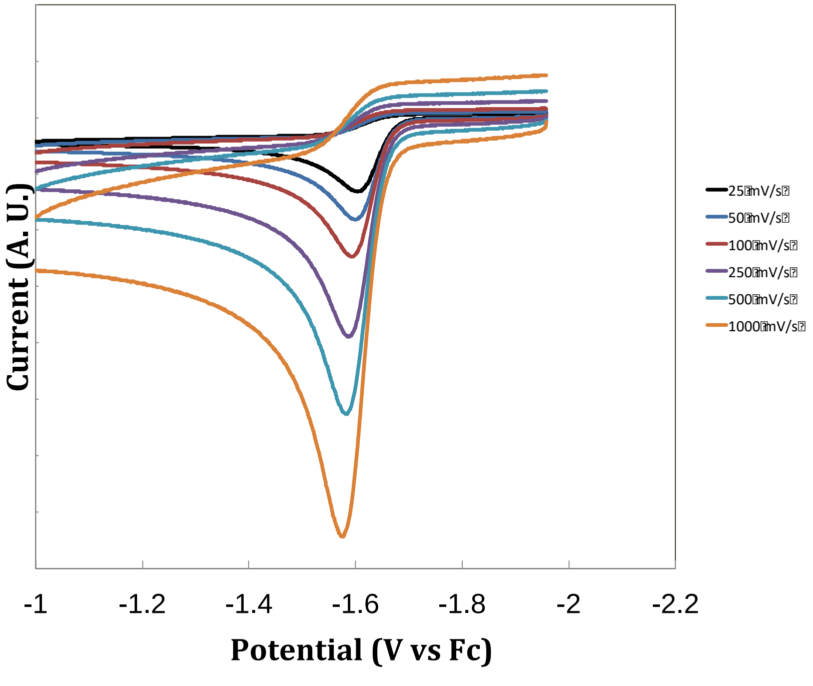

Figure S18b: Scan rate dependence of **2-Na** (1 mmol*L^{-1}) in DME using [^{n}NPr$_4$][BAr$^F_4$] (100 mmol*L^{-1}) as the electrolyte.

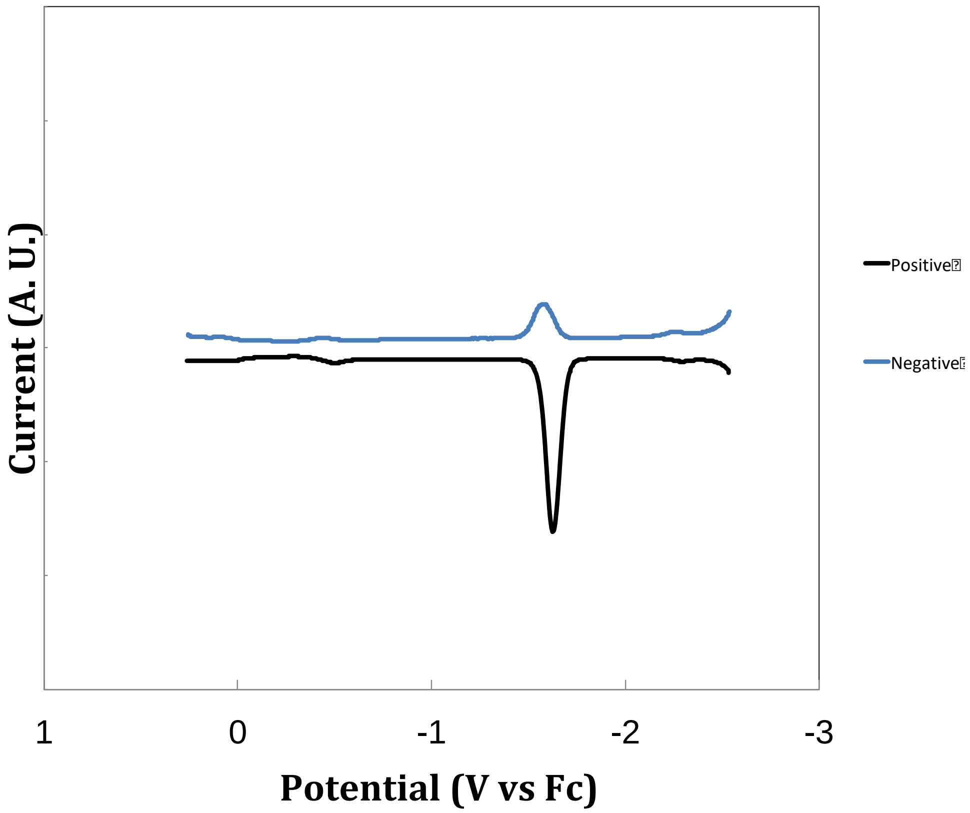

Figure S18c: Differential Pulse Voltammetry of **2-Na** (1 mmol*L^{-1}) in DME using [^{n}NPr$_4$][BAr$^F{}_4$] (100 mmol*L^{-1}) as the electrolyte.

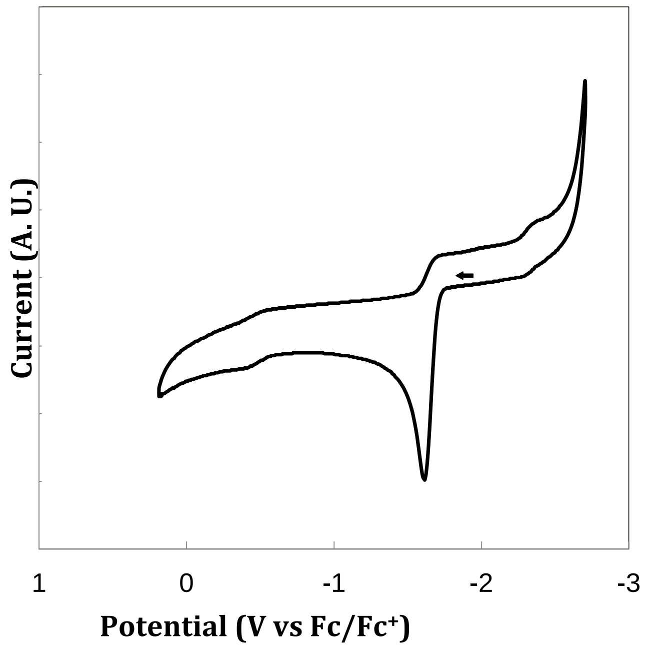

Figure S19a: Cyclic Voltammogram of **2-K** (1 mmol*L^{-1}) in DME using [$^{n}NPr_4$][$BAr^F{}_4$] (100 mmol*L^{-1}) as the electrolyte. Scan Rate: 100 mV*s^{-1}. OCP: −1.84 V versus Fc/Fc$^+$. E_{pc}: −1.58 V versus Fc/Fc$^+$.

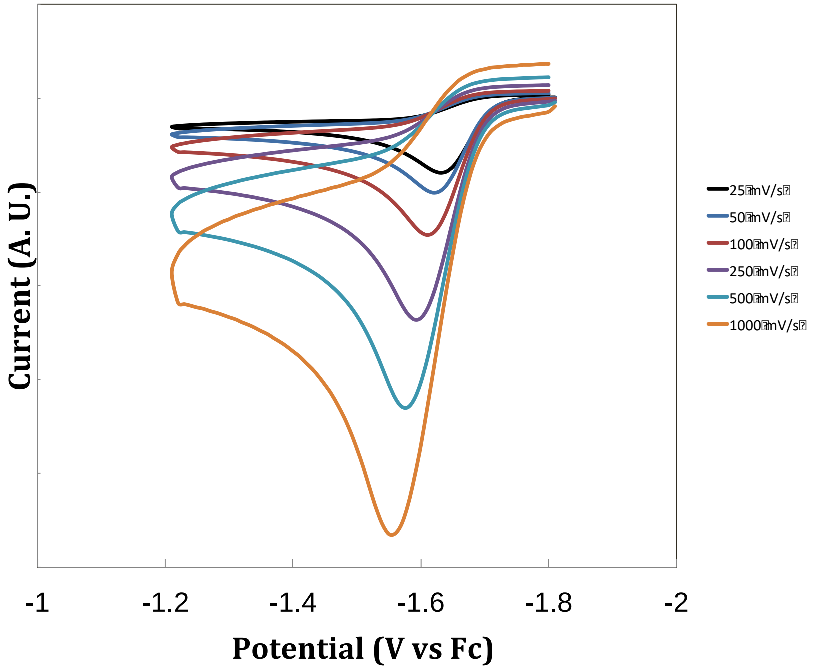

Figure S19b: Scan rate dependence of **2-K** (1 mmol*L^{-1}) in DME using [$^{n}NPr_4$][$BAr^F{}_4$] (100 mmol*L^{-1}) as the electrolyte.

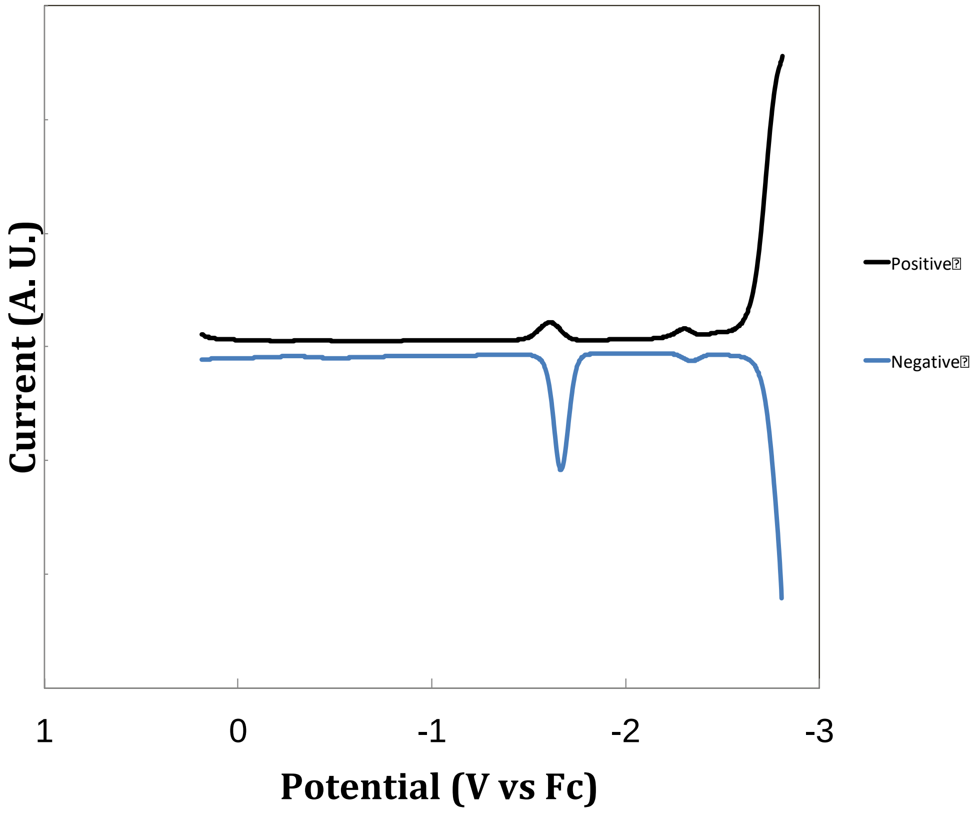

Figure S19c: Differential Pulse Voltammetry of **2-K** (1 mmol*L^{-1}) in dimethoxyethane using [^{n}NPr$_4$][BAr$^F_4$] (100 mmol*L^{-1}) as the electrolyte.

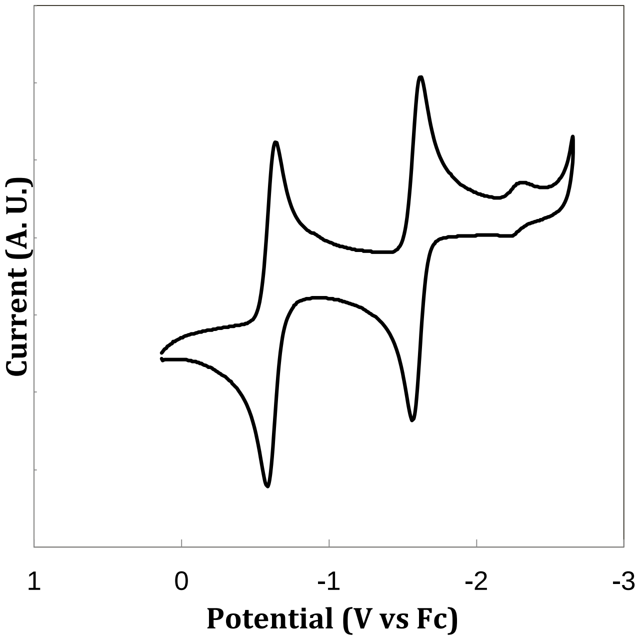

Figure S20a: Cyclic Voltammogram of **4-Na** (1 mmol*L^{-1}) in DME using [^{n}NPr$_4$][BAr$^F{}_4$] (100 mmol*L^{-1}) as the electrolyte. Scan Rate: 100mV*s^{-1}. OCP: −1.69 V versus Fc/Fc$^+$. $E_{1/2}$: −1.60 V versus Fc/Fc$^+$ & $E_{1/2}$: −0.61 V versus Fc/Fc$^+$.

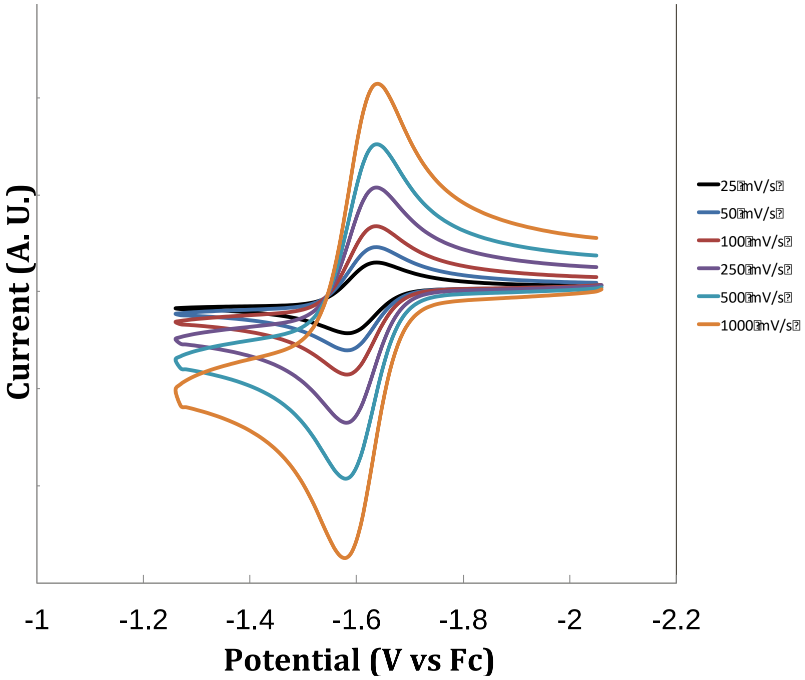

Figure S20b: Scan rate dependence of **4-Na** (1 mmol*L^{-1}) in DME using [^{n}NPr$_4$][BAr$^F{}_4$] (100 mmol*L^{-1}) as the electrolyte. Wave centered at −1.60 V versus Fc/Fc$^+$.

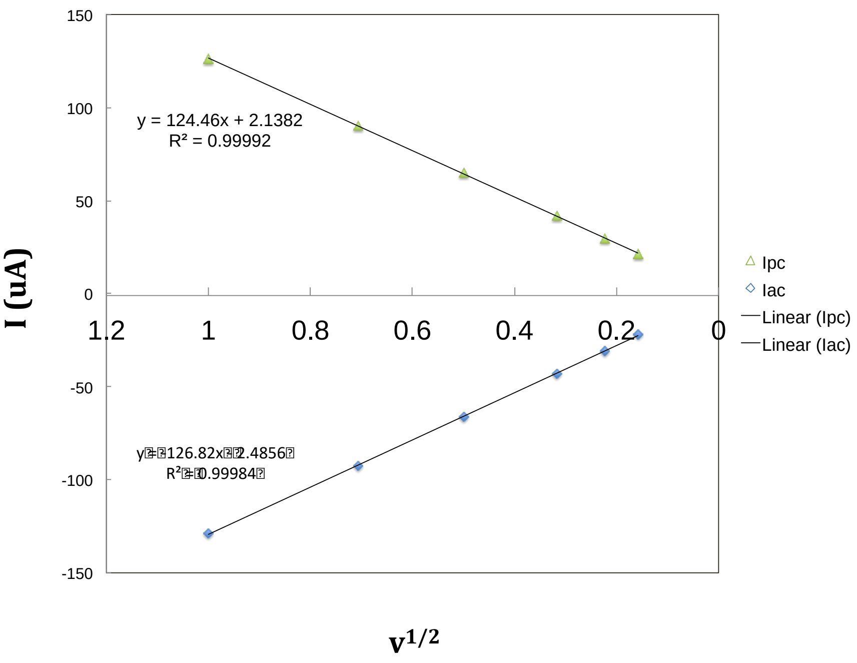

Figure S20c: Plot of the square of the scan rate ($\nu^{1/2}$) vs the current in μA of **4-Na** (1 mmol*L^{-1}) in DME using [^{n}NPr$_4$][BAr$^F_4$] (100 mmol*L^{-1}) as the electrolyte. Wave centered at −1.60 V vs (Fc/Fc$^+$).

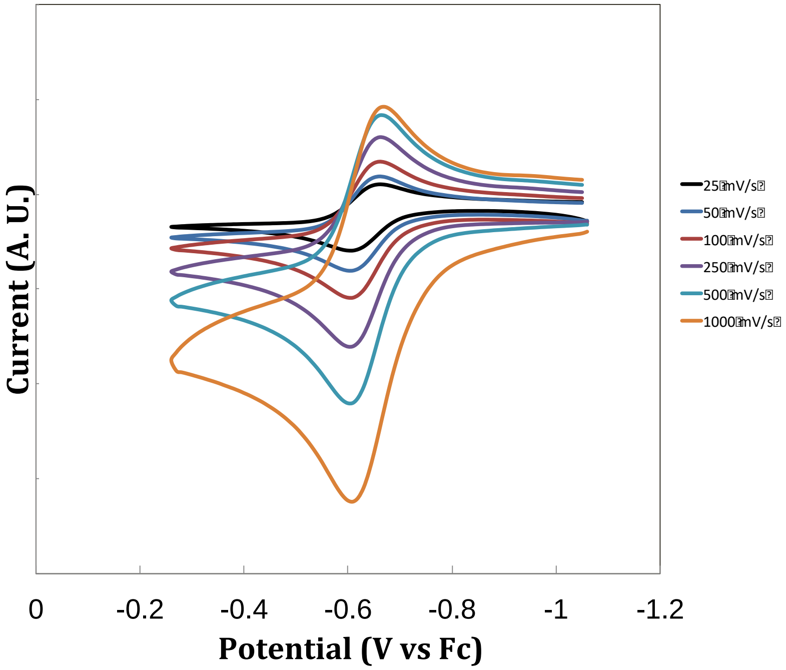

Figure S20d: Scan rate dependence of **4-Na** (1 mmol*L^{-1}) in DME using [^{n}NPr$_4$][BAr$^F_4$] (100 mmol*L^{-1}) as the electrolyte. Wave centered at −0.61 V versus Fc/Fc$^+$.

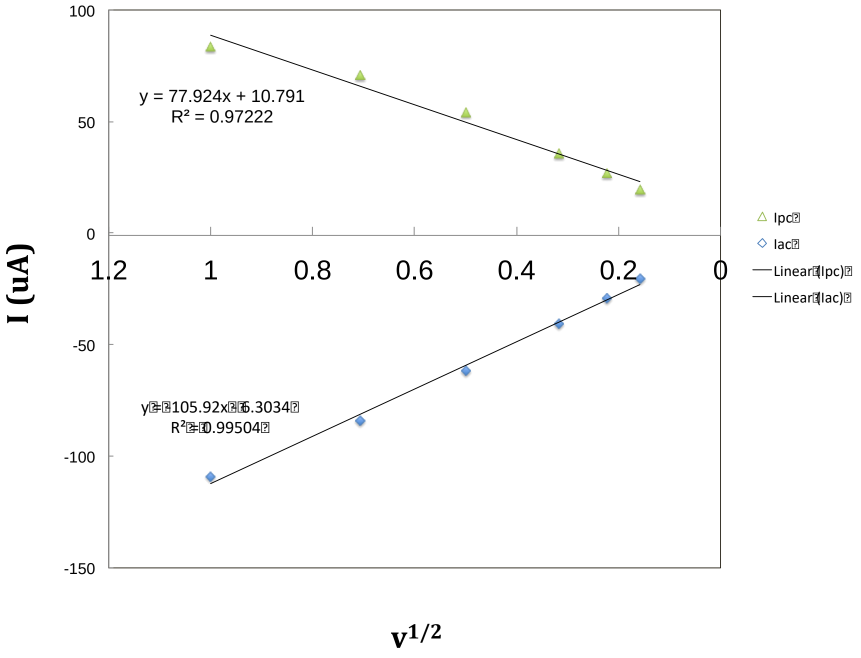

Figure S20e: Plot of the square of the scan rate ($\nu^{1/2}$) vs the current in μA of **4-Na** (1 mmol*L^{-1}) in DME using [$^{n}NPr_4$][$BAr^F{}_4$] (100 mmol*L^{-1}) as the electrolyte. Wave centered at −0.61 V versus Fc/Fc$^+$.

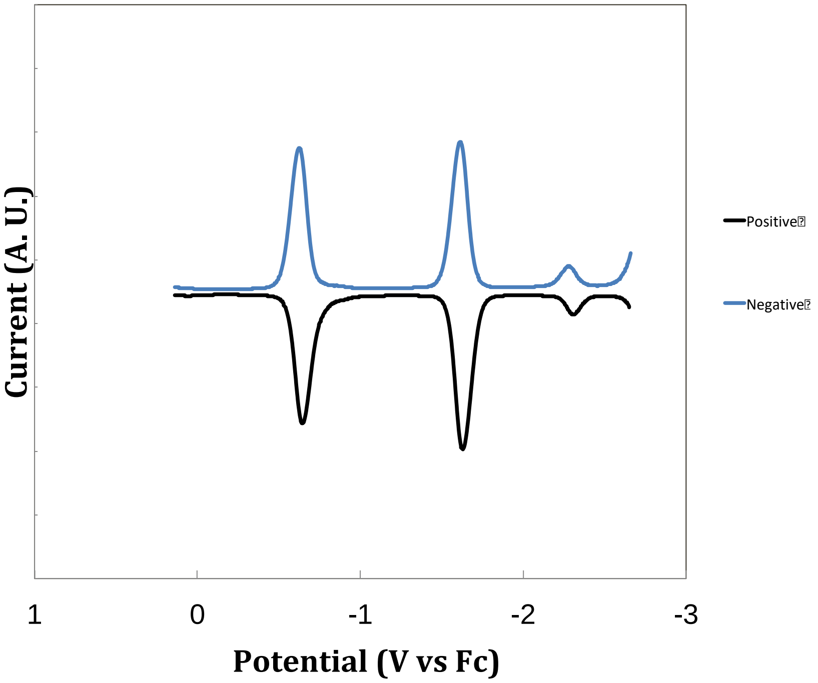

Figure S20f: Differential Pulse Voltammetry of **4-Na** (1 mmol*L^{-1}) in DME using [$^{n}NPr_4$][$BAr^F{}_4$] (100 mmol*L^{-1}) as the electrolyte.

UV-vis:

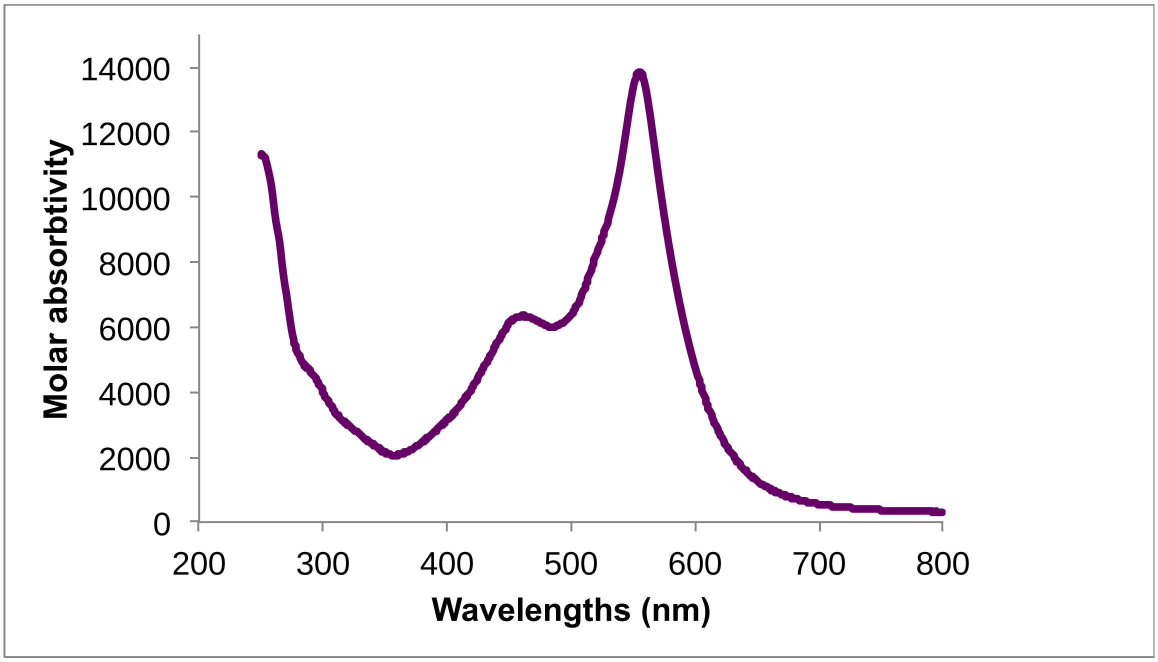

Figure S21: UV-vis spectrum of **4-Na** in DME.

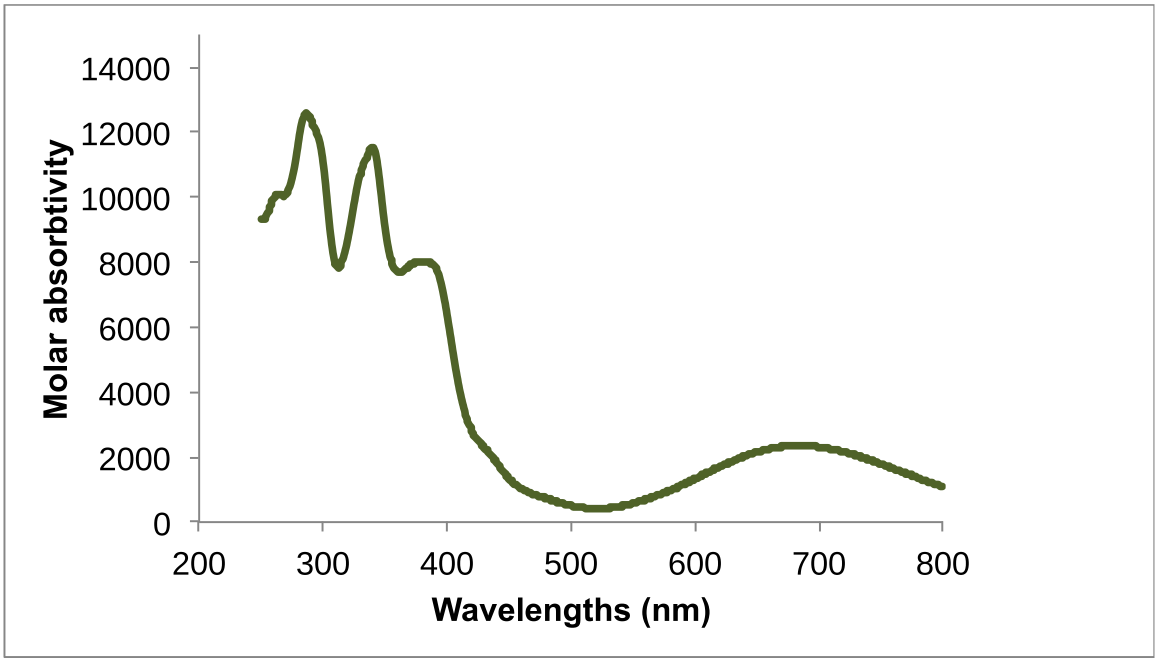

Figure S22: UV-vis spectrum of **6** in DME.

X-ray crystal structures:

Compound	2-Li	2-Na	2-K	4-Na	6
**Empirical formula**	$C_{64}H_{92}Li_2N_2O_{12}$	$C_{32}H_{46}NNaO_6$	$C_{32}H_{40}KNO_6$	$C_{38}H_{50}NNaO_6$	$C_{26}H_{20}N$
**Formula weight**	1095.27	563.69	573.75	639.78	346.43
**Temperature/K**	100	100	100	100	100
**Crystal system**	monoclinic	triclinic	monoclinic	monoclinic	monoclinic
**Space group**	$P2_1/c$	$P\bar{1}$	$P2_1$	$P2_1/c$	$P2_1$
**a**	11.6347(5)Å	11.6153(5)Å	9.4575(6)Å	17.7615(7)Å	9.8522(9)Å
**b**	18.1467(9)Å	11.6198(5)Å	13.1739(8)Å	11.8703(4)Å	7.7653(7)Å
**c**	31.5962(14)Å	12.6450(6)Å	12.1272(7)Å	34.9201(13)Å	12.2084(11)Å
**α**	90°	104.751(2)°	90°	90°	90°
**β**	95.756(2)°	95.937(2)°	101.138(2)°	100.040(2)°	95.841(5)°
**γ**	90°	99.774(2)°	90°	90°	90°
**Volume**	6637.3(5)Å^3	1607.08(13)Å^3	1482.49(16)Å^3	7249.6(5)Å^3	929.16(15)Å^3
**Z**	4	2	2	8	2
**d_{calc}**	1.096 g/cm^3	1.165 g/cm^3	1.285 g/cm^3	1.172 g/cm^3	1.238 g/cm^3
**F(000)**	2368.0	608.0	612.0	2752.0	366.0
**Crystal size, mm**	0.49 × 0.43 × 0.19	0.33 × 0.17 × 0.03	0.5 × 0.2 × 0.08	0.23 × 0.11 × 0.07	0.42 × 0.12 × 0.1
**2θ range for data collection**	5.772 - 55.152°	3.37 - 55.222°	5.896 - 55.084°	2.368 - 55.046°	3.354 - 55.202°
**Reflections collected**	204102	40235	53265	155046	20557
**Goodness-of-fit on F^2**	1.105	1.028	1.064	1.015	1.061
**Final R indexes [I>=2σ (I)]**	R_1 = 0.0799, wR_2 = 0.1849	R_1 = 0.0471, wR_2 = 0.1206	R_1 = 0.0231, wR_2 = 0.0581	R_1 = 0.0414, wR_2 = 0.0958	R_1 = 0.0310, wR_2 = 0.0761

Table S1: X-ray collection parameters.

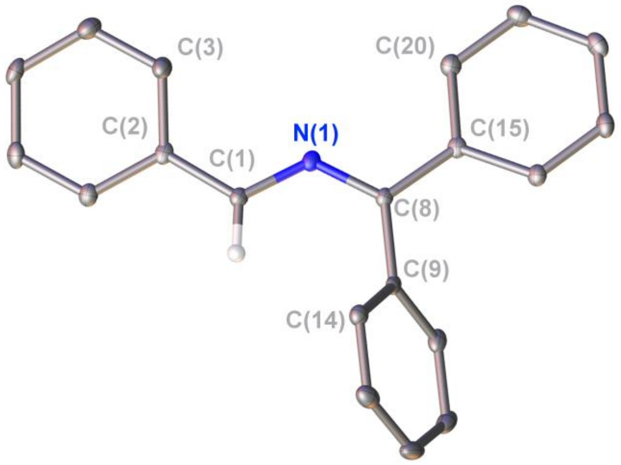

Figure S23a: Thermal ellipsoid plot of one of the 2-azaallyl portions of **2-Li** [Li(DME)$_3$][1,3,3-triphenyl 2-azaallyl anion] at 30% probability level. Selected bond distances (Å): N(1)–C(1): 1.284(15)- 1.325(2), N(1)–C(8): 1.337(9)- 1.347(5), C(1)–C(2): 1.441(2)-1.443(10), C(8)–C(15): 1.451(15)- 1.473(6), C(8)–C(9): 1.485(5)-1.531(10). Selected bond angles (degrees): C(8)–N(1)–C(1): 123.9(8)- 125.90(16), N(1)–C(1)–C(2): 120.5(6)- 123.8(9), N(1)–C(8)–C(15): 117.1(4)- 119.5(9), N(1)–C(8)–C(9): 119.8(7)- 122.7(3). Selected dihedral angles (degrees): C(2)–C(1)–N(1)–C(8): -175.01- 178.92, C(3)–C(2)–C(1)–N(1): -7.38- 5.67, C(15)–C(8)–N(1)–C(1): -173.88- 178.93, C(20)–C(15)–C(8)–N(1): -6.78- 1.17, C(16)–C(15)–C(8)–N(1): -93.37- -55.86.

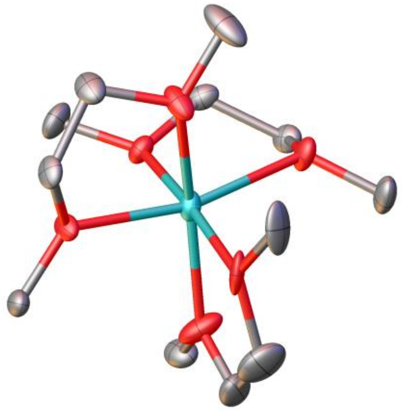

Figure S23b: Thermal ellipsoid plot of one of the cationic portions of **2-Li** at 30% probability level. H-atoms and disorder removed for clarity.

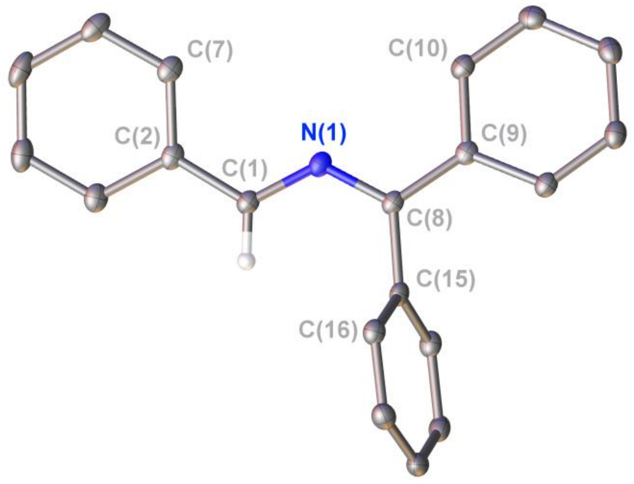

Figure S23a: Thermal ellipsoid plot of the 2-azaallyl portion of **2-Na** [Na(DME)$_3$][1,3,3-triphenyl 2-azaallyl anion] at 30% probability level. Phenyl H-atoms and cation removed for clarity. Selected bond distances (Å): N(1)–C(1): 1.3250(18), N(1)–C(8): 1.3365(17), C(1)–C(2): 1.4353(18), C(8)–C(9): 1.445(2), C(8)–C(15): 1.4941(19). Selected bond angles (degrees): C(8)–N(1)–C(1): 124.46(13), N(1)–C(1)–C(2): 120.94(13), N(1)–C(8)–C(9): 117.92(12), N(1)–C(8)–C(15): 122.11(12). Selected dihedral angles (degrees): C(2)–C(1)–N(1)–C(8): 178.70, C(7)–C(2)–C(1)–N(1): -4.34, C(9)–C(8)–N(1)–C(1): 176.15, C(10)–C(9)–C(8)–N(1): -7.45, C(16)–C(15)–C(8)–N(1): -69.33.

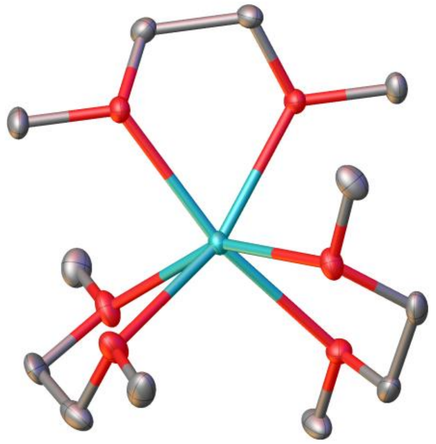

Figure S23b: Thermal ellipsoid plot of the cationic portion of **2-Na** at 30% probability level. H-atoms removed for clarity.

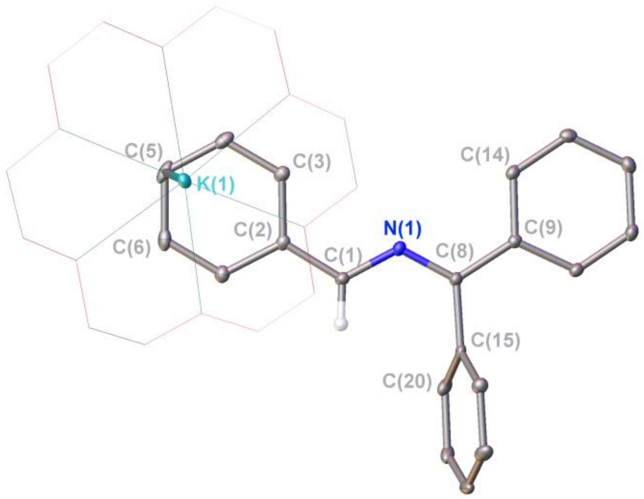

Figure S24a: Thermal ellipsoid plot of **2-K** [K(18-crown-6)][1,3,3-triphenyl 2-azaallyl anion] at 30% probability level. 18-crown-6 is represented in wireframe and phenyl and 18-crown-6 H-atoms are removed for clarity. Selected bond distances (Å): N(1)–C(1): 1.3346(19), N(1)–C(8): 1.3353(18), C(1)–C(2): 1.433(2), C(8)–C(9): 1.451(2), C(8)–C(15): 1.491(2), K(1)–C(5): 1.4941(19), K(1)–C(6′): 3.4377(18). Selected bond angles (degrees): C(8)–N(1)–C(1): 123.90(13), N(1)–C(1)–C(2): 120.67(13), N(1)–C(8)–C(9): 119.07(13), N(1)–C(8)–C(15): 121.76(13). Selected dihedral angles (degrees): C(2)–C(1)–N(1)–C(8): -178.90, C(3)–C(2)–C(1)–N(1): -6.48, C(9)–C(8)–N(1)–C(1): 177.26, C(14)–C(9)–C(8)–N(1): 2.23, C(20)–C(15)–C(8)–N(1): 75.00.

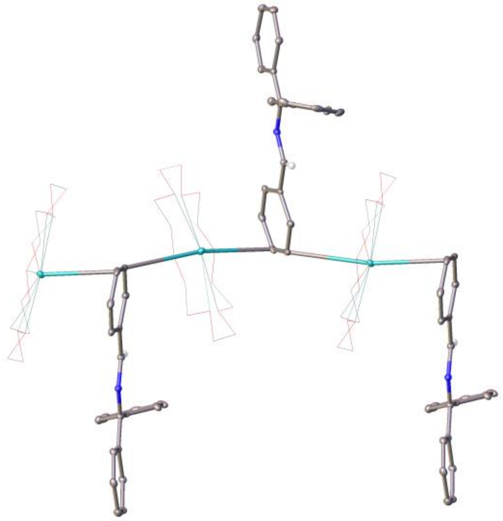

Figure S24b: Thermal ellipsoid plot of **2-K** showing the 1-d polymeric structure.

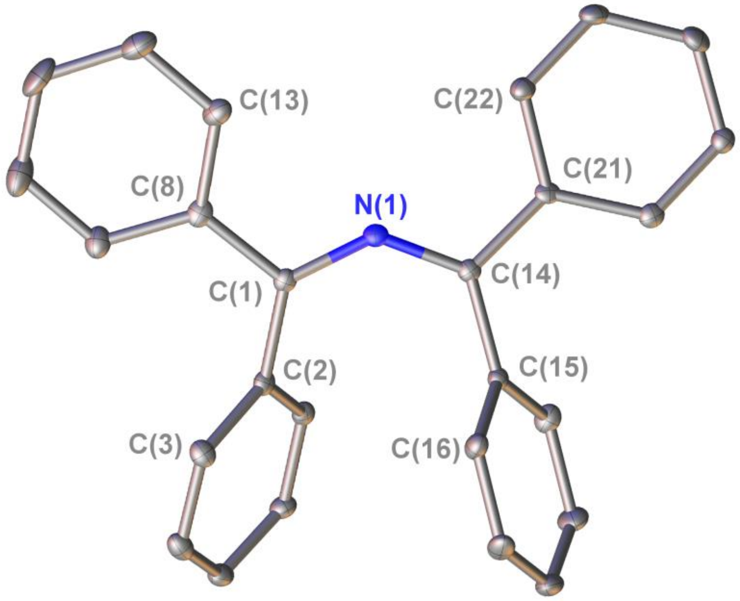

Figure S25a: Thermal ellipsoid plot of one of the 2-azaallyl portions of **4-Na** [Na(DME)$_3$][1,1,3,3-tetraphenyl 2-azaallyl anion] at 30% probability level. H-atoms and cation removed for clarity. Selected bond distances (Å): N(1)–C(1): 1.335(2)-1.335(2), N(1)–C(14): 1.333(2)-1.342(2), C(1)–C(8): 1.464(2)-1.471(2), C(14)–C(21): 1.464(2)-1.464(2), C(1)–C(2): 1.487(2)-1.489(2), C(14)–C(15): 1.486(2)-1.494(2). Selected bond angles (degrees): C(1)–N(1)–C(14): 131.6(1)-132.9(1), N(1)–C(1)–C(8): 114.8(1)-115.4(1), N(1)–C(14)–C(21): 114.8(1)-115.7(1), N(1)–C(1)–C(2): 124.8(1)-125.1(1), N(1)–C(14)–C(15): 125.0(1)-125.4(1). Selected dihedral angles (degrees): C(8)–C(1)–N(1)–C(14): -174.4- -174.8, C(13)–C(8)–C(1)–N(1): 4.6-7.8, C(21)–C(14)–N(1)–C(1): -170.5- -174.1, C(22)–C(21)–C(14)–N(1): 5.8-10.1, C(3)–C(2)–C(1)–N(1): -129.9- 46.4, C(16)–C(15)–C(14)–N(1): -122.9- 43.4.

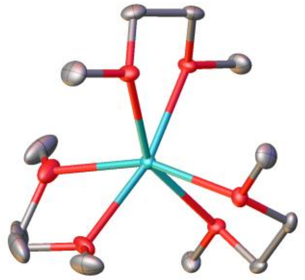

Figure S25b: Thermal ellipsoid plot of the cationic portion of **4-Na** at 30% probability level. H-atoms removed for clarity.

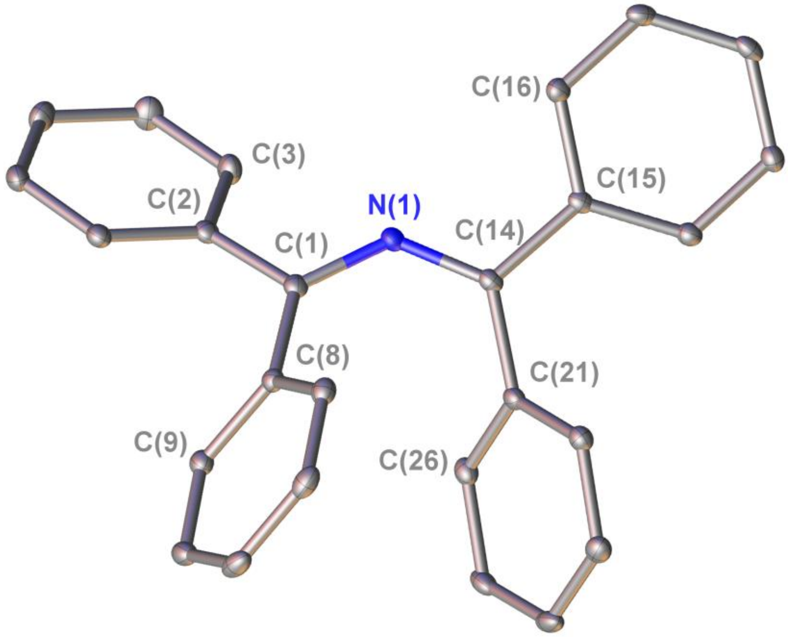

Figure S26: Thermal ellipsoid plot of **6** 1,1,3,3-tetraphenyl 2-azaallyl radical at the 30% probability level. H-atoms removed for clarity. Selected bond distances (Å): N(1)–C(1): 1.336(2), N(1)–C(14): 1.330(2), C(1)–C(2): 1.470(2), C(14)–C(15): 1.473(2), C(1)–C(8): 1.486(2), C(14)–C(21): 1.482(2). Selected bond angles (degrees): C(1)–N(1)–C(14): 128.87(14), N(1)–C(1)–C(2): 114.55(14), N(1)–C(14)–C(15): 115.75(14), N(1)–C(1)–C(8): 125.17(14), N(1)–C(14)–C(21): 124.27(14). Selected dihedral angles (degrees): C(2)–C(1)–N(1)–C(14): -162.24, C(3)–C(2)–C(1)–N(1): 25.26, C(15)–C(14)–N(1)–C(1): -166.46, C(16)–C(15)–C(14)–N(1): 15.72, C(9)–C(8)–C(1)–N(1): -140.55, C(16)–C(15)–C(14)–N(1): 42.09.

Computational Details and Supplementary Data

Density Functional Theory (DFT) calculations were carried out using the Gaussian '16 suite (revision A.03).[12] The hybrid functional combining Becke's 3-parameter exchange functional combined with the Lee-Yang-Parr correlation functional (B3LYP) was employed. 6-31-G* basis sets as implemented in Gaussian 16 were employed for light atoms (H, C, N). Iodine atoms were treated with a 28-electron small core pseudo-potential associated with the Stuttgart-Bonn variety of natural orbital incorporating quasi-relativistic effects. Structures were optimized without constraints; convergence criteria were kept to their default values. All stationary points were verified to possess 0 (reaction intermediates) by analytical vibrational frequency calculations. Thermochemistry calculations were performed at 298.15 K. Molecular orbitals were rendered using Gaussview software.

Theory (previously reported here[13, 14]):

$$\Delta G^* = \frac{\lambda}{4}\left(1 + \frac{\Delta G_{rel}}{\lambda}\right)^2$$

Equation S1: Estimation of the free energy of the transition state of electron transfer (ΔG^*) from the reorganization energy (λ) and from the free energy of electron transfer (ΔG^*).

$$\lambda = \lambda_i + \lambda_o;\ \lambda_i \gg \lambda_o;\ \lambda \approx \lambda_i$$

Equation S2: Approximation of the reorganization energy (λ) being primarily the internal reorganization energy (λ_i) rather than the solvent reorganization energy (λ_o) in organic solvent.

$$\Delta G^* = \frac{\lambda_i}{4}\left(1 + \frac{\Delta G_{rel}}{\lambda_i}\right)^2$$

Equation S3: Replacement of total reorganization energy for internal reorganization energy (λ_i) in equation S#.

$$\lambda_i = \frac{\lambda_i(D) + \lambda_i(A)}{2}$$

Equation S4: Determination of the internal reorganization energy (λ_i) from the average of the internal reorganization energy of the electron donor ($\lambda_i(D)$) and the internal reorganization energy of the electron acceptor ($\lambda_i(A)$)

$$\lambda_i(D\ or\ A) = \left(E_S(R_P) - E_S(R_S)\right) + \left(E_P(R_S) - E_P(R_P)\right)$$

Equation S5: Definition of the internal reorganization energy of either the electron donor ($\lambda_i(D)$) or the electron acceptor ($\lambda_i(A)$) as related to the sum of the differences in the free energies of the starting material in its optimized

geometry ($E_S(R_S)$) and the starting material in the geometry of the product ($E_S(R_P)$) and the differences in the free energies of the product in its optimized geometry ($E_P(R_P)$) and the product in the geometry of the starting material ($E_P(R_S)$).

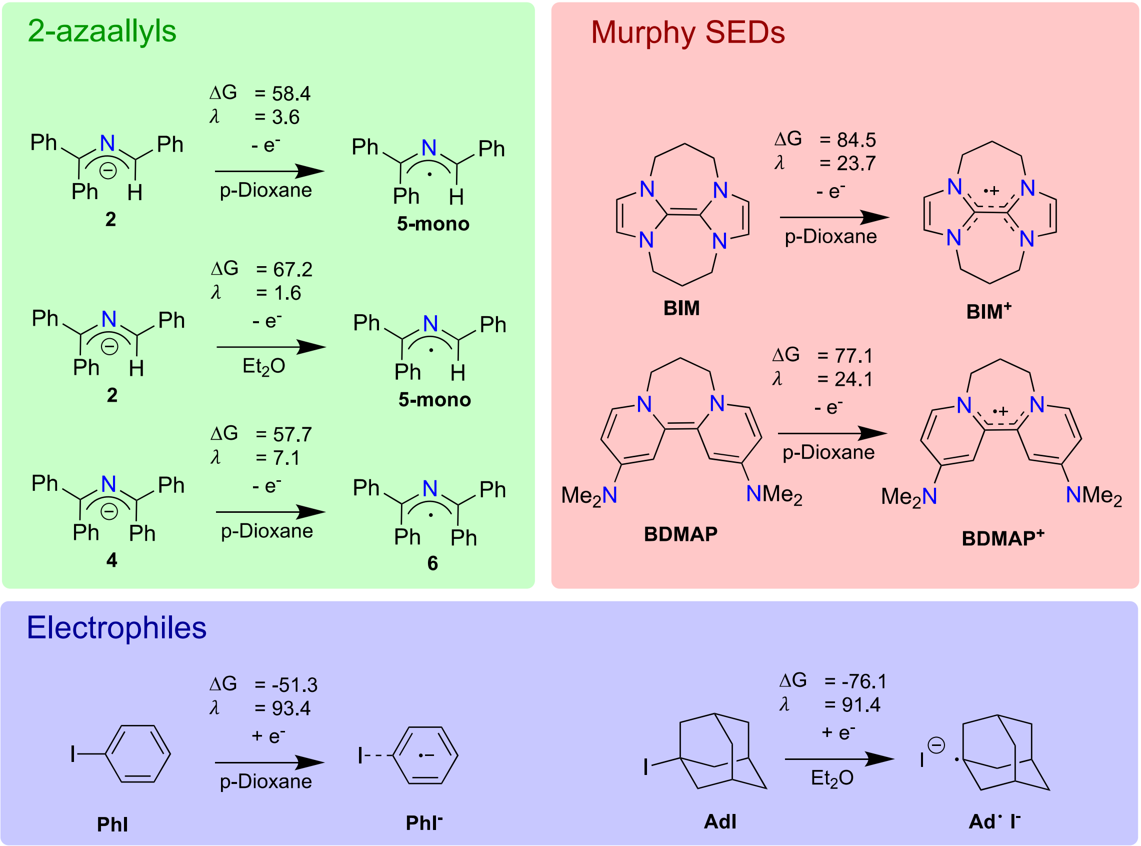

Scheme S1: The reorganization energies and free energies of electron transfer determined by computation. The compounds are divided by color: green for 2-azaallyl compounds, red for compounds previously described by Murphy, and blue for electrophiles. The solvent field used for the calculation is placed underneath the arrow for electron transfer.

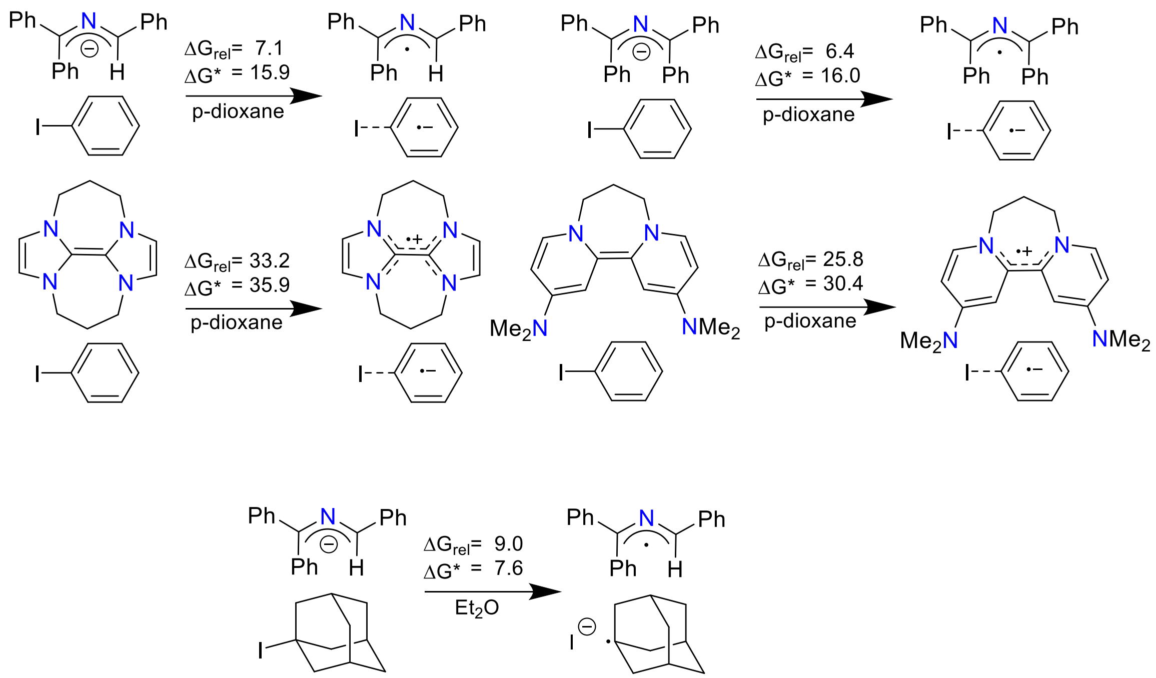

Scheme S2: The calculated change in free energy ΔG_{rel} and the predicted barrier ΔG^* of electron transfer between the SEDs and the substrates. These values were calculated using the values in Scheme S# and the equations above. As a general trend the azaallyl anions have both lower ΔG_{rel} and ΔG^* of their electron transfer compared to the previously reported SEDs.

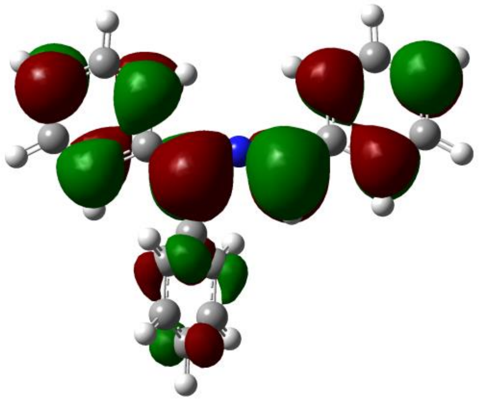

Figure S27: HOMO of **2** showing clear non-bonding character in the 2-azallyl moiety.

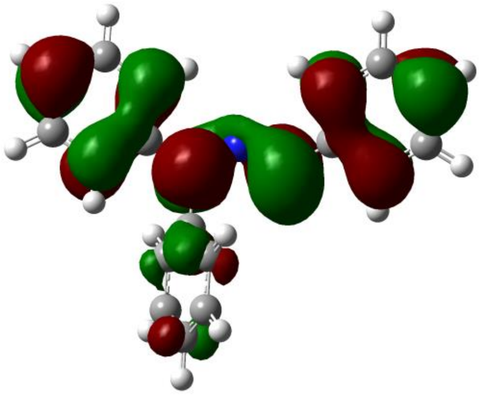

Figure S28: SOMO of **5-mono** showing clear non-bonding character in the 2-azallyl moiety.

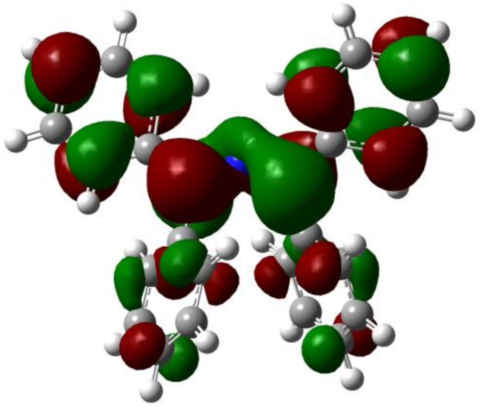

Figure S29: HOMO of **4** showing clear non-bonding character in the 2-azallyl moiety.

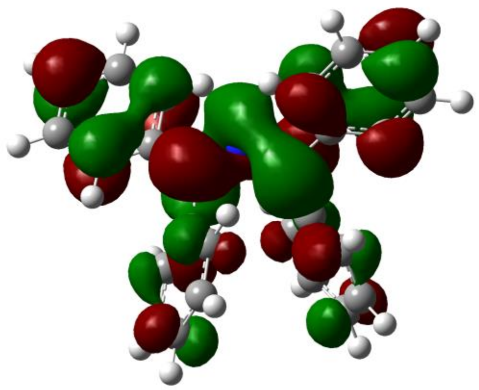

Figure S30: SOMO of **6** showing clear non-bonding character in the 2-azallyl moiety.

Computed optimized geometries and Energies:

Compound: **2**
Solvent: p-Dioxane
Energy: -826.640413469 hartrees
Energy with thermal free energy correction: -826.390594 hartrees

7	0.492815000	-0.376843000	0.034515000
6	1.488561000	0.491457000	-0.035669000
1	1.312544000	1.570838000	-0.109963000
6	2.870133000	0.073101000	-0.038605000
6	3.908389000	1.036271000	-0.168907000
1	3.637769000	2.085978000	-0.268949000
6	5.252840000	0.670305000	-0.175479000
1	6.014836000	1.440566000	-0.278671000
6	5.631596000	-0.674906000	-0.052310000
1	6.679749000	-0.961128000	-0.056933000
6	4.621188000	-1.642829000	0.080281000
1	4.891547000	-2.692498000	0.180563000
6	3.276733000	-1.284754000	0.090129000
1	2.507291000	-2.042283000	0.200294000
6	-0.820577000	-0.104046000	0.032510000
6	-1.746975000	-1.229907000	0.036589000
6	-1.291016000	-2.565069000	-0.164484000
1	-0.232238000	-2.719175000	-0.340977000
6	-2.160801000	-3.650147000	-0.139306000
1	-1.763181000	-4.650568000	-0.300938000
6	-3.537490000	-3.474467000	0.078971000
1	-4.214777000	-4.324050000	0.091982000
6	-4.010878000	-2.171377000	0.280371000
1	-5.070601000	-2.001672000	0.462184000
6	-3.144139000	-1.078475000	0.262291000
1	-3.550119000	-0.087882000	0.441226000
6	-1.342596000	1.294183000	0.010473000
6	-0.976894000	2.229031000	1.002013000
1	-0.319602000	1.910317000	1.806204000
6	-1.449305000	3.543101000	0.978654000
1	-1.153620000	4.234734000	1.764222000
6	-2.305188000	3.970043000	-0.043945000
1	-2.673348000	4.992410000	-0.063780000
6	-2.677636000	3.061578000	-1.040940000
1	-3.336914000	3.377628000	-1.846223000
6	-2.200240000	1.748483000	-1.013377000
1	-2.493267000	1.055226000	-1.797430000

Compound: **2**
Solvent: Et_2O
Energy: -826.655657720 hartrees
Energy with thermal free energy correction: -826.406239 hartrees

7	-0.488550000	-0.398088000	-0.026723000
6	-1.488360000	0.469085000	0.038045000
1	-1.312312000	1.548858000	0.103324000
6	-2.869508000	0.049093000	0.038668000
6	-3.908727000	1.013318000	0.158357000
1	-3.639293000	2.063985000	0.250897000
6	-5.253268000	0.647155000	0.163390000
1	-6.015454000	1.418000000	0.258113000
6	-5.631672000	-0.699224000	0.049186000
1	-6.679665000	-0.985286000	0.052777000
6	-4.621241000	-1.668190000	-0.073224000
1	-4.891254000	-2.718402000	-0.166645000
6	-3.276236000	-1.309892000	-0.081462000
1	-2.508394000	-2.070278000	-0.183697000
6	0.821302000	-0.112628000	-0.027685000
6	1.764892000	-1.222950000	-0.033050000
6	1.331507000	-2.569562000	0.143354000
1	0.273628000	-2.748704000	0.301264000
6	2.222676000	-3.637564000	0.117984000
1	1.842313000	-4.647467000	0.259803000
6	3.598992000	-3.432267000	-0.076316000
1	4.292511000	-4.268371000	-0.090048000
6	4.050666000	-2.117634000	-0.251920000
1	5.109752000	-1.925418000	-0.412499000
6	3.162813000	-1.041527000	-0.232951000
1	3.552789000	-0.040480000	-0.387805000
6	1.323758000	1.295802000	-0.009407000
6	1.002281000	2.199062000	-1.043226000
1	0.395509000	1.851660000	-1.875158000
6	1.454079000	3.520987000	-1.025069000
1	1.195051000	4.189941000	-1.842210000
6	2.242969000	3.983510000	0.035213000
1	2.595764000	5.011156000	0.050902000
6	2.570031000	3.105847000	1.074513000
1	3.177878000	3.451176000	1.907373000
6	2.113002000	1.784634000	1.051254000
1	2.371295000	1.113919000	1.866748000

Compound: **4**
Solvent: p-dioxane
Energy: -1057.69868386 hartrees
Energy with thermal free energy correction: -1057.374052 hartrees

7	-0.000013000	-0.890056000	0.000014000
6	1.228136000	-0.365650000	0.021653000
6	1.559993000	1.062646000	0.290743000
6	1.011086000	1.738684000	1.400633000
1	0.314755000	1.209946000	2.044524000
6	1.345847000	3.062587000	1.688305000
1	0.906068000	3.551194000	2.554598000
6	2.243591000	3.761551000	0.872026000
1	2.502157000	4.793558000	1.093855000
6	2.799446000	3.111458000	-0.235632000
1	3.492917000	3.640129000	-0.885901000
6	2.462215000	1.785749000	-0.517653000
1	2.899302000	1.296654000	-1.384235000
6	2.331799000	-1.328832000	-0.114763000
6	3.671511000	-1.039072000	0.259122000
1	3.913047000	-0.063153000	0.666969000
6	4.690145000	-1.985523000	0.141833000
1	5.699145000	-1.717484000	0.449248000
6	4.428181000	-3.270773000	-0.349799000
1	5.223607000	-4.005251000	-0.443301000
6	3.111574000	-3.582595000	-0.721323000
1	2.879210000	-4.571912000	-1.110994000
6	2.093351000	-2.638726000	-0.615320000
1	1.082095000	-2.889406000	-0.915802000
6	-1.228149000	-0.365636000	-0.021634000
6	-1.559982000	1.062671000	-0.290743000
6	-1.011102000	1.738654000	-1.400681000
1	-0.314834000	1.209861000	-2.044595000
6	-1.345816000	3.062564000	-1.688366000
1	-0.906063000	3.551135000	-2.554691000
6	-2.243481000	3.761589000	-0.872049000
1	-2.502005000	4.793606000	-1.093885000
6	-2.799313000	3.111548000	0.235648000
1	-3.492723000	3.640265000	0.885943000
6	-2.462128000	1.785826000	0.517681000
1	-2.899206000	1.296766000	1.384288000
6	-2.331837000	-1.328791000	0.114776000
6	-2.093404000	-2.638712000	0.615278000
1	-1.082144000	-2.889420000	0.915723000
6	-3.111643000	-3.582560000	0.721276000

1	-2.879290000	-4.571900000	1.110896000
6	-4.428257000	-3.270692000	0.349805000
1	-5.223695000	-4.005157000	0.443306000
6	-4.690206000	-1.985421000	-0.141769000
1	-5.699209000	-1.717341000	-0.449138000
6	-3.671551000	-1.038986000	-0.259056000
1	-3.913084000	-0.063050000	-0.666859000

Compound: **5-mono**
Solvent: p-dioxane
Energy: -826.548589881 hartrees
Energy with thermal free energy correction: -826.297519 hartrees

7	0.472233000	0.493423000	0.008680000
6	-2.029970000	-4.010906000	0.013726000
1	-2.333284000	-5.053763000	0.012909000
6	-1.290935000	-3.494635000	1.082942000
1	-1.021295000	-4.134058000	1.918594000
6	-0.896835000	-2.154837000	1.081763000
1	-0.325256000	-1.759175000	1.916561000
6	-1.246494000	-1.304034000	0.017938000
6	-0.817501000	0.128252000	0.014823000
6	1.466739000	-0.365669000	-0.151312000
6	-3.746119000	3.269914000	0.049477000
1	-4.483517000	4.067176000	0.058771000
6	-4.131990000	1.948254000	0.296446000
1	-5.172138000	1.715051000	0.505689000
6	-3.187561000	0.922570000	0.281876000
1	-3.504427000	-0.093812000	0.488077000
6	-1.823596000	1.192770000	0.022032000
6	2.853174000	0.045866000	-0.086376000
6	3.868002000	-0.912863000	-0.310080000
1	3.588062000	-1.940362000	-0.528951000
6	5.213620000	-0.555814000	-0.254562000
1	5.979291000	-1.305995000	-0.430128000
6	5.578392000	0.766382000	0.026737000
1	6.626898000	1.045832000	0.070895000
6	4.582464000	1.728274000	0.252607000
1	4.862449000	2.754685000	0.472578000
6	3.237352000	1.377585000	0.198930000
1	2.464080000	2.117292000	0.375362000
6	-2.377403000	-3.177003000	-1.053427000
1	-2.948551000	-3.570587000	-1.889382000
6	-1.993972000	-1.833572000	-1.048565000
1	-2.269861000	-1.189909000	-1.878946000
6	-1.450898000	2.537148000	-0.221776000
1	-0.409061000	2.757865000	-0.422615000
6	-2.398163000	3.555795000	-0.208249000
1	-2.088434000	4.578760000	-0.403212000
1	1.279584000	-1.423828000	-0.356673000

Compound: **5-mono**
Solvent: Et_2O
Energy: -826.550240683 hartrees
Energy with thermal free energy correction: -826.299182 hartrees

7	-0.471836000	-0.495563000	0.010178000
6	2.026703000	4.011697000	0.010286000
1	2.329186000	5.054754000	0.008503000
6	1.288592000	3.495689000	1.080506000
1	1.018780000	4.135536000	1.915711000
6	0.895328000	2.155485000	1.080535000
1	0.324269000	1.760740000	1.916094000
6	1.245231000	1.304276000	0.016953000
6	0.817705000	-0.128377000	0.015116000
6	-1.467260000	0.363459000	-0.147921000
6	3.751564000	-3.266478000	0.051747000
1	4.490283000	-4.062445000	0.061700000
6	4.134887000	-1.943966000	0.299388000
1	5.174377000	-1.708944000	0.509628000
6	3.188592000	-0.919748000	0.283857000
1	3.503981000	0.097039000	0.490134000
6	1.825148000	-1.192008000	0.022451000
6	-2.853768000	-0.048600000	-0.084384000
6	-3.867942000	0.912347000	-0.303326000
1	-3.587317000	1.940690000	-0.516899000
6	-5.214067000	0.556130000	-0.249445000
1	-5.978946000	1.307963000	-0.421047000
6	-5.580232000	-0.767315000	0.025223000
1	-6.628954000	-1.045997000	0.068025000
6	-4.585092000	-1.731506000	0.246101000
1	-4.866009000	-2.758771000	0.460642000
6	-3.239442000	-1.381643000	0.194150000
1	-2.468013000	-2.124289000	0.366477000
6	2.374203000	3.177229000	-1.056677000
1	2.944547000	3.570585000	-1.893219000
6	1.991855000	1.833348000	-1.050551000
1	2.267967000	1.189778000	-1.880915000
6	1.455210000	-2.537229000	-0.222327000
1	0.414345000	-2.760919000	-0.425140000
6	2.404227000	-3.554600000	-0.207849000
1	2.096456000	-4.577964000	-0.403635000
1	-1.280771000	1.422052000	-0.350288000

Compound: **6**
Solvent: p-dioxane
Energy: -1057.60780728 hartrees
Energy with thermal free energy correction: -1057.282104 hartrees

7	0.000013000	-0.994264000	0.000443000
6	1.765655000	3.471557000	1.906998000
1	1.913564000	4.447011000	2.361000000
6	0.883433000	2.552206000	2.484497000
1	0.347230000	2.808667000	3.393726000
6	0.689336000	1.301444000	1.898259000
1	0.005874000	0.592235000	2.355465000
6	1.383477000	0.936291000	0.729665000
6	1.194292000	-0.420060000	0.143583000
6	-1.193962000	-0.420114000	-0.143384000
6	-1.383183000	0.936188000	-0.729620000
6	-0.689031000	1.301312000	-1.898196000
1	-0.005440000	0.592155000	-2.355295000
6	-0.883280000	2.551994000	-2.484562000
1	-0.347071000	2.808448000	-3.393789000
6	-1.765648000	3.471272000	-1.907179000
1	-1.913676000	4.446662000	-2.361282000
6	4.571786000	-2.902624000	-0.829793000
1	5.418754000	-3.535828000	-1.077280000
6	4.737646000	-1.799414000	0.014158000
1	5.714500000	-1.577897000	0.434688000
6	3.652754000	-0.981628000	0.330181000
1	3.799806000	-0.143473000	1.002036000
6	2.366371000	-1.249209000	-0.188611000
6	-2.456076000	3.125029000	-0.741680000
1	-3.137626000	3.834330000	-0.280749000
6	-2.273523000	1.867432000	-0.163542000
1	-2.812915000	1.609714000	0.742669000
6	-2.366374000	-1.249089000	0.188728000
6	-3.652352000	-0.981834000	-0.331126000
1	-3.798920000	-0.144122000	-1.003649000
6	-4.737465000	-1.799400000	-0.015258000
1	-5.714041000	-1.578211000	-0.436599000
6	-4.572154000	-2.901978000	0.829629000
1	-5.419303000	-3.534975000	1.077035000
6	-3.303080000	-3.183463000	1.350774000
1	-3.162401000	-4.036894000	2.008196000
6	-2.216658000	-2.371040000	1.036070000
1	-1.234485000	-2.590969000	1.440085000
6	2.456055000	3.125303000	0.741490000

1	3.137475000	3.834656000	0.280446000
6	2.273626000	1.867638000	0.163452000
1	2.812940000	1.609973000	-0.742824000
6	2.216137000	-2.371849000	-1.035040000
1	1.233704000	-2.592086000	-1.438244000
6	3.302337000	-3.184488000	-1.349856000
1	3.161212000	-4.038409000	-2.006551000

Compound: **PhI**
Solvent: p-Dioxane
Energy: -243.072782903 hartrees
Energy with thermal free energy correction: -243.014614 hartrees

6	1.265225000	-1.217463000	0.000000000
6	2.665052000	-1.208953000	0.000000000
6	3.366548000	0.000000000	0.000000000
6	2.665052000	1.208953000	0.000000000
6	1.265225000	1.217463000	0.000000000
6	0.582799000	0.000000000	0.000000000
1	0.725499000	-2.158074000	0.000000000
1	3.201379000	-2.153676000	0.000000000
1	4.452423000	0.000000000	0.000000000
1	3.201379000	2.153676000	0.000000000
1	0.725499000	2.158074000	0.000000000
53	-1.569162000	0.000000000	0.000000000

Compound: **PhI$^{•-}$**
Solvent: p-Dioxane
Energy: -243.146824097 hartrees
Energy with thermal free energy correction: -243.096371 hartrees

6	-1.904779000	1.219123000	-0.048632000
6	-3.309373000	1.214324000	0.018641000
6	-4.007405000	0.000074000	0.052489000
6	-3.309501000	-1.214256000	0.018648000
6	-1.904911000	-1.219198000	-0.048629000
6	-1.246465000	-0.000070000	-0.080850000
1	-1.356257000	2.157729000	-0.072556000
1	-3.854376000	2.156154000	0.046006000
1	-5.093233000	0.000126000	0.106366000
1	-3.854609000	-2.156023000	0.046021000
1	-1.356490000	-2.157864000	-0.072555000
53	2.068105000	-0.000002000	0.008995000

Compound: **AdI**
Solvent: Et_2O
Energy: -401.568030913 hartrees
Energy with thermal free energy correction: -401.370499 hartrees

6	-0.517937000	-0.504549000	1.365674000
1	-0.142296000	0.143569000	2.164890000
1	-0.142406000	-1.516619000	1.551677000
6	-0.047686000	-0.000047000	-0.000071000
6	-2.072248000	-0.503896000	1.364173000
1	-2.418630000	-0.864671000	2.340569000
6	-2.583374000	0.930891000	1.120155000
1	-2.238529000	1.597114000	1.921828000
1	-3.680847000	0.946626000	1.139036000
6	-0.517839000	1.434991000	-0.245887000
1	-0.142208000	1.803459000	-1.206675000
1	-0.142436000	2.101905000	0.537829000
6	-2.072144000	1.433454000	-0.245700000
1	-2.418553000	2.459404000	-0.421557000
6	-2.583033000	0.504715000	-1.366378000
1	-3.680512000	0.513080000	-1.389654000
1	-2.238000000	0.865924000	-2.344085000
6	-0.517743000	-0.930601000	-1.119900000
1	-0.142356000	-1.946924000	-0.958387000
1	-0.142268000	-0.585621000	-2.089396000
6	-2.583143000	-1.435664000	0.246095000
1	-2.238085000	-2.462970000	0.422269000
1	-3.680612000	-1.460072000	0.250274000
6	-2.071958000	-0.929394000	-1.118456000
1	-2.418447000	-1.594647000	-1.918998000
53	2.250355000	0.000019000	0.000040000

Compound: **Ad• I⁻**
Solvent: Et_2O
Energy: -401.672614491 hartrees
Energy with thermal free energy correction: -401.491832 hartrees

6	-8.727639000	0.896754000	1.261667000
1	-8.335874000	1.918390000	1.171096000
1	-8.476369000	0.526703000	2.264365000
6	-8.189387000	-0.006106000	0.181283000
6	-10.282128000	0.910250000	1.079143000
1	-10.737222000	1.555625000	1.842554000
6	-10.615095000	1.446171000	-0.331302000
1	-10.248343000	2.476036000	-0.439318000
1	-11.704478000	1.479181000	-0.469502000
6	-8.425529000	0.530587000	-1.207345000
1	-7.957474000	-0.101611000	-1.973290000
1	-8.030949000	1.548609000	-1.323056000
6	-9.977539000	0.541027000	-1.409036000
1	-10.216563000	0.924667000	-2.410089000
6	-10.510987000	-0.900911000	-1.255701000
1	-11.598320000	-0.914232000	-1.412095000
1	-10.069880000	-1.549339000	-2.024935000
6	-8.623923000	-1.440599000	0.340899000
1	-8.371357000	-1.834484000	1.334139000
1	-8.158034000	-2.093097000	-0.409195000
6	-10.814587000	-0.533030000	1.223451000
1	-10.590845000	-0.918532000	2.227504000
1	-11.907836000	-0.539085000	1.115365000
6	-10.177567000	-1.445379000	0.151425000
1	-10.558594000	-2.470117000	0.256960000
53	13.679782000	-0.000024000	0.000804000

Compound: **BIM**
Solvent: p-Dioxane
Energy: -685.881676570 hartrees
Energy with thermal free energy correction: -685.645112 hartrees

6	-2.836440000	0.671675000	0.396599000
6	-0.678792000	0.000000000	0.194888000
6	-2.836440000	-0.671675000	0.396599000
1	-3.661611000	1.357464000	0.522189000
1	-3.661611000	-1.357464000	0.522189000
6	0.678792000	0.000000000	0.194888000
6	2.836440000	-0.671675000	0.396599000
6	2.836440000	0.671675000	0.396599000
1	3.661611000	-1.357464000	0.522189000
1	3.661611000	1.357464000	0.522189000
7	-1.513237000	1.157950000	0.304048000
7	1.513237000	1.157950000	0.304048000
7	1.513237000	-1.157950000	0.304048000
7	-1.513237000	-1.157950000	0.304048000
6	-1.281453000	-2.341530000	-0.528333000
1	-1.260138000	-2.056264000	-1.596317000
1	-2.141613000	-3.001272000	-0.378411000
6	1.281453000	-2.341530000	-0.528333000
1	1.260138000	-2.056264000	-1.596317000
1	2.141613000	-3.001272000	-0.378411000
6	0.000000000	-3.096251000	-0.179269000
1	0.000000000	-3.355592000	0.886004000
1	0.000000000	-4.033032000	-0.750487000
6	1.281453000	2.341530000	-0.528333000
1	1.260138000	2.056264000	-1.596317000
1	2.141613000	3.001272000	-0.378411000
6	-1.281453000	2.341530000	-0.528333000
1	-1.260138000	2.056264000	-1.596317000
1	-2.141613000	3.001272000	-0.378411000
6	0.000000000	3.096251000	-0.179269000
1	0.000000000	4.033032000	-0.750487000
1	0.000000000	3.355592000	0.886004000

Compound: **BIM$^{•+}$**
Solvent: p-Dioxane
Energy: -685.748091054 hartrees
Energy with thermal free energy correction: -685.510443 hartrees

6	-2.854584000	0.674731000	0.179103000
6	-0.705252000	0.000000000	-0.053215000
6	-2.854584000	-0.674731000	0.179103000
1	-3.670675000	1.373102000	0.272515000
1	-3.670675000	-1.373102000	0.272515000
6	0.705252000	0.000000000	-0.053215000
6	2.854584000	-0.674731000	0.179103000
6	2.854584000	0.674731000	0.179103000
1	3.670675000	-1.373102000	0.272515000
1	3.670675000	1.373102000	0.272515000
7	-1.542449000	1.113918000	0.049013000
7	1.542449000	1.113918000	0.049013000
7	1.542449000	-1.113918000	0.049013000
7	-1.542449000	-1.113918000	0.049013000
6	-1.264473000	-2.507829000	-0.310157000
1	-1.210074000	-2.593430000	-1.405243000
1	-2.133501000	-3.078456000	0.021788000
6	1.264473000	-2.507829000	-0.310157000
1	1.210074000	-2.593430000	-1.405243000
1	2.133501000	-3.078456000	0.021788000
6	0.000000000	-3.075413000	0.315777000
1	0.000000000	-2.927521000	1.401163000
1	0.000000000	-4.155106000	0.131809000
6	1.264473000	2.507829000	-0.310157000
1	1.210074000	2.593430000	-1.405243000
1	2.133501000	3.078456000	0.021788000
6	-1.264473000	2.507829000	-0.310157000
1	-1.210074000	2.593430000	-1.405243000
1	-2.133501000	3.078456000	0.021788000
6	0.000000000	3.075413000	0.315777000
1	0.000000000	4.155106000	0.131809000
1	0.000000000	2.927521000	1.401163000

Compound: **BDMAP**
Solvent: p-Dioxane
Energy: -881.246092562 hartrees
Energy with thermal free energy correction: -880.905005 hartrees

6	0.680634000	0.480759000	-0.131880000
6	2.777192000	1.702537000	-0.371037000
6	3.533221000	0.591621000	-0.187284000
6	2.864901000	-0.703208000	-0.125172000
6	1.495676000	-0.725397000	-0.119438000
6	-0.680619000	0.480765000	0.131871000
6	-2.777155000	1.702569000	0.371027000
6	-3.533199000	0.591663000	0.187267000
6	-2.864904000	-0.703181000	0.125163000
6	-1.495675000	-0.725380000	0.119431000
1	3.233728000	2.675672000	-0.524804000
1	4.610961000	0.680578000	-0.190654000
1	0.979995000	-1.670982000	-0.042791000
1	-3.233682000	2.675708000	0.524790000
1	-4.610934000	0.680652000	0.190627000
1	-0.979996000	-1.670965000	0.042783000
7	3.666410000	-1.865401000	-0.152322000
7	-3.666423000	-1.865365000	0.152320000
6	-2.978188000	-3.140724000	0.277585000
1	-3.721291000	-3.928359000	0.429363000
1	-2.382923000	-3.401187000	-0.616678000
1	-2.311038000	-3.124065000	1.143585000
6	-4.766488000	-1.927471000	-0.812676000
1	-4.407541000	-2.134153000	-1.836418000
1	-5.455904000	-2.725426000	-0.521491000
1	-5.323542000	-0.990949000	-0.832901000
6	2.978151000	-3.140741000	-0.277652000
1	3.721243000	-3.928380000	-0.429455000
1	2.382874000	-3.401240000	0.616597000
1	2.311005000	-3.124032000	-1.143654000
6	4.766386000	-1.927529000	0.812776000
1	4.407348000	-2.134305000	1.836469000
1	5.455877000	-2.725426000	0.521606000
1	5.323381000	-0.990974000	0.833128000
7	-1.405684000	1.690156000	0.422188000
7	1.405719000	1.690139000	-0.422195000
6	-0.706192000	2.819020000	1.036737000
1	0.025095000	2.411034000	1.744545000
1	-1.425762000	3.402165000	1.618649000
6	0.706244000	2.819017000	-1.036734000

1	-0.025051000	2.411048000	-1.744542000
1	1.425820000	3.402157000	-1.618644000
6	0.000033000	3.699927000	0.000005000
1	-0.721464000	4.349635000	-0.510898000
1	0.721544000	4.349619000	0.510911000

Compound: **BDMAP$^{•+}$**
Solvent: p-Dioxane
Energy: -881.124260829 hartrees
Energy with thermal free energy correction: -880.782099 hartrees

6	0.712190000	0.472101000	-0.091942000
6	2.748343000	1.612452000	-0.651503000
6	3.514526000	0.517144000	-0.391149000
6	2.878422000	-0.716312000	0.003908000
6	1.485008000	-0.689002000	0.149605000
6	-0.712165000	0.472097000	0.091941000
6	-2.748324000	1.612437000	0.651504000
6	-3.514502000	0.517127000	0.391144000
6	-2.878396000	-0.716323000	-0.003913000
6	-1.484979000	-0.689008000	-0.149612000
1	3.195010000	2.543191000	-0.981331000
1	4.585918000	0.601477000	-0.499894000
1	0.963990000	-1.549607000	0.542258000
1	-3.194996000	2.543173000	0.981333000
1	-4.585897000	0.601449000	0.499885000
1	-0.963954000	-1.549601000	-0.542275000
7	3.619907000	-1.833362000	0.250252000
7	-3.619921000	-1.833346000	-0.250257000
6	-2.959362000	-3.070782000	-0.652371000
1	-3.698392000	-3.868405000	-0.711906000
1	-2.476445000	-2.973668000	-1.634098000
1	-2.200294000	-3.364768000	0.081729000
6	-5.081719000	-1.785603000	-0.205068000
1	-5.486092000	-1.068884000	-0.929725000
1	-5.473344000	-2.771207000	-0.452004000
1	-5.444034000	-1.519704000	0.794333000
6	2.959264000	-3.070743000	0.652390000
1	3.698236000	-3.868421000	0.711929000
1	2.476365000	-2.973577000	1.634121000
1	2.200163000	-3.364678000	-0.081697000
6	5.081711000	-1.785723000	0.205049000
1	5.486145000	-1.069023000	0.929692000
1	5.473263000	-2.771352000	0.452000000
1	5.444042000	-1.519874000	-0.794358000
7	-1.386716000	1.622604000	0.545694000
7	1.386736000	1.622613000	-0.545691000
6	-0.626733000	2.758940000	1.093369000
1	0.149100000	2.347334000	1.746897000
1	-1.300190000	3.345310000	1.720271000
6	0.626748000	2.758947000	-1.093360000

1	-0.149084000	2.347340000	-1.746889000
1	1.300201000	3.345322000	-1.720261000
6	0.000005000	3.628217000	0.000006000
1	-0.753875000	4.276409000	-0.459882000
1	0.753883000	4.276410000	0.459897000

References:

1. S.-J. Chen, H. Cai and Z.-L. Xue, *Organometallics*, 2009, **28**, 167-171.
2. Bruker, Bruker AXS, Inc, Madison, WI, USA, 2009.
3. Bruker, Bruker AXS, Inc, Madison, WI, USA, 2009.
4. G. M. Sheldrick, University of Gottingen, Germany, 2007.
5. G. M. Sheldrick, University of Gottingen, Germany,2008.
6. G. M. Sheldrick, University of Gottingen, Germany,, 2014.
7. P. E. Daniel, A. E. Weber and S. J. Malcolmson, *Org. Lett.*, 2017, **19**, 3490-3493.
8. K. Li, A. E. Weber, L. Tseng and S. J. Malcolmson, *Org. Lett.*, 2017, **19**, 4239-4242.
9. L. Liu, S. Zhang, X. Fu and C.-H. Yan, *Chem. Commun.*, 2011, **47**, 10148-10150.
10. A. E. Wendlandt and S. S. Stahl, *Org. Lett.*, 2012, **14**, 2850-2853.
11. M. Li, S. Berritt, L. Matuszewski, G. Deng, A. Pascual-Escudero, G. B. Panetti, M. Poznik, X. Yang, J. J. Chruma and P. J. Walsh, *J. Am. Chem. Soc.*, 2017, **139**, 16327-16333.
12. M. J. Frisch, G. W. Trucks, H. B. Schlegel, G. E. Scuseria, M. A. Robb, J. R. Cheeseman, G. Scalmani, V. Barone, G. A. Petersson, H. Nakatsuji, X. Li, M. Caricato, A. V. Marenich, J. Bloino, B. G. Janesko, R. Gomperts, B. Mennucci, H. P. Hratchian, J. V. Ortiz, A. F. Izmaylov, J. L. Sonnenberg, Williams, F. Ding, F. Lipparini, F. Egidi, J. Goings, B. Peng, A. Petrone, T. Henderson, D. Ranasinghe, V. G. Zakrzewski, J. Gao, N. Rega, G. Zheng, W. Liang, M. Hada, M. Ehara, K. Toyota, R. Fukuda, J. Hasegawa, M. Ishida, T. Nakajima, Y. Honda, O. Kitao, H. Nakai, T. Vreven, K. Throssell, J. A. Montgomery Jr., J. E. Peralta, F. Ogliaro, M. J. Bearpark, J. J. Heyd, E. N. Brothers, K. N. Kudin, V. N. Staroverov, T. A. Keith, R. Kobayashi, J. Normand, K. Raghavachari, A. P. Rendell, J. C. Burant, S. S. Iyengar, J. Tomasi, M. Cossi, J. M. Millam, M. Klene, C. Adamo, R. Cammi, J. W. Ochterski, R. L. Martin, K. Morokuma, O. Farkas, J. B. Foresman and D. J. Fox, Wallingford, CT, 2016.
13. S. F. Nelsen, S. C. Blackstock and Y. Kim, *J. Am. Chem. Soc.*, 1987, **109**, 677-682.
14. J. A. Murphy, S. z. Zhou, D. W. Thomson, F. Schoenebeck, M. Mahesh, S. R. Park, T. Tuttle and L. E. A. Berlouis, *Angew. Chem. Int. Ed.*, 2007, **46**, 5178-5183.
